# Acute mountain sickness prediction: a concerto of multidimensional phenotypic data and machine learning strategies in the framework of predictive, preventive, and personalized medicine

**DOI:** 10.1007/s13167-025-00404-9

**Published:** 2025-03-31

**Authors:** Wenhui Li, Meng Zhang, Yangyi Hu, Pan Shen, Zhijie Bai, Chaoji Huangfu, Zhexin Ni, Dezhi Sun, Ningning Wang, Pengfei Zhang, Li Tong, Yue Gao, Wei Zhou

**Affiliations:** 1 Research Center for High Altitude Medicine, Qinghai Provincial Key Laboratory of Plateau Medical Application, Key Laboratory of Ministry of Education, Qinghai-Utah Joint Research Key Laboratory for High Altitude Medicine, Qinghai University, Xining, 810000 China; 2 The Fifth People’s Hospital of Qinghai Province, Xining, 810000 China; 3 Department of Pharmaceutical Sciences, Beijing Institute of Radiation Medicine, Beijing, 100850 China; 4 Qinghai Provincial Key Laboratory of Traditional Chinese Medicine Research for Glucolipid Metabolic Diseases, Qinghai University, Xining, 810000 China; 5 State Key Laboratory of Kidney Diseases, Chinese PLA General Hospital, Beijing, 100853 China

**Keywords:** Predictive preventive personalized medicine (PPPM / 3PM), Acute mountain sickness (AMS), Proteomics, Metabolomics, Biomarker panel, Prediction model, Susceptibility, AMS predisposition, Machine learning, Artificial intelligence, Multi-omics, Multi-level diagnostics, Phenotyping, Support vector machine (SVM), Traditional Chinese Medicine, Acclimatization protocols, Tailored nutritional strategies, Stratified intervention, Lifestyle adjustments, Mitochondrial health

## Abstract

**Background:**

Acute mountain sickness (AMS) is a self-limiting illness, involving a complex series of physiological responses to rapid ascent to high altitudes, where the body is exposed to lower oxygen levels (hypoxia) and changes in atmospheric pressure. AMS is the mildest and most common form of altitude sickness; however, without adequate preparation and adherence to ascent guidelines, it can progress to life-threatening conditions.

**Aims:**

Due to the multi-factorial predisposition of AMS among individuals, identifying AMS biomarkers before high altitude exposure from multiple dimensions (e.g., clinical, metabolic, and proteomic markers) and integrating them to build an AMS predictive model enables early diagnosis and personalized interventions, which allows targeted allocation of medical resources, such as prophylactic medications (e.g., acetazolamide) and supplemental oxygen, to those who need them most and prevention of unnecessary complications. Consequently, predicting AMS utilizing biomarkers from multidimensional phenotypic data before high-altitude exposure is essential for the paradigm change in high-altitude medical research from currently applied reactive services to the cost-effective predictive, preventive, and personalized medicine (PPPM/3PM) in primary (reversible damage to health and targeted protection against health-to-disease transition) and secondary (personalized protection against disease progression) care.

**Methods:**

To this end, this study recruited 83 Han Chinese male volunteers and obtained clinical, proteomic, and metabolomic profiles for analysis before they ascended to high altitudes. The Mann–Whitney *U* test was used to identify clinical features distinguishing AMS from non-AMS. The proteomic and metabolomic features were concatenated and clustered to find co-expression modules associated with AMS. A machine learning model, Mutual Information-radial kernel-based Support Vector Machine-Recursive Feature Elimination (MI-radialSVM-RFE) was employed for biomarkers selection and AMS prediction. A molecular docking technique was used to select molecular biomarkers that can bind with Traditional Chinese Medicine (TCM) ingredients.

**Results:**

Among 83 participants, 66 were selected for detailed analysis after quality control steps. Six protein-metabolite co-expression modules were identified as significantly associated with AMS. The MI-radialSVM-RFE model selected 12 biomarkers (two clinical features: systolic blood pressure (SBP) and peak expiratory flow (PEF); six proteins: Acyl-CoA synthetase long-chain family member 4 (ACSL4), immunoglobulin kappa variable 1D-16 (IGKV1D-16), coagulation factor XIII B subunit (F13B), prosaposin (PSAP), poliovirus receptor (PVR), and multimerin-2 (MMRN2); and four metabolites: 2-Methyl-1,3-cyclohexadiene, calcitriol, 4-Acetamido-2-amino-6-nitrotoluene, and 20-Hydroxy-PGE2) for the AMS prediction model. The model exhibited excellent predictive performance in both training (*n* = 66) and validating cohorts (*n* = 24) with AUCs of 0.97 and 0.94, respectively. Additionally, molecular docking analysis suggested PSAP and ACSL4 proteins as potential molecular targets for AMS prevention.

**Conclusion and expert recommendations:**

This study advances high-altitude medicine by developing a predictive model for AMS using clinical, proteomic, and metabolomic data. The identified biomarkers linked to energy metabolism, immune response, and vascular regulation offer insights into AMS mechanisms. High-altitude predictive approaches should focus on implementing biomarker-driven risk screening using clinical, proteomic, and metabolomic data to identify high-risk individuals before high-altitude exposure. Preventive measures should prioritize pre-acclimatization protocols, tailored nutritional strategies and interventions guided by biomarker profiles, and lifestyle adjustments, such as maintaining mitochondrial health through proper nutritional strategies.

**Supplementary Information:**

The online version contains supplementary material available at 10.1007/s13167-025-00404-9.

## Introduction

Acute mountain sickness (AMS) is a self-limiting illness, involving a complex series of physiological responses to rapid ascent to high altitudes, where the body is exposed to lower oxygen levels (hypoxia) and changes in atmospheric pressure [1]. AMS is the mildest and most common form of altitude sickness; however, without adequate preparation and adherence to ascent guidelines, it can progress to high-altitude pulmonary edema (HAPE) [2] or high-altitude cerebral edema (HACE) [3], both of which are life-threatening. Depending on the altitude attained, individual susceptibility, rate of ascent, and degree of pre-acclimatization, the prevalence of AMS can be ~ 25–40% with a passive ascent to 3000–3500 m [4] and can increase to 40–90% among unacclimatized individuals ascending rapidly to altitudes of 4500–6000 m [5].

### The popularity of high-altitude activities highlights the impact of AMS

Driven by the increasing popularity of outdoor activities such as hiking, skiing, and mountaineering, mountain tourism has experienced significant growth. Although the COVID-19 pandemic caused a temporary decline in tourism, the sector has rebounded post-pandemic [6]. For instance, Andorra reported over 9 million tourists and 12 million overnight stays in 2023, demonstrating a strong recovery to pre-pandemic levels. Approximately 120 million tourists visit the mountainous regions of the Alps annually, and around 400 million skier-days (primarily downhill skiers and snowboarders) were recorded across 2000 ski areas in 80 countries worldwide in 2020 [7]. Notably, a significant proportion of these individuals are acutely exposed to altitudes above 2500 m, where the risk of developing mountain illnesses begins and increases progressively with higher elevations [8]. The growing popularity of high-altitude activities and the expansion of high-altitude economies highlight the widespread impact of AMS. This condition not only affects individual health, sports performance, and work efficiency but also has broader implications for community well-being, economic stability, and healthcare resource allocation.

### Physiological changes due to high altitude hypoxia cause AMS symptoms

The increasing level of hypoxemia due to the reduced inspired oxygen partial pressure with gain in altitude has long been recognized as the primary reason for developing AMS [7]. As altitude increases, atmospheric pressure decreases, leading to a lower partial pressure of oxygen (PaO_2_) and insufficient oxygen delivery to tissues [9]. The body initially responds by increasing the rate and depth of breathing (hyperventilation) to improve oxygen intake. However, hyperventilation reduces carbon dioxide (CO_2_) levels, potentially causing respiratory alkalosis [10]. To compensate, the kidneys excrete bicarbonate, gradually restoring pH balance [9]. Hypoxia also triggers vasodilation of cerebral blood vessels, increasing intracranial pressure [7], which is believed to contribute to headache, the hallmark symptom of AMS [11, 12]. In severe cases, hypoxia can disrupt the blood–brain barrier, allowing fluid to leak into the brain and causing cerebral edema, which manifests as dizziness, confusion, and nausea [13]. At high altitudes, low oxygen levels cause constriction of the pulmonary arteries (pulmonary vasoconstriction), which increases pulmonary artery pressure [14]. This can lead to pulmonary edema (fluid accumulation in the lungs), exacerbating hypoxia and causing shortness of breath, cough, and fatigue [15]. As the lungs respond to low oxygen, regions of the lung may not be adequately ventilated, leading to a mismatch between airflow and blood flow, further impairing gas exchange. As oxygen supply decreases, cells switch from aerobic to anaerobic metabolism, which produces lactic acid, leading to fatigue and muscle soreness [16].

On a molecular level, hypoxia may activate inflammatory responses to cope with altitude stress. Increased levels of inflammatory cytokines, such as IL-6 and TNF-α, have been observed in AMS sufferers, suggesting that inflammation plays a role in symptom manifestation [17]. Inflammation and hypoxia can lead to endothelial dysfunction, impairing vascular permeability and fluid balance, which may contribute to symptoms like edema in various tissues (e.g., face, hands, and lungs) [18]. Hypoxia-induced diuresis at high altitudes often results in fluid loss and dehydration [19], further worsening symptoms like headache and dizziness.

### Multidimensional insights into AMS predisposition: genetic, physiological, known phenotypes, and personalized approaches for prediction and prevention

The incidence of AMS varies significantly due to physiological, genetic, environmental, and behavioral factors. A faster ascent and higher altitude reduce the time available for acclimatization and increase hypoxic stress, thus increasing the risk of AMS [20]. Genetic variations, particularly in hypoxia-inducible factor (HIF) genes, influence the body’s ability to adapt to low oxygen levels [21, 22]. For instance, populations indigenous to high-altitude regions (e.g., Tibetans, Andeans) have genetic adaptations that confer protection against hypoxia [22]. Individuals with reduced ventilatory responses to hypoxia or less effective oxygen delivery mechanisms are more susceptible to AMS. Variations in how cerebral blood vessels respond to low oxygen (e.g., excessive vasodilation leading to increased intracranial pressure) can also influence AMS risk [23]. Conditions like insulin resistance or subclinical nutrient deficiencies (e.g., iron and vitamin D) may impair energy production and oxygen transport, increasing the risk of AMS [24]. Additionally, abnormal body weight represents a shifted metabolism that may also increase the risk of AMS [25].

Suboptimal health, defined as an intermediate state between health and disease, amplifies AMS risk and severity in various ways [26]. Individuals with reduced lung capacity or mild, undiagnosed respiratory conditions (e.g., mild asthma or chronic bronchitis) may struggle to adapt to hypoxia, increasing their risk of AMS [27]. Suboptimal cardiovascular health (e.g., hypertension, reduced cardiac output) impairs oxygen delivery to tissues, exacerbating the effects of low oxygen levels at altitude [28]. Suboptimal mitochondrial function or impaired oxidative metabolism can reduce the efficiency of oxygen utilization, leading to greater susceptibility to AMS [29]. Furthermore, suboptimal health is often associated with systemic inflammation. At high altitudes, hypoxia can trigger an inflammatory response, exacerbating symptoms like headache, nausea, and fatigue that are characteristic of AMS [30].

Ischemic stroke (IS), a severely under-diagnosed disease, shares common physiological pathways influenced by high-altitude exposure with AMS [31–33]. Hypoxia-induced changes, such as increased blood viscosity, a hypercoagulable state, and inflammatory responses, play pivotal roles in both conditions [34]. Contextually, people who are vulnerable to IS are also predisposed to AMS.

Flammer syndrome (FS), a phenotype of people with a predisposition for an altered blood vessel reaction to stimuli like coldness, emotional stress, or hypoxia, together with a group of signs and symptoms, has been known to be related to AMS [35]. As FS subjects are generally more sensitive, for example to high altitudes, symptoms of altitude sickness are more pronounced in them.

Stress overload, whether hormonal, psychological, or emotional, can lead to vasoconstriction and disrupted microcirculation [36]. This, in turn, contributes to systemic hypoxic-ischemic effects and prolonged oxidative stress. Individuals with impaired microcirculation often experience delayed adaptation to altitude changes, making them more prone to developing AMS.

Due to various AMS predispositions among individuals, identifying biomarkers from multiple dimensions (e.g., clinical, metabolic, and proteomic markers) linked to AMS risk enables early diagnosis and personalized interventions which allow targeted allocation of medical resources, such as prophylactic medications (e.g., acetazolamide) and supplemental oxygen, to those who need them most and prevention of unnecessary complications. Consequently, predicting AMS, utilizing biomarkers from multidimensional phenotypic data before high-altitude exposure, is essential for the paradigm change in high-altitude medical research from currently applied reactive services to the cost-effective predictive, preventive, and personalized medicine (PPPM/3PM) in primary (reversible damage to health and targeted protection against health-to-disease transition) and secondary (personalized protection against disease progression) care. To this end, an innovatively designed AMS biomarker selection approach and robust predictive model elaborated in this study play a crucial role in identifying AMS risk individuals (predictive), implementing tailored prevention measures (preventive), and customizing interventions (personalized).

### Predictive markers of AMS

Many efforts have been made to explore AMS indicators using different phenotypic data. Jia et al. distinguished between AMS-susceptible and AMS-resistant individuals through the analysis of plasma cytokine profiles at low altitude, pinpointing four key cytokines, i.e., IGFBP-6, SAA1, Dkk4, and IL-17RA, out of 75 differentially expressed ones as potential AMS susceptibility predictors [37]. Using a plasma metabolomic approach, Gao et al. identified significant alterations in 44 metabolites and four enzymes in subjects exposed to high altitudes for the first time, highlighting five metabolites (sphingomyelin, lecithin, glutamic acid, glyceric acid, and 12,13-diHOME) with predictive capabilities [38]. Additionally, proteomic approaches are also gaining traction in AMS research [39–42]. Guo et al., for instance, identified significant immunological and inflammatory differences between AMS-susceptible and AMS-resistant groups through proteomic comparisons [39]. He et al. profiled AMS symptoms, clinical indexes, and plasma proteomes of AMS, found the pathogenic role of RET to AMS, and suggested ADAM15, PHGDH, and TRAF2 as protective, predictive, and diagnostic biomarkers, respectively [40].

Despite these efforts, research on accurate models based on a holistic profile of individuals (including clinical, metabolic, and proteomic data) within the 3PM framework for AMS prediction before high-altitude exposure remains limited, significantly hampering early detection and intervention progress. To predict the likelihood of AMS before acute high-altitude exposure, we assessed the clinical, proteomic, and metabolomic profiles of a Chinese Han cohort (*n* = 83) before they ascended to the plateau and correlated them with AMS status after acute exposure. Co-expressed protein and metabolite modules were clustered and associated with AMS degrees and 26 clinical variables representing different phenotypes to elucidate the molecular mechanisms underlying AMS susceptibility. Next, employing a Mutual Information-radial kernel-based Support Vector Machine-Recursive Feature Elimination (MI-radialSVM-RFE) method, we identified key protein and metabolite biomarkers linked to AMS status. By integrating these molecular biomarkers with clinical indicators that differentiate AMS from non-AMS subjects, we developed a predictive model for AMS, which signifies a significant shift towards a proactive approach in high-altitude medical research. Furthermore, we explored their biological functions and potential as drug targets, offering insights into potential targeted AMS prevention strategies.

## The working hypothesis and aims

Considering the multi-factorial predisposition of AMS, we hypothesized by identifying and integrating multidimensional biomarkers, including clinical, proteomic, and metabolomic data, that it is possible to predict individual AMS predisposition prior to high-altitude exposure. A predictive model based on these biomarkers can transform AMS management by enabling proactive prediction and personalized interventions reducing the risk of severe altitude-related complications. Building on this concept, we developed the MI-radialSVM-RFE method to identify molecular features with high predictive power from multidimensional phenotypic data and construct an accurate AMS predictive model. This model is expected to facilitate rapid AMS screening before exposure to high-altitude environments.

## Volunteer recruitment and methodology

### Volunteer recruitment

We recruited Han Chinese male volunteers from Chengdu Medical College in Sichuan Province (Chengdu, China). The participants completed the questionnaire providing demographic information, including age, height, weight, and smoking habits, and accepted basic health examinations of blood pressure, heart rate, lung function, blood routine, and blood biochemistry. Peripheral venous blood samples were collected in ethylenediaminetetraacetic acid-coated collection vessels on an empty stomach before ascending to altitude, and plasma was centrifuged and stored at − 80 °C for analysis. Individuals enrolled should refer to the following criteria:

- the age above 18 years;
- no history of migraine, headache, and seizure;
- no history of plateau travel in the last year;
- no history of alcohol drinking in the last week.

Furthermore, individuals with a prior medical history of cardiovascular and respiratory disorders, regular prescription medication usage, and particularly acute infections were also excluded.

### AMS status measurement

**Fig. 1:**
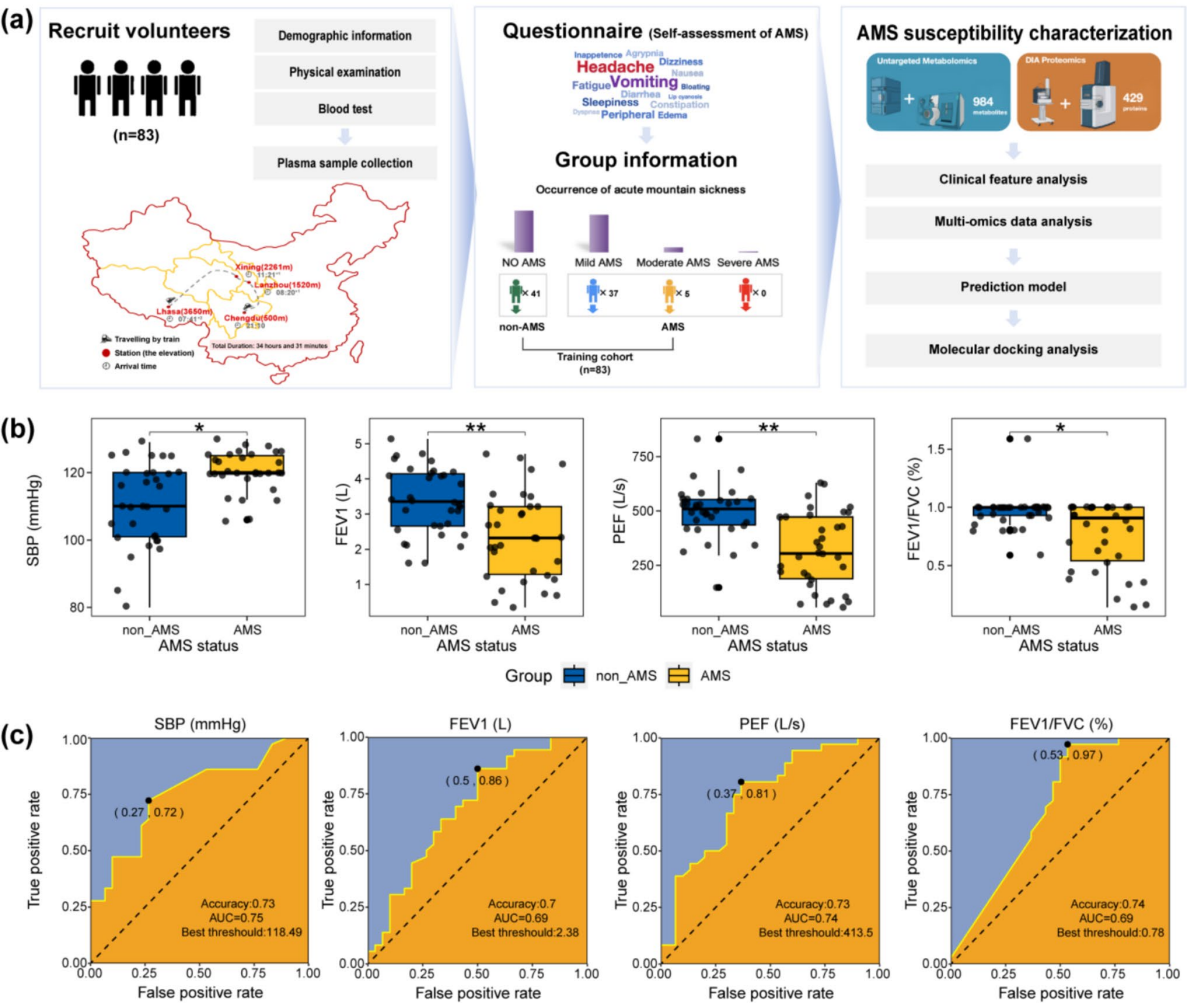
The clinical variables that distinguished AMS from non-AMS before high-altitude exposure. **a** The overall workflow of AMS prediction using multidimensional phenotypic data before high-altitude exposure. All subjects left Chengdu at 9:10 PM by train and arrived in Lhasa after 34 h and 31 min. **b** Four clinical variables showed significant differences between the non-AMS and AMS individuals, as determined by the Mann–Whitney *U* test. Asterisks indicate significant differences with *P*-values (* ≤ 0.05 and ** ≤ 0.01). **c** The area under the receiver operating characteristic curve (ROC AUC) of an AMS predictive model based on univariate logistic regression for each of the four clinical variables and the best thresholds. The ROC AUC was calculated on the training set (*n* = 66). Abbreviation: AMS, acute mountain sickness; SBP, systolic blood pressure; FEV1, forced expiratory volume in one second; PEF, peak expiratory flow; FVC, forced vital capacity

All subjects were transported to Lhasa (altitude of 3650 m) from Chengdu (altitude of 500 m; Fig. 1a indicates the locations of these two cities on the map) by train (the total journey lasted 34 h and 31 min). They were requested to complete the Lake Louise AMS self-questionnaire, which includes an assessment of various symptoms (headache, gastrointestinal symptoms, fatigue and/or weakness, and dizziness/light-headedness), on the first night after arriving at high altitude. Based on the Lake Louise Scoring (LLS) system, the diagnosis can be categorized into no AMS, mild AMS, moderate AMS, and severe AMS. Those diagnosed with no AMS were defined as the non-AMS group, and those with mild, moderate, and severe responses were defined as the AMS group.

### Preparation of samples for analysis

#### Proteomics

Prior to analysis, the samples undergo a series of pretreatments. Two microliter plasma from each specimen was denatured in 20 µL buffer containing 6 M urea in 100 mM ammonium bicarbonate (ABC) at 32 °C for 30 min. The proteins were reduced with 5 mM dithiothreitol (DTT) for 30 min at 32 °C, then alkylated for 45 min with 10 mM iodoacetamide (IAM) in darkness at room temperature. The protein extracts were diluted with 100 µL 50 mM ABC, and digested by trypsin with an enzyme-to-substrate ratio of 1:20, at 37 °C for 14 h. The reaction was stopped by adding 15 µL 10% trifluoroacetic acid (TFA) in volume. Digested peptides were cleaned up with SOLAµ (Thermo Fisher Scientific, identifier:62209–001) following the manufacturer’s instructions. Finally, 8 µg peptide mixtures were lyophilized and stored at − 80 °C for LC–MS/MS analysis. The lyophilized peptide sample was redissolved with buffer A (2% acetonitrile, 0.1% formic acid) for data-independent acquisition mass spectrometry (DIA-MS) analysis. Separation was performed on the Ultimate 3000 LC system (Thermo Fisher Scientific). The mobile phases consisted of phases A (2% acetonitrile/0.1% formic acid) and B (80% acetonitrile/0.1% formic acid). The peptide mixtures flowed through a homemade capillary column (150 µm inner diameter × 25 cm, 1.9 µm C18 particles, Dr. Maisch) at a steady rate of 600 nL/min and were separated with a 120 min nonlinear gradient. The peptides separated by the LC system were sprayed into the nanoESI source and then using an Orbitrap Fusion Lumos mass spectrometer (Thermo Fisher Scientific) for DIA analysis. DIA MS parameters were set to (1) MS: 350–1500 scan range (m/z); 120,000 resolution; 4e6 AGC target; 50 ms MIT; (2) HCD-MS/MS: 45 sequential precursor isolation windows; 30,000 resolutions; 200–2000 scan range (m/z); 4e6 AGC target; automatic MIT; Collision Energy (%): 32. DIA data was analyzed using Spectronaut. Then, based on the Target-decoy model applicable to DIA-MS, the FDR at PSM, peptide, and protein levels were controlled below 1%.

#### Metabolomics

One hundred microliter plasma mixed with 400 µL extraction solution (methanol:acetonitrile, 1:1(v/v)), the extraction solution contains deuterated internal standards. The suspension was vortexed for 30 s, sonicated for 10 min in a 4 °C water bath, and incubated for 1 h at − 40 °C to precipitate proteins. Then the samples were centrifuged at 12,000 rpm for 15 min at 4 °C, and the supernatant was transferred to a fresh glass vial for analysis. The quality control (QC) sample was prepared by mixing an equal aliquot of the supernatant of samples.

LC–MS/MS analyses were performed using an UHPLC system (Vanquish, Thermo Fisher Scientific) with a Phenomenex Kinetex C18 (2.1 mm × 50 mm, 2.6 µm) coupled to Orbitrap Exploris 120 mass spectrometer (Orbitrap MS, Thermo). The mobile phase A: 0.01% acetic acid in water; mobile phase B: isopropanol:acetonitrile (1:1(v/v)). The auto-sampler temperature was 4 °C, and the injection volume was 2 µL. The Orbitrap Exploris 120 mass spectrometer was used for its ability to acquire MS/MS spectra on information-dependent acquisition (IDA) mode in the control of the acquisition software (Xcalibur, Thermo). In this mode, the acquisition software continuously evaluates the full scan MS spectrum. The ESI source conditions were set as the following: sheath gas flow rate as 50 Arb, Aux gas flow rate as 15 Arb, capillary temperature 320 °C, full MS resolution as 60,000, MS/MS resolution as 15,000, collision energy: SNCE 20/30/40, spray voltage as 3.8 kV (positive) or − 3.4 kV (negative), respectively.

The raw data were converted to the mzXML format using ProteoWizard and processed with an in-house program, which was developed using R and based on XCMS, for peak detection, extraction, alignment, and integration. Then, an in-house MS2 database (BiotreeDB) was applied to metabolite annotation. The cutoff for annotation was set at 0.3.

### Preprocess of multi-omics data

#### Proteomics

We performed several preprocessing steps to ensure the quality of the proteomic data (Supplementary Fig. S1). First, for each sample, we considered protein abundances that differed by more than five standard deviations (SDs) from the mean protein abundance in the sample as “missing”. Next, we excluded those samples for which the fraction of missing proteins exceeded 20% (*n* = 13). Finally, we removed samples for which the distribution of protein abundance deviated from the overall protein abundance distribution with Kolmogorov–Smirnov (KS) distance > 0.08 (Q3 + 1.5IQR; Q3: the upper quartile and IQR: the interquartile range) (*n* = 2). Moreover, to ensure the reliability of the measured abundances for each protein, we also removed proteins with missing abundance in more than half (> 50%) of the remaining samples (*n* = 50). After filtering, 68 proteome profiles including 429 unique proteins were left and log2-transformed for the following analyses. Although intense quality control steps were performed, some proteins can still have missing values in some samples. Thus, we imputed the missing values using the Predictive Mean Matching (PMM) method from the “mice” R-package (version 3.16.0). After this, cyclic loess normalization from the “limma” R-package (version 3.58.1) was applied to normalize each pair of samples to each other.

#### Metabolomics

Similar preprocessing steps were performed to ensure the quality of metabolomic data (Supplementary Fig. S2). First, for each sample, we replaced metabolite abundances that differed by more than five SDs from the mean metabolite abundance in the sample with “missing”. Next, we removed samples for which the distribution of metabolite abundance deviated from the overall protein abundance distribution with KS-distance > 0.04 (Q3 + 1.5 IQR) (*n* = 2). No samples were excluded based on the criteria of the fraction of missing metabolites, as only limited missing values were observed in metabolomic profiles. Similarly, we removed metabolites with missing abundance in more than 10% of the remaining samples (*n* = 5). After filtering, 81 metabolome profiles including 984 unique metabolites were left and log2-transformed for the following analyses. Lastly, the same methods were used to impute the missing values and normalize each pair of samples to each other.

### Multi-omics data integration and clustering

To identify the co-expressed proteins and metabolites that might be able to explain the susceptibility to AMS, we integrated the proteomic and metabolomic data by concatenating the protein and metabolite abundances from samples that passed both proteomic and metabolomic data preprocessing steps (*n* = 66). Based on the integrated multi-omics data, hierarchical clustering was used to identify multi-omics co-expression modules. Specifically, the distance matrix was calculated using Spearman’s rank correlation coefficient and the clustering was performed by Ward’s method [43]. Next, the multi-omics modules were identified using dynamic tree cut (i.e., cutreeDynamic function in “cutreeDynamic” R-package, version 1.63–1) with minClusterSize = 45 to keep a relatively large module size. Moreover, the eigen-expression of each module was calculated using the moduleEigengenes function from the “WGCNA” R-package (version 1.72–1). To reduce the number of identified modules, we used mergeCloseModules function to merge similar modules with cutHeight = 0.4. Finally, the associations between modules and each clinical variable were calculated using the Pearson correlation coefficient, and the statistical significance was yielded with a *P* < 0.05 after multi-test correction using the “Benjamini & Hochberg” method.

### Functional enrichment analysis

To characterize the biological function of each module, we performed Gene Ontology (GO) and Kyoto Encyclopedia of Genes and Genomes (KEGG) enrichment analyses for proteins and metabolites separately. GO enrichment analysis of proteins was performed using the enrichGO function from the “clusterProfiler” R-package (version 4.10.0), and the statistical significance of the GO enrichment was tested using “Benjamini & Hochberg” with a cutoff *P* < 0.05. Furthermore, the redundant GO terms were removed using REVIGO with the semantic similarity measure “SimRel” and a cutoff of 0.5. KEGG enrichment analysis of metabolites was performed with MetaboAnalyst 6.0. All the KEGG analyses with *P* < 0.05 were enriched and shown.

### Feature selection of AMS prediction model

The AMS prediction model integrates clinical and molecular features. Specifically, the clinical features were selected from clinic variables that exhibited significant changes in the status of AMS, and the molecular features were selected from modules that showed significant associations with the degree of AMS. To maximize the predictive power of features, we employed a Mutual Information-radial kernel-based Support Vector Machine-Recursively Feature Elimination (MI-radialSVM-RFE) method, which was an extension of the classical SVM-RFE method [44, 45]. MI-radialSVM-RFE is an iterative algorithm based on mutual information, which starts with an initial set of features and effectively identifies the optimal hub of key proteins and metabolites linearly or nonlinearly associated with AMS by progressively eliminating feature vectors (see Supplementary Fig. S3 for details). The input data were from samples that have undergone both proteomic and metabolomic data preprocessing. This dataset contains 66 observations (individual subjects) and 619 features (proteins and metabolites). We set *k* = 3 for the k-fold cross-validation (CV) and halve.above = 100 to halve the feature set in each round until fewer than 100 features remained. Feature ranking was performed using the lapply function, based on the average rank across the three folds, with error estimation performed using a radialSVM. The process of feature selection and generalization error estimation involved varying number of top features from 1 to 30. Subsequently, the initially selected clinic, proteomic, and metabolomic features were tested using a univariate logistic regression. Only those features with significant predictive power (with a Bonferroni-corrected *P* < 0.05) were employed to build the final prediction model.

### Molecular docking analysis

Protein–ligand blind docking was performed using CB-Dock2 [46] with default parameters, which automatically calculates the curvature-based cavity detection to precisely narrow down the docking space as well as the optimized parameters for AutoDock Vina [47].

### Statistical analyses

The median with interquartile range (IQR) was used to indicate the distribution of each clinical variable, and the Mann–Whitney *U* test was applied to compare the differences between groups. The SHAP values (SHapley Additive exPlanations) [48] were calculated using kernelshap function from “kernelshap” R-package (version 0.4.1), and the bee swarm plot was made by shap.plot.summary function from “SHAPforxgboost” R-package (version 0.1.3). Decision curve analysis (DCA) [49] for the three single-type biomarker models (clinical, protein, and metabolite models) and the full model using all biomarkers was performed using decision_curve function in “rmda” R-package (version 1.6). Other R-packages including “ggplot2” (version 3.4.4), “cowplot” (version 1.1.1), “GOplot” (version 1.0.2), “amap” (version 0.8.19), “caret” (version 6.0.94), “e1071” (version 1.7.13), “pROC” (version 1.18.5), “ROCR” (version 1.0.11), and “ropls” (version 1.34.0) were applied in this study. The *P*-values were corrected using the “Benjamini & Hochberg” or “Bonferroni” method for multiple comparisons. All statistical analyses were performed using R (version 4.3.2).

## Results

### Sample characteristics

A total of 83 individuals were recruited based on our criteria, and their plasma samples were collected before they ascended to the plateau. All participants had their physiological responses monitored for 3 days after arriving at high altitude. AMS severities were assessed according to the Lake Louise Scoring (LLS) system 2018 on the first night. Among the 83 participants, 41 (49.40%) asymptomatic subjects were defined as the non-AMS group, while 37 (44.58%) subjects diagnosed with mild AMS, and 5 (6.02%) diagnosed with moderate AMS were defined as the AMS group. No severe AMS cases were observed in this study cohort. After performing quality control, 66 samples, consisting of 36 non-AMS individuals and 30 AMS individuals, were retained for the subsequent analyses; the clinical characteristics of these samples are shown in Table 1. Figure 1a summarizes the study setting and analysis process. To identify the clinical predictors of AMS, we performed the Mann–Whitney *U* test of all clinical variables in the 66 samples. The results revealed that apart from systolic blood pressure (SBP, *P* = 0.02), forced expiratory volume in one second (FEV1, *P* = 0.01), peak expiratory flow (PEF, *P* = 0.002), and ratio of forced expiratory volume in one second to forced vital capacity (FEV1/FVC, *P* = 0.02), no other variables significantly differed between the non-AMS and AMS individuals (Fig. 1b). When predicted using the area under the receiver operating characteristic curve (ROC AUC), SBP (AUC = 0.75), FEV1 (AUC = 0.69), PEF (AUC = 0.74), and FEV1/FVC (AUC = 0.69) performed well in distinguishing AMS from non-AMS (binary classifiers using logistic regression model, *n* = 66, Fig. 1c), suggesting blood pressure and lung function may be clinical phenotypic indicators of AMS susceptibility.

**Table 1:** Demographics and clinical characteristics of subjects

Variables	AMS	Non-AMS	Overall	Wilcoxon rank sum test / Fisher’s exact test ^a^	
	(*N* = 30)	(*N* = 36)	(*N* = 66)	*W*	*P*
Age (years)	*N* = 30	*N* = 36	*N* = 66		
Median [Q3–Q1]	23.0 [25.8–21.0]	22.5 [24.0–21.0]	23.0 [24.0–21.0]	599.0	0.45
BMI (kg/m^2^)	*N* = 29	*N* = 36	*N* = 65		
Median [Q3–Q1]	22.0 [23.0–21.0]	21.0 [24.0–21.0]	22.0 [24.0–21.0]	560.5	0.61
Smoke (%) ^a^	*N* = 24	*N* = 23	*N* = 47		
No	12 (40.0%)	14 (38.9%)	26 (39.4%)	/	0.56
Yes	12 (40.0%)	9 (25.0%)	21 (31.8%)		
SBP (mmHg)	*N* = 23	*N* = 25	*N* = 48		
Median [Q3–Q1]	120 [125–120]	116[120–109]	120 [125–112]	399.5	0.02^*^
DBP (mmHg)	*N* = 23	*N* = 25	*N* = 48		
Median [Q3–Q1]	75.0 [80.0–70.5]	76.0 [86.0–70.0]	76.0 [83.5–70.0]	242.0	0.35
HR (bpm)	*N* = 26	*N* = 31	*N* = 57		
Median [Q3–Q1]	73.0 [84.0–65.5]	80.0 [85.0–69.0]	76.0 [85.0–67.0]	357.5	0.47
SpO_2_ (%)	*N* = 26	*N* = 31	*N* = 57		
Median [Q3–Q1]	99.0 [99.0–98.0]	99.0 [99.0–97.5]	99.0 [99.0–98.0]	404.5	0.99
FVC (L)	*N* = 24	*N* = 30	*N* = 54		
Median [Q3–Q1]	3.63 [4.74–2.70]	3.29 [4.17–2.77]	3.46 [4.31–2.71]	382.0	0.71
FEV1 (L)	*N* = 24	*N* = 30	*N* = 54		
Median [Q3–Q1]	2.34 [3.21–1.86]	3.29 [4.09–2.67]	3.10 [3.79–2.13]	210.5	0.01^**^
PEF (L/s)	*N* = 24	*N* = 30	*N* = 54		
Median [Q3–Q1]	366 [484–212]	509 [553–423]	472 [530–320]	179.5	0.002^**^
FEV1/FVC	*N* = 24	*N* = 30	*N* = 54		
Median [Q3–Q1]	0.93 [1.00–0.57]	1.00 [1.00–0.93]	1.00 [1.00–0.83]	237.0	0.02^*^
WBC (E9/L)	*N* = 30	*N* = 36	*N* = 66		
Median [Q3–Q1]	5.70 [6.19–5.07]	6.07 [6.52–5.42]	5.97 [6.43–5.19]	437.0	0.19
NE (E9/L)	*N* = 30	*N* = 36	*N* = 66		
Median [Q3–Q1]	2.65 [3.35–2.28]	2.93 [3.44–2.40]	2.80 [3.42–2.33]	482.5	0.46
LY (E9/L)	*N* = 30	*N* = 36	*N* = 66		
Median [Q3–Q1]	2.23 [2.44–1.94]	2.40 [2.76–2.16]	2.32 [2.75–1.99]	450.5	0.25
RBC (E12/L)	*N* = 30	*N* = 36	*N* = 66		
Median [Q3–Q1]	5.31 [5.49–5.06]	5.28 [5.46–5.10]	5.30 [5.47–5.08]	513.0	0.73
HGB (g/L)	*N* = 30	*N* = 36	*N* = 66		
Median [Q3–Q1]	154 [161–148]	158 [162–152]	157 [162–150]	445.0	0.22
PLT (E9/L)	*N* = 30	*N* = 36	*N* = 66		
Median [Q3–Q1]	224 [254–198]	233 [250–209]	228 [251–200]	530.5	0.91
ALT (µkat/L)	*N* = 30	*N* = 36	*N* = 66		
Median [Q3–Q1]	0.27 [0.32–0.23]	0.27 [0.36–0.22]	0.27 [0.36–0.23]	536.0	0.96
AST (µkat/L)	*N* = 30	*N* = 36	*N* = 66		
Median [Q3–Q1]	0.31 [0.39–0.28]	0.33 [0.39–0.27]	0.33 [0.39–0.27]	563.5	0.77
TBIL (µmol/L)	*N* = 30	*N* = 36	*N* = 66		
Median [Q3–Q1]	14.6 [23.9–12.7]	16.9 [21.4–12.7]	15.5 [22.7–12.7]	523.0	0.83
UA (µmol/L)	*N* = 30	*N* = 36	*N* = 66		
Median [Q3–Q1]	366 [413–347]	384 [414–339]	374 [413–344]	496.0	0.58
UREA (mmol/L)	*N* = 30	*N* = 36	*N* = 66		
Median [Q3–Q1]	5.51 [6.37–4.62]	5.57 [6.27–4.73]	5.57 [6.35–4.68]	524.0	0.84
CREA (µmol/L)	*N* = 30	*N* = 36	*N* = 66		
Median [Q3–Q1]	77.2 [82.3–69.0]	76.3 [81.5–67.6]	76.5 [82.1–68.0]	572.5	0.68
CK-MB (µkat/L)	*N* = 30	*N* = 36	*N* = 66		
Median [Q3–Q1]	0.27 [0.34–0.24]	0.26 [0.32–0.22]	0.27 [0.33–0.22]	591.5	0.51
LDH (µkat/L)	*N* = 30	*N* = 36	*N* = 66		
Median [Q3–Q1]	2.94 [3.35–2.65]	2.99 [3.25–2.71]	2.96 [3.27–2.66]	519.5	0.80
GLU (mmol/L)	*N* = 30	*N* = 36	*N* = 66		
Median [Q3–Q1]	4.53 [4.92–4.25]	4.48 [4.84–4.11]	4.51 [4.88–4.16]	572.0	0.69

Abbreviation: BMI, body mass index; SBP, systolic blood pressure; DBP, diastolic blood pressure; HR, heart rate; SpO_2_, blood oxygen saturation; FVC, forced vital capacity; FEV1, forced expiratory volume in one second; PEF, peak expiratory flow; WBC, white blood cell count; NE, neutrophil count; LY, lymphocyte count; RBC, red blood cell count; HGB, hemoglobin; PLT, platelet; ALT, alanine aminotransferase; AST, aspartate aminotransferase; TBIL, total bilirubin; UA, uric acid; UREA, urea; CREA, creatinine; CK-MB, creatine kinase-MB; LDH, lactate dehydrogenase; GLU, blood glucose

^*^P < 0.05; ^**^P < 0.01

^a^Smoke was tested using Fisher’s exact test, and other variables were tested using the Wilcoxon rank sum test

### Multi-omics data revealed biological functions related to AMS susceptibility

**Fig. 2:**
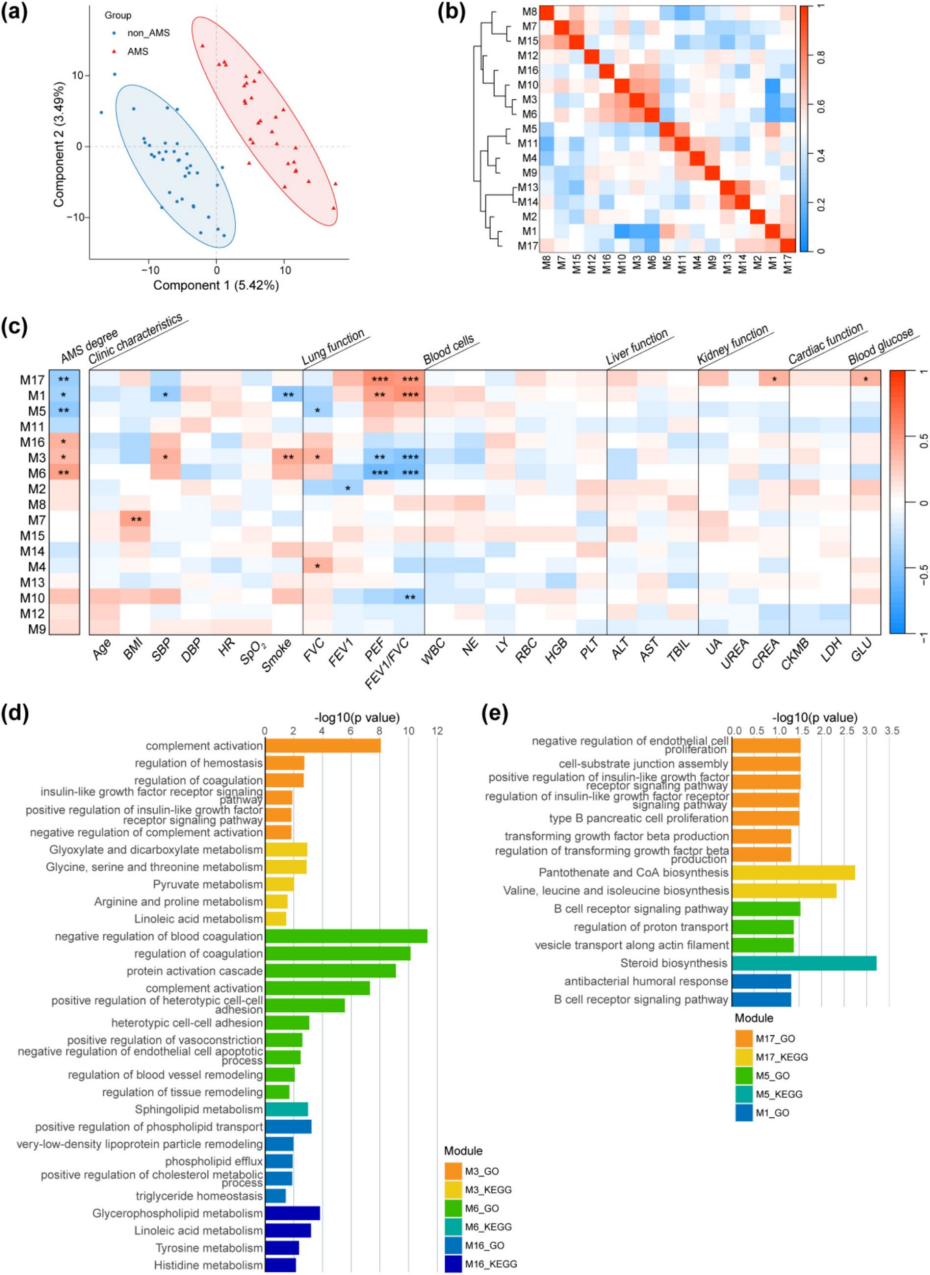
Protein-metabolite co-expression modules reveal potential links between pre-exposure phenotypes and AMS susceptibility. **a** Orthogonal partial least squares discriminant analysis (OPLS-DA) on the integrated proteomic and metabolomic data. **b** The dendrogram and correlation heatmap of the 17 identified protein-metabolite co-expression modules by hierarchical clustering. **c** Correlation heatmap between eigen-expression of each module and AMS degree after acute exposure and 26 clinical variables representing different phenotypes before acute exposure. Protein-metabolite co-expression modules were identified using hierarchical clustering with Spearman’s correlation coefficient. Asterisks indicate significant correlations with false discovery rates (FDR; * ≤ 0.05, ** ≤ 0.01, and *** ≤ 0.001). Gene Ontology (GO) and Kyoto Encyclopedia of Genes and Genomes (KEGG) enrichment analyses for proteins and metabolites in protein-metabolite co-expression modules that showed upregulation with AMS degree (**d**) and that showed downregulation with AMS degree (**e**). Abbreviation: AMS, acute mountain sickness; BMI, body mass index; SBP, systolic blood pressure; DBP, diastolic blood pressure; HR, heart rate; SpO_2_, blood oxygen saturation; FVC, forced vital capacity; FEV1, forced expiratory volume in one second; PEF, peak expiratory flow; WBC, white blood cell count; NE, neutrophil count; LY, lymphocyte count; RBC, red blood cell count; HGB, hemoglobin; PLT, platele; ALT, alanine aminotransferase; AST, aspartate aminotransferase; TBIL, total bilirubin; UA, uric acid; UREA, urea; CREA, creatinine; CKMB, creatine kinase-MB; LDH, lactate dehydrogenase; GLU, blood glucose

The complete and normalized proteomic (*n* = 429) and metabolomic (*n* = 984) abundances from each sample were concatenated to construct a multi-omics profile. Notably, integrated multi-omics data showed greater potential in distinguishing AMS from non-AMS using orthogonal partial least squares discriminant analysis (OPLS-DA) compared with single-omics data (Fig. 2a and Supplementary Fig. S4). Hierarchical clustering using Spearman’s correlation coefficient with Ward’s method identified 17 protein-metabolite co-expression modules (M1 ~ M17) with sizes ranging from 164 molecules (module M16) to 46 molecules (module M7) (Fig. 2b and Supplementary Data). Among the identified modules, six were significantly associated with AMS (namely AMS-modules), shown by correlating the eigen-expression of each module with AMS degree (Fig. 2c). Gene function enrichment analyses were performed separately for proteins and metabolites to explore the mechanistic association between clinical phenotypes and AMS susceptibility (Fig. 2d, e, and Supplementary Fig. S5). Significant correlations with lung function represented by FVC, FEV1, PEF, and FEV1/FVC were observed in five out of the six AMS-modules, suggesting lung function impairment could exacerbate AMS susceptibility by altering proteins or metabolites related to endothelial cells-, cell adhesion-, coagulation-, immune-, vesicle transport-, and energy metabolism-related biological processes (BPs) or pathways. Smoking and high SBP levels also appeared to influence AMS susceptibility by disturbing molecules involved in immune-related BPs (module M1 and M3), insulin-like growth factor (IGF) receptor-related BPs (module M1), and energy-related metabolism (module M1) [50, 51]. The same modules were observed to correlate with smoking and SBP levels indicating an association between SBP and smoking [52]. Additionally, higher levels of creatinine representing kidney function and blood glucose seem to contribute to AMS susceptibility through endothelial cells-, IGF receptor-, and transforming growth factor (TGF) beta-related BPs, and pantothenate and CoA biosynthesis and valine, leucine, and isoleucine biosynthesis related pathways (module M17). These results indicate that, while smoking, creatinine and blood glucose were not directly associated with AMS status, pre-dysregulated proteins in their shared biological functions may build a bridge.

### Establishment of the AMS prediction model

**Fig. 3:**
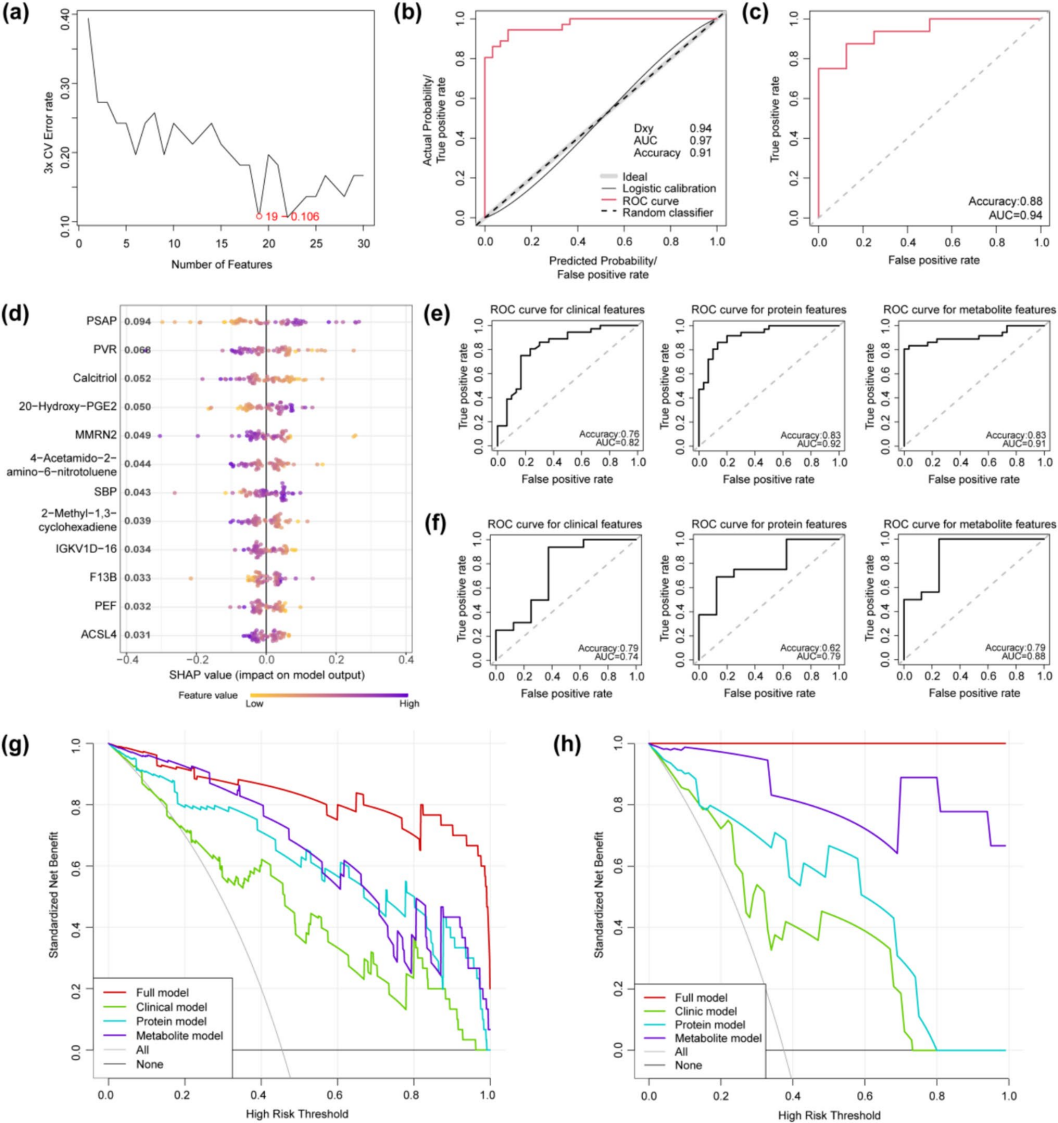
Establishing the predictive model of AMS. **a** The estimated prediction errors for a variety set of features (from top 1 to top 30) selected by MI-radialSVM-RFE. **b** The ROC curve and calibration curve of the AMS prediction model in the training cohort (*n* = 66). **c** ROC curve of the AMS prediction model in validating cohort (*n* = 24). **d** The bee swarm plot of SHAP values for selected biomarkers. **e** The ROC curve of the AMS prediction model using selected clinical, protein, and metabolite features in the training cohort. **f** The ROC curve of the AMS prediction model using selected clinical, protein, and metabolite features in the validating cohort. Decision curve analysis (DCA) for the three single-type biomarker models (clinical, protein, and metabolite models) and the full model using all biomarkers in the training cohort (**g**) and in the validating cohort (**h**). Abbreviation: PSAP, prosaposin; PVR, poliovirus receptor; 20-Hydroxy-PGE2, 20-hydroxy prostaglandin E2; MMRN2, multimerin-2; SBP, systolic blood pressure; IGKV1D-16, immunoglobulin kappa variable 1D-16; F13B, coagulation factor XIII B subunit; PEF, peak expiratory flow; ACSL4, acyl-CoA synthetase long-chain family member 4

To identify molecular biomarkers of AMS, we employed a novel machine learning model, MI-radialSVM-RFE, which wrapped around a feature selection metric as assessed by MI and a classification algorithm of radialSVM, iteratively removed the least important features from the high-dimensional feature set and obtained the optimum feature subset from various candidate subsets generated (Supplementary Fig. S3). The distinct advantage of employing MI in the MI-radialSVM-RFE model lies in its capability to capture and quantify the nonlinear correlations between molecular biomarkers and AMS, thereby enhancing the predictive power and accuracy of the radialSVM classifier in potential adverse medical conditions. The initial molecular feature set (*n* = 619) was from the six AMS-modules, and the lowest error rate was determined by the entire feature selection and error estimation process (three-fold cross-validation). After applying MI-radialSVM-RFE, we selected the top 19 ranked molecular features with the lowest classification error rate (Fig. 3a), combining them with the four clinical features that distinguished AMS from non-AMS (i.e., SBP, FEV1, PEF, and FEV1/FVC) to form a multidimensional phenotypic feature set. Twelve (12) (including 2 clinical, 6 proteomic, and 4 metabolomic features) of the 23 phenotypic features with significant predictive power (*n* = 66, univariate logistic regression test, *P* < 0.05 after family-wise error rate (FWER) correction) (Table S1) were selected as biomarkers to build the predictive model. The final AMS prediction model was trained using radialSVM on all 66 subjects (training cohort). To evaluate the performance of this model, an additional validating cohort (*n* = 35) traveling from Chengdu to Lhasa by train was recruited based on the same criteria as used for the training cohort, and their AMS status was evaluated using the LLS system as well. Moreover, the same QC steps were performed on the multi-omics data from the validating cohort, resulting in a final sample size of 24. The clinic characteristics of these 24 samples are shown in Table S2. In the training cohort (*n* = 66), the model achieved excellent performance for AMS prediction as analyzed using ROC (AUC = 0.97), accuracy (0.91), and calibration curve (Fig. 3b). When tested using ROC and accuracy in the validating cohort, this model was indicated with satisfactory predictive performance (AUC = 0.94 and accuracy = 0.88, Fig. 3c).

The importance of selected biomarkers on the prediction of the model was evaluated using SHAP values (SHapley Additive exPlanations) as shown in Fig. 3d. To evaluate the prediction ability of different phenotypic types (clinical, protein, and metabolite phenotypes), we trained and tested the radialSVM model using each phenotypic type separately. In the training cohort, a relatively good performance was achieved for each type (clinic phenotype: AUC = 0.82 and accuracy = 0.76; protein phenotype: AUC = 0.92 and accuracy = 0.83; and metabolite phenotype: AUC = 0.91 and accuracy = 0.83, Fig. 3e), but an apparent drop of performance was observed for the validating cohort (clinic phenotype: AUC = 0.74 and accuracy = 0.79; protein phenotype: AUC = 0.79 and accuracy = 0.62; and metabolite phenotype: AUC = 0.88 and accuracy = 0.79, Fig. 3f).

These results indicate that the multidimensional phenotypic features have stronger predictive potential and more generalized predictive ability. Additionally, to assess the clinical utilities of different phenotypic types, we build a logistic regression model using each phenotypic type separately. Decision curve analysis (DCA) showed that in both training and validating cohorts, proteomic and metabolomic biomarkers showed consistently higher positive net benefit (PNB) than clinical biomarkers for decision thresholds between 10 and 80%, and the full model using all biomarkers achieved the highest PNB for almost all decision thresholds (Fig. 3g, h).

### Biological functions of identified AMS biomarkers and their potential as drug targets

**Fig. 4:**
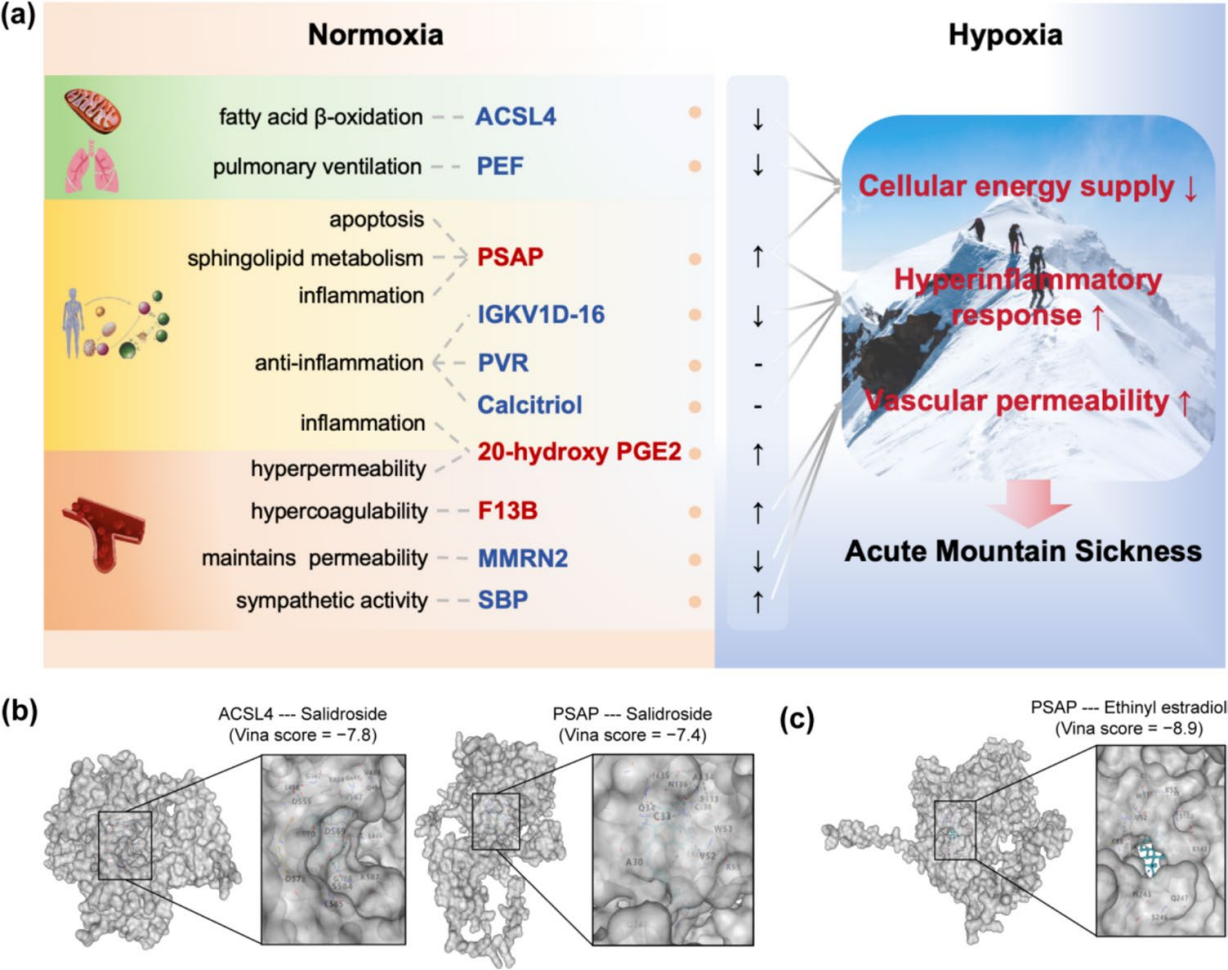
Potential relationships between the identified biomarkers and AMS and the related TCM ingredients. **a** The biological functions of the selected molecular biomarkers involve fatty acid metabolism, apoptosis, inflammatory response, and vascular permeability regulation and play a key role in hypoxia regulation. Imbalanced regulation can lead to insufficient cellular energy supply, increased inflammation, and increased vascular permeability, thereby promoting the occurrence of AMS. Additionally, this process is further exacerbated by the adverse effects of individual baseline metabolic changes, hypertension, and/or underlying pulmonary ventilatory dysfunction. Upregulated biomarkers in AMS are colored red, and downregulated biomarkers are colored blue. **b** Biomarkers related to AMS served (ACSL4 and PSAP) as potential targets that can be bound by the bioingredient salidroside in *Rhodiola rosea L*. Abbreviation: ACSL4, acyl-CoA synthetase long-chain family member 4; PEF, peak expiratory flow; PSAP, prosaposin; IGKV1D-16, immunoglobulin kappa variable 1D-16; PVR, poliovirus receptor; 20-Hydroxy-PGE2, 20-hydroxy prostaglandin E2; F13B, coagulation factor XIII B subunit; MMRN2, multimerin-2; SBP, systolic blood pressure

To investigate how the identified biomarkers are associated with AMS, we examined their biological functions. The findings suggest that these functions primarily relate to energy metabolism, immune responses, and vascular regulation (Fig. 4a), suggesting a possible link between the adaptive constraints of these functions and AMS susceptibility.

Next, to further validate the effectiveness of the identified molecular biomarkers, we explored their potential as drug targets for anti-AMS. For this, we investigated how AMS molecular biomarkers interact with the ingredients of *Rhodiola rosea L.*, a widely used Traditional Chinese Medicine (TCM) in Tibetan medicines for preventing and treating AMS for a long time [53]. Molecular docking analysis showed that the binding energies of salidroside from *Rhodiola rosea L.* to ACSL4 and PSAP were − 7.8 and − 7.4 (Fig. 4b). The 3D structure of ACSL4 (O60488) and PSAP (P07602) were obtained from the AlphaFold protein structure database [54], while the 3D structure of salidroside (CID 159278) was downloaded from the PubChem database. Salidroside was demonstrated to interact with 23 amino acid residues (SER441, GLY442, GLY443, ALA444, PRO445, LEU446, GLN464, GLY465, TYR466, GLY467, LEU468, VAL488, ASP555, ILE567, ILE568, ASP569, ARG570, ASP573, SER584, LEU585, GLY586, LYS587, and LYS690) in ACSL4 and with 16 amino acid residues (ALA30, CYS33, GLN34, ASN35, VAL52, TRP53, LYS55, SER133, ALA134, ASN136, CYS138, GLU139, SER140, GLN142, LYS143, and GLN247) in PSAP.

## Discussion and data interpretation

### Combining multidimensional phenotypic data with machine learning methods for proactive high-altitude medicine

This study presents a comprehensive approach to understanding and predicting AMS susceptibility by integrating clinical, proteomic, and metabolomic data. Using two clinical features (SBP and PEF) and ten molecular biomarkers (proteins: ACSL4, IGKV1D-16, F13B, PSAP, PVR, and MMRN2; metabolites: 2-Methyl-1,3-cyclohexadiene, calcitriol, 4-Acetamido-2-amino-6-nitrotoluene, and 20-Hydroxy-PGE2) selected by newly designed MI-radialSVM-RFE method, we established an AMS prediction model which demonstrated excellent predictive power on both training and validating cohorts. Among six protein biomarkers, ACSL4 and PSAP demonstrated the ability to bind salidroside from *Rhodiola rosea L.* a potential anti-hypoxic herb [55, 56], suggesting their capability as possible targets for preventing AMS. The innovative use of multidimensional phenotypical data and machine learning models to predict AMS susceptibility prior to high-altitude exposure in this study signifies a paradigm shift from reactive medicine to 3PM in the field of high-altitude medicine.

### Suboptimal baseline health, especially suboptimal respiratory function, associated with AMS

Physiological responses to hypoxia involving major organs are usually protective, but maladaptation in some individuals influenced by baseline health may contribute to the development of AMS. For example, in response to acute hypoxic exposure, the lungs respond with hypoxic ventilatory response (HVR) and hypoxic pulmonary vasoconstriction (HPV) to increase the rate and depth of breathing and redirect blood from poorly ventilated areas to well-ventilated areas and eventually optimize oxygen uptake [57]. However, the efficiency and extent of these adaptations may be affected by individual differences in lung function. Although all participants recruited in this study were healthy individuals, changes in clinical variables related to lung functions (i.e., FVC, FEV1, PEF, and FEV1/FVC) indicated their differences in pulmonary efficiency. The significant correlations between lung function-related clinical features (FEV1, PEF, and FEV1/FVC) and AMS status suggest individuals with compromised pulmonary efficiency may be more prone to AMS. This also aligns with the clinical observation that symptoms of AMS often include respiratory distress [58]. Apart from lung function-related clinical features, the level of SBP also differed between AMS and non-AMS subjects. Although observed in many studies [59], the direct link between high SBP and increased risk of AMS is still under investigation; the interplay of impaired cerebral blood flow regulation [60], endothelial dysfunction [61], increased oxidative stress [62], sympathetic overactivity [63], and altered renal response [64] could contribute to the heightened risk of AMS in individuals with high SBP.

### Selected AMS molecular biomarkers suggest a link between AMS predisposition and physiological limitations in energy metabolism, immune response, and vascular regulation

In most cases, baseline health differences associated with AMS susceptibility are not visible, which may explain the variance in using clinical phenotypic data to predict AMS across studies [65, 66]. The inadequacy of clinical phenotypes for AMS prediction provides room for molecular-based approaches to explain and predict AMS susceptibility. In fact, the selected molecular biomarkers using MI-radialSVM-RFE suggest a link between AMS susceptibility and physiological limitations in energy metabolism, immune response, and vascular regulation. Specifically, as an important regulator of fatty acid metabolism, ACSL4 promotes the conversion of free fatty acids into fatty acyl-CoA [67]. Downregulation of ACSL4 before high-altitude exposure leads to increased inhibition of fatty acid metabolism, which interacts with severe oxygen deficiency after high-altitude exposure and may cause various discomforts due to energy imbalance. Acute hypoxia triggers innate and adaptive immune responses which can link to four of the identified biomarkers, including PSAP which regulates monocyte and macrophage inflammation [68], IGKV1D-16, calcitriol, and PVR with anti-inflammatory properties [69, 70]. Hypoxia also drives angiogenesis [71], and MMRN2 competes with VEGF to bind VEGFR2, thereby inhibiting angiogenesis and weakening vascular permeability [72]. 20-hydroxy PGE2 is also a basic regulator of angiogenesis and participates in the regulation of pulmonary vascular homeostasis and remodeling [73]. Moreover, the elevation of hypoxia-inducible factor (HIF) 1-alpha levels and the induction of transferrin gene expression are well-established consequences of hypoxia. Increased transferrin carries iron to produce red blood cells in order to compensate for insufficient oxygen supply, while abnormally upregulated transferrin induces a hypercoagulable state [74]. This mechanism coincides with the high expression of F13B in this study, and activation of blood coagulation may lead to increased vascular permeability and may also amplify the inflammatory cascade [75, 76]. Notably, corresponding to our findings, Tibetans have higher ventilation, higher oxygen saturation, lower pulmonary artery pressure, and lower hemoglobin concentration compared with Han people, showing excellent altitude adaptability [22].

### The binding of active compound of anti-hypoxic herb to identified biomarkers suggests their potential as treatment and prevention targets for AMS

Several studies have indicated that *Rhodiola rosea L.* prevents and treats AMS by regulating the HIF-1 signaling pathway. Salidroside, a major biologically active compound of *Rhodiola rosea L.*, has been documented to exhibit various pharmacological activities, which may lay the material basis for its anti-hypoxic effects [77]. Salidroside can alleviate liver injury [78], lung injury [79, 80], and brain injury [81] caused by hypoxia through various pathways, significantly improving symptoms and restoring inflammatory factor balance triggered by hypoxia [79]. Notably, a close association between salidroside and inflammatory response was found in all of these studies. Furthermore, metabolic pathway enrichment analysis suggests that salidroside’s mechanisms against hypoxic injury may involve modulating the disordered homeostasis of energy and lipid metabolism [82]. These findings are consistent with the associations observed between salidroside and PSAP as well as ACSL4 in this study, indicating the identified biomarkers and their associated biological pathways can be molecular targets of TCM for AMS treatment and prevention [83].

### Limitations

Although establishing an AMS prediction model based on multi-omics data is the first attempt in the field of high-altitude medicine, the prediction accuracy of our model on both training and validation sets proves the feasibility of this approach. In fact, multi-omics data analysis is considered superior to single-omics analysis, as it offers a more holistic and comprehensive understanding of biological systems [84]. However, it is also important to acknowledge that the range of proteins and metabolites analyzed in this study may not encompass all underlying mechanisms contributing to AMS, given the variability in AMS incidence among individuals. Nevertheless, our research prioritized proteins with high expression levels, which enhances the clinical applicability of our findings. A limitation of the study is the exclusive inclusion of male participants, which restricts the investigation of sex-specific biomarkers and limits the applicability of our model to the female population. Another limitation is that this study focused only on the Han Chinese population. From the AMS predisposition point of view, future comparisons with other ethnic populations and including all sex groups will be necessary. Also, the correlations between hypoxia and the identified biomarkers necessitate further validation through extensive studies involving larger cohorts. Additionally, an in-depth exploration of the molecular mechanisms of AMS requires proteomic and transcriptomic analyses that investigate the temporal and spatial changes in protein homeostasis, including synthesis and degradation, within longitudinal cohort studies.

## Conclusion, expert recommendations, and outlook in the framework of 3PM

This study represents a significant advancement in the field of high-altitude medicine by successfully integrating clinical, proteomic, and metabolomic data to predict AMS within the 3PM framework. Using the innovative MI-radialSVM-RFE method, we identified critical clinical and molecular biomarkers linked to AMS and developed a robust predictive model. By training and validating on two separate cohorts, our model proved its accuracy in distinguishing AMS from non-AMS people. The identified AMS biomarkers highlight the potential interplay between energy metabolism, immune response, and vascular regulation in AMS predisposition, paving the way for predictive diagnostics and targeted interventions. Additionally, the binding of active compound, salidroside, from anti-hypoxic traditional herb to the identified AMS molecular biomarkers indicates their potential as preventive or therapeutic targets for anti-AMS.

To effectively manage AMS within the 3PM framework, adopting predictive, preventive, and personalized strategies is essential. Predictive approaches should focus on implementing biomarker-driven risk screening using clinical, proteomic, and metabolomic data to identify high-risk individuals before high-altitude exposure. Expanding multi-omics research, including transcriptomics and epigenomics, can further refine these predictions. Preventive measures should prioritize pre-acclimatization protocols, tailored nutritional strategies and interventions guided by biomarker profiles, and lifestyle adjustments. An important aspect is mitochondrial health quality control. At high altitudes, reduced oxygen availability challenges mitochondrial function, leading to decreased mitochondrial density and alterations in energy metabolism impairing the body’s ability to acclimatize and increase susceptibility to AMS [29]. To support mitochondrial health, several nutritional strategies can be focused on, such as prioritizing carbohydrate intake, consuming antioxidant-rich foods, proper hydration, and ensuring adequate iron and vitamin D [85, 86]. Also, incorporating traditional remedies like *Rhodiola rosea L.* based on molecular evidence offers additional preventive potential. Personalized medicine approaches can customize prophylactic and therapeutic strategies to individual risk profiles identified from multi-omics data, with a focus on real-time monitoring technologies for dynamic care adjustments. Expanding research to include female participants and diverse populations is crucial for ensuring the broad applicability of these strategies. Together, these recommendations aim to minimize AMS risk, enhance safety, and promote well-being in high-altitude environments.

The use of multi-omics data and machine learning represents a paradigm shift in AMS management from reactive to proactive. This integrative approach offers a more comprehensive understanding of AMS pathophysiology and allows for early identification and prevention.

### Future research directions

- Validation in diverse populations: Extend studies to larger, more diverse cohorts to validate the predictive model across different ethnicities, genders, and age groups.
- Longitudinal studies: Conduct longitudinal research to capture the temporal dynamics of biomarker expression and their association with AMS progression.
- Drug development: Leverage the identified biomarkers and pathways to develop novel pharmaceuticals or optimize existing therapies, such as salidroside-based treatments.
- Climate and environmental factors: Investigate the influence of changing environmental conditions, such as extreme cold or heat, on AMS susceptibility.

This study exemplifies the transformative potential of integrating predictive, preventive, and personalized strategies in high-altitude healthcare. By enabling early diagnosis, reducing morbidity, and tailoring interventions, the 3PM framework not only enhances individual health and performance but also contributes to the sustainability and safety of mountain tourism and high-altitude economics.

## Supplementary Information

Below is the link to the electronic supplementary material.

Supplementary file1 (DOCX 1.63 MB)

## Data Availability

The datasets generated during and/or analyzed during the current study are available from the corresponding author on reasonable request.

## Abbreviations

*AMS*: Acute mountain sickness
*20**-Hydroxy-PGE2*: 20-Hydroxy prostaglandin E2
*ABC*: Ammonium bicarbonate
*ACSL4*: Acyl-CoA synthetase long-chain family member 4
*ADAM15*: ADAM metallopeptidase domain 15
*ALT*: Alanine aminotransferase
*AST*: Aspartate aminotransferase
*AUC*: Area under the curve
*BMI*: Body mass index
*BPs*: Biological processes
*CK-MB*: Creatine kinase-MB
*CoA*: Coenzyme A
*CO*_*2*_: Carbon dioxide
*CREA*: Creatinine
*CV*: Cross-validation
*DBP*: Diastolic blood pressure
*DCA*: Decision curve analysis
*DIA-MS*: Data-independent acquisition mass spectrometry
*Dkk4*: Dickkopf WNT signaling pathway inhibitor 4
*DTT*: Dithiothreitol
*ESI*: Electrospray ionization
*F13B*: Coagulation factor XIII B subunit
*FDR*: False discovery rate
*FEV1*: Forced expiratory volume in one second
*FS*: Flammer syndrome
*FVC*: Forced vital capacity
*FWER*: Family-wise error rate
*GLU*: Blood glucose
*GO*: Gene Ontology
*HACE*: High altitude cerebral edema
*HAPE*: High altitude pulmonary edema
*HCD-MS/MS*: Higher energy collisional dissociation-tandem mass spectrometry
*HGB*: Hemoglobin
*HIF*: Hypoxia inducible factor
*HPV*: Hypoxic pulmonary vasoconstriction
*HR*: Heart rate
*HVR*: Hypoxic ventilatory response
*IAM*: Iodoacetamide
*IDA*: Information dependent acquisition
*IGF*: Insulin-like growth factor
*IGFBP-6*: Insulin-like growth factor binding protein 6
*IGKV1D-16*: Immunoglobulin kappa variable 1D-16
*IL-17RA*: Interleukin 17 receptor A
*IL-6*: Interleukin-6
*IQR*: Interquartile range
*IS*: Ischemic stroke
*KEGG*: Kyoto Encyclopedia of Genes and Genomes
*KS*: Kolmogorov-Smirnov
*LC*: Liquid chromatography
*LC**–**MS/MS*: Liquid chromatography-tandem mass spectrometry
*LDH*: Lactate dehydrogenase
*LLS*: Lake Louise Scoring
*LY*: Lymphocyte count
*MI*: Mutual information
*MI-radialSVM-RFE*: Mutual information-radial kernel-based support vector machine-recursive feature elimination
*MMRN2*: Multimerin-2
*MS*: Mass spectrometry
*MS/M**S **(MS2)*: Tandem mass spectrometry
*NE*: Neutrophil count
*OPLS-DA*: Orthogonal partial least squares discriminant analysis
*PaO*_*2*_: Pressure of oxygen
*PEF*: Peak expiratory flow
*pH*: Pondus hydrogenii
*PHGDH*: Phosphoglycerate dehydrogenase
*PLT*: Platelet
*PMM*: Predictive mean matching
*PNB*: Positive net benefit
*PPM/3PM*: Predictive, preventive, and personalized medicine
*PSAP*: Prosaposin
*PSM*: Propensity score matching
*PVR*: Poliovirus receptor
*QC*: Quality control
*radialSVM*: Radial kernel-based support vector machine
*RBC*: Red blood cell count
*RET*: Ret proto-oncogene
*ROC*: Receiver operating characteristic curve
*SAA1*: Serum amyloid A1
*SBP*: Systolic blood pressure
*SDs*: Standard deviations
*SHAP*: Shapley Additive exPlanations
*SpO*_*2*_: Blood oxygen saturation
*SVM-RFE*: Support vector machine-recursive feature elimination
*TBIL*: Total bilirubin
*TCM*: Traditional Chinese Medicine
*TFA*: Trifluoroacetic acid
*TGF*: Transforming growth factor
*TNF-α*: Tumor necrosis factor-alpha
*TRAF2*: TNF receptor-associated factor 2
*UA*: Uric acid
*UHPLC*: Ultra high performance liquid chromatography
*UREA*: Urea
*VEGF*: Vascular endothelial growth factor
*VEGFR2*: Vascular endothelial growth factor receptor 2
*WBC*: White blood cell count

## References

[1] Imray C, Wright A, Subudhi A, Roach R. Acute mountain sickness: pathophysiology, prevention, and treatment. Prog Cardiovasc Dis. 2010;52(6):467–84. 10.1016/j.pcad.2010.02.003. 20417340 10.1016/j.pcad.2010.02.003

[2] Bartsch P, Maggiorini M, Ritter M, Noti C, Vock P, Oelz O. Prevention of high-altitude pulmonary edema by nifedipine. N Engl J Med. 1991;325(18):1284–9. 10.1056/nejm199110313251805. 1922223 10.1056/NEJM199110313251805

[3] Hackett PH, Roach RC. High altitude cerebral edema. High Alt Med Biol. 2004;5(2):136–46. 10.1089/1527029041352054. 15265335 10.1089/1527029041352054

[4] Kriemler S, Bürgi F, Wick C, Wick B, Keller M, Wiget U, et al. Prevalence of acute mountain sickness at 3500 m within and between families: a prospective cohort study. High Alt Med Biol. 2014;15(1):28–38. 10.1089/ham.2013.1073. 24559431 10.1089/ham.2013.1073

[5] Kayser B, Dumont L, Lysakowski C, Combescure C, Haller G, Tramèr MR. Reappraisal of acetazolamide for the prevention of acute mountain sickness: a systematic review and meta-analysis. High Alt Med Biol. 2012;13(2):82–92. 10.1089/ham.2011.1084. 22724610 10.1089/ham.2011.1084

[6] Daniel S, Susanne B, Geoffrey L. Tourism and climate change stocktake 2023. Tourism panel on climate change. 2023. https://tpcc.info/

[7] Burtscher M, Hefti U, Hefti JP. High-altitude illnesses: old stories and new insights into the pathophysiology, treatment and prevention. Sport Med Heal Sci. 2021;3(2):59–69. 10.1016/j.smhs.2021.04.001. 10.1016/j.smhs.2021.04.001 PMC9219347 35782163

[8] Roach RC, Hackett PH, Oelz O, Bärtsch P, Luks AM, MacInnis MJ, et al. The 2018 Lake Louise acute mountain sickness score. High Alt Med Biol. 2018;19(1):4–6. 10.1089/ham.2017.0164. 29583031 10.1089/ham.2017.0164 PMC6191821

[9] Lee Hamm L, Nakhoul N, Hering-Smith KS. Acid-base homeostasis. Clin J Am Soc Nephrol. 2015;10(12):2232–42. 10.2215/CJN.07400715. 26597304 10.2215/CJN.07400715 PMC4670772

[10] Taylor A. High-altitude illnesses: physiology, risk factors, prevention, and treatment. Rambam Maimonides Med J. 2011;2(1):e0022. 10.5041/rmmj.10022. 23908794 10.5041/RMMJ.10022 PMC3678789

[11] Wu Y, Zhang C, Chen Y, Luo YJ. Association between acute mountain sickness (AMS) and age: a meta-analysis. Mil Med Res. 2018;5(1):1–8. 10.1186/s40779-018-0161-x. 29747689 10.1186/s40779-018-0161-x PMC5946480

[12] Alizadeh R, Ziaee V, Aghsaeifard Z, Mehrabi F, Ahmadinejad T. Characteristics of headache at altitude among trekkers; a comparison between acute mountain sickness and non- acute mountain sickness headache. Asian J Sports Med. 2012;3(2):126–30. 10.5812/asjsm.34714. 22942999 10.5812/asjsm.34714 PMC3426732

[13] Engelhardt S, Patkar S, Ogunshola OO. Cell-specific blood-brain barrier regulation in health and disease: a focus on hypoxia. Br J Pharmacol. 2014;171(5):1210–30. 10.1111/bph.12489. 24641185 10.1111/bph.12489 PMC3952799

[14] Dunham-Snary KJ, Wu D, Sykes EA, Thakrar A, Parlow LRG, Mewburn JD, et al. Hypoxic pulmonary vasoconstriction. Chest. 2017;151(1):181–92. 10.1016/j.chest.2016.09.001. 27645688 10.1016/j.chest.2016.09.001 PMC5310129

[15] Paralikar S. High altitude pulmonary edema-clinical features, pathophysiology, prevention and treatment. Indian J Occup Environ Med. 2012;16(2):59. 10.4103/0019-5278.107066. 23580834 10.4103/0019-5278.107066 PMC3617508

[16] Kumari P, Sengar MS, Sengar N. A mini review on lactic acidosis effect, cause, symptoms, complications, metabolism, and pathodology with its diagnosis, treatment, and preventions. J Chem Rev. 2024;6(1):27–38. 10.48309/jcr.2024.407092.1230.

[17] Guo H, Wang Q, Li T, Sun W, Chen J, Wang C, et al. IL-2, IL-17A and TNF-α hold potential as biomarkers for predicting acute mountain sickness prior to ascent. Cytokine. 2024;181:156694. 10.1016/j.cyto.2024.156694. 39024679 10.1016/j.cyto.2024.156694

[18] Ramakrishnan S, Anand V, Roy S. Vascular endothelial growth factor signaling in hypoxia and inflammation. J Neuroimmune Pharmacol. 2014;9(2):142–60. 10.1007/s11481-014-9531-7. 24610033 10.1007/s11481-014-9531-7 PMC4048289

[19] Swenson ER, Duncan TB, Goldberg SV, Ramirez G, Ahmad S, Schoene RB. Diuretic effect of acute hypoxia in humans: relationship to hypoxic ventilatory responsiveness and renal hormones. J Appl Physiol. 1995;78(2):377–83. 10.1152/jappl.1995.78.2.377. 7759405 10.1152/jappl.1995.78.2.377

[20] Savioli G, Ceresa IF, Gori G, Fumoso F, Gri N, Floris V, et al. Pathophysiology and therapy of high-altitude sickness: practical approach in emergency and critical care. J Clin Med. 2022;11(14). 10.3390/jcm11143937 10.3390/jcm11143937 PMC9325098 35887706

[21] Suzuki K, Kizaki T, Hitomi Y, Nukita M, Kimoto K, Miyazawa N, et al. Genetic variation in hypoxia-inducible factor 1α and its possible association with high altitude adaptation in Sherpas. Med Hypotheses. 2003;61(3):385–9. 10.1016/S0306-9877(03)00178-6. 12944107 10.1016/s0306-9877(03)00178-6

[22] Xu S, Li S, Yang Y, Tan J, Lou H, Jin W, et al. A genome-wide search for signals of high-altitude adaptation in Tibetans. Mol Biol Evol. 2011;28(2):1003–11. 10.1093/molbev/msq277. 20961960 10.1093/molbev/msq277

[23] Hoiland RL, Bain AR, Rieger MG, Bailey DM, Ainslie PN. Hypoxemia, oxygen content, and the regulation of cerebral blood flow. Am J Physiol Integr Comp Physiol. 2016;310(5):R398–413. 10.1152/ajpregu.00270.2015. 10.1152/ajpregu.00270.2015 PMC4796739 26676248

[24] Tangvarasittichai S. Oxidative stress, insulin resistance, dyslipidemia and type 2 diabetes mellitus. World J Diabetes. 2015;6(3):456. 10.4239/WJD.V6.I3.456. 25897356 10.4239/wjd.v6.i3.456 PMC4398902

[25] Golubnitschaja O, Liskova A, Koklesova L, Samec M, Biringer K, Büsselberg D, et al. Caution, “normal” BMI: health risks associated with potentially masked individual underweight—EPMA Position Paper 2021. EPMA J. 2021;12(3):243–64. 10.1007/s13167-021-00251-4. 34422142 10.1007/s13167-021-00251-4 PMC8368050

[26] Wang W, Yan Y, Guo Z, Hou H, Garcia M, Tan X, et al. All around suboptimal health — a joint position paper of the suboptimal health study consortium and European association for predictive preventive and personalised medicine. EPMA J. 2021;12(4):403–33. 10.1007/s13167-021-00253-2. 34539937 10.1007/s13167-021-00253-2 PMC8435766

[27] Stream JO, Luks AM, Grissom CK. Lung disease at high altitude. Expert Rev Respir Med. 2009;3(6):635–50. 10.1586/ers.09.51. 20477353 10.1586/ers.09.51 PMC4798974

[28] Fernandes IA, Rocha MP, Campos MO, Mattos JD, Mansur DE, Rocha HNM, et al. Reduced arterial vasodilatation in response to hypoxia impairs cerebral and peripheral oxygen delivery in hypertensive men. J Physiol. 2018;596(7):1167–79. 10.1113/JP275545. 29462837 10.1113/JP275545 PMC5878233

[29] Koklesova L, Mazurakova A, Samec M, Kudela E, Biringer K, Kubatka P, et al. Mitochondrial health quality control: measurements and interpretation in the framework of predictive, preventive, and personalized medicine. EPMA J. 2022;13(2):177–93. 10.1007/s13167-022-00281-6. 35578648 10.1007/s13167-022-00281-6 PMC9096339

[30] Pham K, Parikh K, Heinrich EC. Hypoxia and inflammation: insights from high-altitude physiology. Front Physiol. 2021;12:676782. 10.3389/fphys.2021.676782. 34122145 10.3389/fphys.2021.676782 PMC8188852

[31] Golubnitschaja O, Potuznik P, Polivka J, Pesta M, Kaverina O, Pieper CC, et al. Ischemic stroke of unclear aetiology: a case-by-case analysis and call for a multi-professional predictive, preventive and personalised approach. EPMA J. 2022;13(4):535–45. 10.1007/s13167-022-00307-z. 36415625 10.1007/s13167-022-00307-z PMC9670046

[32] Golubnitschaja O, Polivka J, Potuznik P, Pesta M, Stetkarova I, Mazurakova A, et al. The paradigm change from reactive medical services to 3PM in ischemic stroke: a holistic approach utilising tear fluid multi-omics, mitochondria as a vital biosensor and AI-based multi-professional data interpretation. EPMA J. 2024;15(1):1–23. 10.1007/s13167-024-00356-6. 38463624 10.1007/s13167-024-00356-6 PMC10923756

[33] Chen R, Wang X, Li N, Golubnitschaja O, Zhan X. Body fluid multiomics in 3PM-guided ischemic stroke management: health risk assessment, targeted protection against health-to-disease transition, and cost-effective personalized approach are envisaged. EPMA J. 2024;15(3):415–52. 10.1007/s13167-024-00376-2. 39239108 10.1007/s13167-024-00376-2 PMC11371995

[34] Polivka J, Polivka J, Pesta M, Rohan V, Celedova L, Mahajani S, et al. Risks associated with the stroke predisposition at young age: facts and hypotheses in light of individualized predictive and preventive approach. EPMA J. 2019;10(1):81–99. 10.1007/s13167-019-00162-5. 30984317 10.1007/s13167-019-00162-5 PMC6459458

[35] Konieczka K, Erb C. Diseases potentially related to Flammer syndrome. EPMA J. 2017;8(4):327–32. 10.1007/s13167-017-0116-4. 29209435 10.1007/s13167-017-0116-4 PMC5700007

[36] Kunin A, Sargheini N, Birkenbihl C, Moiseeva N, Fröhlich H, Golubnitschaja O. Voice perturbations under the stress overload in young individuals: phenotyping and suboptimal health as predictors for cascading pathologies. EPMA J. 2020;11(4):517–27. 10.1007/s13167-020-00229-8. 33200009 10.1007/s13167-020-00229-8 PMC7658305

[37] Lu H, Wang R, Li W, Xie H, Wang C, Hao Y, et al. Plasma cytokine profiling to predict susceptibility to acute mountain sickness. Eur Cytokine Netw. 2016;27(4):90–6. 10.1684/ecn.2016.0383. 28396299 10.1684/ecn.2016.0383

[38] Liao WT, Liu B, Chen J, Cui JH, Gao YX, Liu FY, et al. Metabolite modulation in human plasma in the early phase of acclimatization to hypobaric hypoxia. Sci Rep. 2016;6(1):1–14. 10.1038/srep22589. 26940428 10.1038/srep22589 PMC4778071

[39] Guo H, Wang Q, Li T, Chen J, Zhang C, Xu Y, et al. Potential plasma biomarkers at low altitude for prediction of acute mountain sickness. Front Immunol. 2023;14. 10.3389/fimmu.2023.1237465. 10.3389/fimmu.2023.1237465 PMC10569122 37841248

[40] Yang J, Jia Z, Song X, Shi J, Wang X, Zhao X, et al. Proteomic and clinical biomarkers for acute mountain sickness in a longitudinal cohort. Commun Biol. 2022;5(1). 10.1038/s42003-022-03514-6. 10.1038/s42003-022-03514-6 PMC9170681 35668171

[41] Sharma NK, Sethy NK, Bhargava K. Comparative proteome analysis reveals differential regulation of glycolytic and antioxidant enzymes in cortex and hippocampus exposed to short-term hypobaric hypoxia. J Proteomics. 2013;79:277–98. 10.1016/J.JPROT.2012.12.020. 23313218 10.1016/j.jprot.2012.12.020

[42] Julian CG, Subudhi AW, Hill RC, Wilson MJ, Dimmen AC, Hansen KC, et al. Exploratory proteomic analysis of hypobaric hypoxia and acute mountain sickness in humans. J Appl Physiol. 2014;116(7):937–44. 10.1152/japplphysiol.00362.2013. 24265281 10.1152/japplphysiol.00362.2013 PMC3972748

[43] Ward JH. Hierarchical grouping to optimize an objective function. J Am Stat Assoc. 1963;58(301):236–44. 10.1080/01621459.1963.10500845.

[44] Chen X-W. Gene selection for cancer classification using bootstrapped genetic algorithms and support vector machines. Comput Syst Bioinforma. 2003;46:504–5. 10.1109/CSB.2003.1227389.

[45] Sanz H, Valim C, Vegas E, Oller JM, Reverter F. SVM-RFE: selection and visualization of the most relevant features through non-linear kernels. BMC Bioinformatics. 2018;19(1):1–18. 10.1186/s12859-018-2451-4. 30453885 10.1186/s12859-018-2451-4 PMC6245920

[46] Liu Y, Yang X, Gan J, Chen S, Xiao Z-X, Cao Y. CB-Dock2: improved protein–ligand blind docking by integrating cavity detection, docking and homologous template fitting. Nucleic Acids Res. 2022;50(W1):W159–64. 10.1093/nar/gkac394. 35609983 10.1093/nar/gkac394 PMC9252749

[47] Liu Y, Grimm M, Dai WT, Hou MC, Xiao ZX, Cao Y. CB-Dock: a web server for cavity detection-guided protein–ligand blind docking. Acta Pharmacol Sin. 2019;41(1):138. 10.1038/S41401-019-0228-6. 31263275 10.1038/s41401-019-0228-6 PMC7471403

[48] Lundberg SM, Lee SI. A unified approach to interpreting model predictions. In: Proceedings of the 31st international conference on neural information processing systems. 2017;4768–77.

[49] Vickers AJ, Elkin EB. Decision curve analysis: a novel method for evaluating prediction models. Med Decis Mak. 2006;26(6):565–74. 10.1177/0272989X06295361. 10.1177/0272989X06295361 PMC2577036 17099194

[50] de la Monte SM, Tong M. Tobacco smoke-induced hepatic injury with steatosis, inflammation, and impairments in insulin and insulin-like growth factor signaling. J Clin Exp Pathol. 2016;06(02). 10.4172/2161-0681.1000269. 10.4172/2161-0681.1000269 PMC4979551 27525191

[51] Schutte AE, Volpe M, Tocci G, Conti E. Revisiting the relationship between blood pressure and insulin-like growth factor-1. Hypertension. 2014;63(5):1070–7. 10.1161/HYPERTENSIONAHA.113.03057. 24566078 10.1161/HYPERTENSIONAHA.113.03057

[52] Vallée A. Associations between smoking and alcohol consumption with blood pressure in a middle-aged population. Tob Induc Dis. 2023;21:1–14. 10.18332/tid/162440. 10.18332/tid/162440 PMC10193384 37215190

[53] Ou C, Geng T, Wang J, Gao X, Chen X, Luo X, et al. Systematically investigating the pharmacological mechanism of Dazhu Hongjingtian in the prevention and treatment of acute mountain sickness by integrating UPLC/Q-TOF-MS/MS analysis and network pharmacology. J Pharm Biomed Anal. 2020;179:113028. 10.1016/j.jpba.2019.113028. 31835126 10.1016/j.jpba.2019.113028

[54] Jumper J, Evans R, Pritzel A, Green T, Figurnov M, Ronneberger O, et al. Highly accurate protein structure prediction with AlphaFold. Nature. 2021;596(7873):583–9. 10.1038/s41586-021-03819-2. 34265844 10.1038/s41586-021-03819-2 PMC8371605

[55] Wang T, Hou J, Xiao W, Zhang Y, Zhou L, Yuan L, et al. Chinese medicinal plants for the potential management of high-altitude pulmonary oedema and pulmonary hypertension. Pharm Biol. 2020;58(1):815–27. 10.1080/13880209.2020.1804407. 32883127 10.1080/13880209.2020.1804407 PMC8641673

[56] Liang Z, Zhang X, Wang F, Zhang K, Liu H, Liu H. Understanding molecular mechanisms of Rhodiola rosea for the treatment of acute mountain sickness through computational approaches (a STROBE-compliant article). Medicine (Baltimore). 2018;97(39):e11886. 10.1097/MD.0000000000011886. 30278484 10.1097/MD.0000000000011886 PMC6181534

[57] Yang M, Wu Y, Yang XB, Liu T, Zhang Y, Zhuo Y, et al. Establishing a prediction model of severe acute mountain sickness using machine learning of support vector machine recursive feature elimination. Sci Rep. 2023;13(1):770–92. 10.1038/s41598-023-31797-0. 36944699 10.1038/s41598-023-31797-0 PMC10030784

[58] Smedley T, Grocott MP. Acute high-altitude illness: a clinically orientated review. Br J Pain. 2013;7(2):85–94. 10.1177/2049463713489539. 26516505 10.1177/2049463713489539 PMC4590130

[59] Chen R, Ye X, Sun M, Yang J, Zhang J, Gao X, et al. Blood pressure load: an effective indicator of systemic circulation status in individuals with acute altitude sickness. Front Cardiovasc Med. 2022;8:765422. 10.3389/fcvm.2021.765422. 35047574 10.3389/fcvm.2021.765422 PMC8761955

[60] Subudhi AW, Fan JL, Evero O, Bourdillon N, Kayser B, Julian CG, et al. AltitudeOmics: cerebral autoregulation during ascent, acclimatization, and re-exposure to high altitude and its relation with acute mountain sickness. J Appl Physiol. 2014;116(7):724–9. 10.1152/japplphysiol.00880.2013. 24371013 10.1152/japplphysiol.00880.2013

[61] Fan N, Liu C, Ren M. Effect of different high altitudes on vascular endothelial function in healthy people. Med (United States). 2020;99(11):E19292. 10.1097/MD.0000000000019292. 10.1097/MD.0000000000019292 PMC7220113 32176054

[62] Pena E, El Alam S, Siques P, Brito J. Oxidative stress and diseases associated with high-altitude exposure. Antioxidants. 2022;11(2):267. 10.3390/antiox11020267. 35204150 10.3390/antiox11020267 PMC8868315

[63] Hainsworth R, Drinkhill MJ, Rivera-Chira M. The autonomic nervous system at high altitude. Clin Auton Res. 2007;17(1):13–9. 10.1007/s10286-006-0395-7. 17264976 10.1007/s10286-006-0395-7 PMC1797062

[64] Wang S-Y, Gao J, Zhao J-H. Effects of high altitude on renal physiology and kidney diseases. Front Physiol. 2022;13. 10.3389/fphys.2022.969456. 10.3389/fphys.2022.969456 PMC9630589 36338473

[65] Avcil M, Yolcubal A, Özlüer YE, Yetiş Ç. Matrix metalloproteinase-9 and substance-P as predictors for early-stage diagnosis of acute mountain sickness. Am J Emerg Med. 2022;59:100–5. 10.1016/j.ajem.2022.07.001. 35820276 10.1016/j.ajem.2022.07.001

[66] Hayat A, Hussain MM, Aziz S, Siddiqui AH, Hussain T. Hyperventilatory capacity–a predictor of altitude sickness. J Ayub Med Coll Abbottabad. 2006;18(2):17–20. 16977807

[67] Ding K, Liu C, Li L, Yang M, Jiang N, Luo S, et al. Acyl-CoA synthase ACSL4: an essential target in ferroptosis and fatty acid metabolism. Chin Med J (Engl). 2023;136(21):2521–37. 10.1097/CM9.0000000000002533. 37442770 10.1097/CM9.0000000000002533 PMC10617883

[68] van Leent MMT, Beldman TJ, Toner YC, Lameijer MA, Rother N, Bekkering S, et al. Prosaposin mediates inflammation in atherosclerosis. Sci Transl Med. 2021;13(584). 10.1126/scitranslmed.abe1433. 10.1126/scitranslmed.abe1433 PMC8209679 33692130

[69] Nandi SS, Gohil T, Sawant SA, Lambe UP, Ghosh S, Jana S. CD155: a key receptor playing diversified roles. Curr Mol Med. 2021;22(7):594–607. 10.2174/1566524021666210910112906. 10.2174/1566524021666210910112906 34514998

[70] Li X, Xu S, Liu J, Zhao Y, Han H, Li X, et al. Treatment with 1,25-Dihydroxyvitamin D3 Delays choroid plexus infiltration and BCSFB injury in MRL/lpr mice coinciding with activation of the PPARγ/NF-κB/TNF-α pathway and suppression of TGF-β/Smad signaling. Inflammation. 2023;46(2):556–72. 10.1007/S10753-022-01755-5/FIGURES/9. 36269513 10.1007/s10753-022-01755-5

[71] Brat DJ, Van Meir EG. Glomeruloid microvascular proliferation orchestrated by VPF/VEGF. Am J Pathol. 2001;158(3):789–96. 10.1016/S0002-9440(10)64025-4. 11238026 10.1016/S0002-9440(10)64025-4 PMC1850366

[72] Lorenzon E, Colladel R, Andreuzzi E, Marastoni S, Todaro F, Schiappacassi M, et al. MULTIMERIN2 impairs tumor angiogenesis and growth by interfering with VEGF-A/VEGFR2 pathway. Oncogene. 2012;31(26):3136–47. 10.1038/onc.2011.487. 22020326 10.1038/onc.2011.487

[73] Ye L, Wang B, Xu H, Zhang X. The emerging therapeutic role of prostaglandin E2 signaling in pulmonary hypertension. Metabolites. 2023;13(11):1152. 10.3390/metabo13111152. 37999248 10.3390/metabo13111152 PMC10672796

[74] Li M, Tang X, Liao Z, Shen C, Cheng R, Fang M, et al. Hypoxia and low temperature upregulate transferrin to induce hypercoagulability at high altitude. Blood. 2022;140(19):2063–75. 10.1182/blood.2022016410. 36040436 10.1182/blood.2022016410 PMC10653030

[75] Cugno M, Marzano AV, Asero R, Tedeschi A. Activation of blood coagulation in chronic urticaria: pathophysiological and clinical implications. Intern Emerg Med. 2010;5(2):97–101. 10.1007/s11739-009-0333-5. 19949893 10.1007/s11739-009-0333-5

[76] Stadnicki A. Involvement of coagulation and hemostasis in inflammatory bowel diseases. Curr Vasc Pharmacol. 2012;10(5):659–69. 10.2174/157016112801784495. 22272910 10.2174/157016112801784495

[77] Yan X, Liu J, Zhu M, Liu L, Chen Y, Zhang Y, et al. Salidroside orchestrates metabolic reprogramming by regulating the Hif-1α signalling pathway in acute mountain sickness. Pharm Biol. 2021;59(1):1540–50. 10.1080/13880209.2021.1992449. 34739769 10.1080/13880209.2021.1992449 PMC8594887

[78] Xiong Y, Wang Y, Xiong Y, Teng L. Protective effect of salidroside on hypoxia-related liver oxidative stress and inflammation via Nrf2 and JAK2/STAT3 signaling pathways. Food Sci Nutr. 2021;9(9):5060–9. 10.1002/fsn3.2459. 34532015 10.1002/fsn3.2459 PMC8441355

[79] Wang Z, Guo Q, Ma J, Cheng J, Zhao A, Li W, et al. Protective effect of salidroside on lung tissue in rats exposed rapidly to high altitude. Zhejiang Da Xue Xue Bao Yi Xue Ban. 2022;51(4):422–9. 10.3724/ZDXBYXB-2022-0157. 37202094 10.3724/zdxbyxb-2022-0157 PMC10264988

[80] Song D, Zhao M, Feng L, Wang P, Li Y, Li W. Salidroside attenuates acute lung injury via inhibition of inflammatory cytokine production. Biomed Pharmacother. 2021;142:111949. 10.1016/j.biopha.2021.111949. 34325302 10.1016/j.biopha.2021.111949

[81] Jiang S, Fan F, Yang L, Chen K, Sun Z, Zhang Y, et al. Salidroside attenuates high altitude hypobaric hypoxia-induced brain injury in mice via inhibiting NF-κB/NLRP3 pathway. Eur J Pharmacol. 2022;925:175015. 10.1016/j.ejphar.2022.175015. 35561751 10.1016/j.ejphar.2022.175015

[82] Liao W, Liu J, Wang S, Xue Z, Zheng F, Feng F, et al. Metabolic profiling reveals that salidroside antagonizes hypoxic injury via modulating energy and lipid metabolism in cardiomyocytes. Biomed Pharmacother. 2020;122:109700. 10.1016/j.biopha.2019.109700. 31918273 10.1016/j.biopha.2019.109700

[83] Lu X, Jin Y, Wang Y, Chen Y, Fan X. Multimodal integrated strategy for the discovery and identification of quality markers in traditional Chinese medicine. J Pharm Anal. 2022;12(5):701–10. 10.1016/j.jpha.2022.05.001. 36320607 10.1016/j.jpha.2022.05.001 PMC9615540

[84] Wu C, Zhou F, Ren J, Li X, Jiang Y, Ma S. A selective review of multi-level omics data integration using variable selection. High-Throughput. 2019;8(1):4. 10.3390/ht8010004. 30669303 10.3390/ht8010004 PMC6473252

[85] Golubnitschaja O, Kapinova A, Sargheini N, Bojkova B, Kapalla M, Heinrich L, et al. Mini-encyclopedia of mitochondria-relevant nutraceuticals protecting health in primary and secondary care—clinically relevant 3PM innovation. EPMA J. 2024;15(2):163–205. 10.1007/s13167-024-00358-4. 38841620 10.1007/s13167-024-00358-4 PMC11148002

[86] Xiao Y, Xiao X, Zhang X, Yi D, Li T, Hao Q, et al. Mediterranean diet in the targeted prevention and personalized treatment of chronic diseases: evidence, potential mechanisms, and prospects. EPMA J. 2024;15(2):207–20. 10.1007/s13167-024-00360-w. 38841625 10.1007/s13167-024-00360-w PMC11147989

