# Supplementary material for: Acute mountain sickness prediction: a concerto of multidimensional phenotypic data and machine learning strategies in the framework of predictive, preventive, and personalized medicine
Source: EPMA J. 2025 Mar 31;16(2):265–84. doi: 10.1007/s13167-025-00404-9 (PMC12106293; doi:10.1007/s13167-025-00404-9)
Supplement: Supplementary file 1 — Supplementary file1 (DOCX 1.63 MB) [file 13167_2025_404_MOESM1_ESM.docx]

**Supplementary materials for:**

**Acute mountain sickness prediction: a concerto of multidimensional phenotypic data and machine learning strategies in the framework of predictive, preventive, and personalized medicine**

**Contents**

[1. Supplementary tables 1](#_Toc1255588956)

[Table S1. Result summary of the univariate logistic regression test for 23 selected features 1](#_Toc1509268070)

[Table S2. Demographics and Clinical Characteristics of the validating cohort. 2](#_Toc191614126)

[2. Supplementary figures 4](#_Toc1380628829)

[Fig. S1. Protein intensity distribution and missing value fraction in samples. 4](#_Toc667923168)

[Fig. S2. Metabolite intensity distribution and missing value fraction in samples. 5](#_Toc887661707)

[Fig. S3. The flowchart of the Mutual Information-radial kernel-based Support Vector Machine-Recursively Feature Elimination (MI-radialSVM-RFE) method. 6](#_Toc361413840)

[Fig. S4. Pot of orthogonal partial least squares discriminant analysis (OPLS-DA). 7](#_Toc1198655164)

[Fig. S5. Chord plot for enriched GO biological processes (BPs) and KEGG pathways. 8](#_Toc253248841)

[3. Supplementary Data 9](#_Toc40682333)

1. **Supplementary table****s**

**Table S1. Result summary of the univariate logistic regression test for 23 selected features**

| **Feature name** | **estimate** | **statistic** | **p-value** | **adjusted p-value** |
| --- | --- | --- | --- | --- |
| SBP | 1.124 | 3.241 | 0.001 | 0.027 |
| FEV1 | 0.463 | -2.626 | 0.009 | 0.199 |
| PEF | 0.993 | -3.068 | 0.002 | 0.05 |
| FEV1/FVC | 0.004 | -2.837 | 0.005 | 0.105 |
| ACSL4 | 0.337 | -3.4 | 0.001 | 0.015 |
| IGKV1D-16 | 0.423 | -3.117 | 0.002 | 0.042 |
| F13B | 48.17 | 3.073 | 0.002 | 0.049 |
| IGKV4-1 | 0.201 | -2.998 | 0.003 | 0.063 |
| CSF1R | 7.608 | 2.958 | 0.003 | 0.071 |
| PSAP | 3.285 | 4.169 | 0 | 0.001 |
| PVR | 0.146 | -3.503 | 0 | 0.011 |
| MMRN2 | 0.281 | -3.102 | 0.002 | 0.044 |
| N-Desmethylvenlafaxine | 0.046 | -2.945 | 0.003 | 0.074 |
| Linoleamide | 0.188 | -3.019 | 0.003 | 0.058 |
| 2-Methyl-1,3-cyclohexadiene | 0.03 | -3.499 | 0 | 0.011 |
| Germacr-1(10)-ene-5,8-dione | 0.113 | -2.183 | 0.029 | 0.668 |
| Phenylalanylphenylalanine | 0.349 | -2.826 | 0.005 | 0.108 |
| Homoarecoline | 0.005 | -3.034 | 0.002 | 0.056 |
| 4-Oxo-2-nonenal | 0.02 | -2.942 | 0.003 | 0.075 |
| Arginyl-Alanine | 7.69 | 2.915 | 0.004 | 0.082 |
| Calcitriol | 0.157 | -3.528 | 0 | 0.01 |
| 4-Acetamido-2-amino-6-nitrotoluene | 0.004 | -3.162 | 0.002 | 0.036 |
| 20-Hydroxy-PGE2 | 12.646 | 3.936 | 0 | 0.002 |

Abbreviation: SBP, systolic blood pressure; FEV1, forced expiratory volume in one second; FVC, forced vital capacity; PEF, peak expiratory flow; ACSL4, acyl-CoA synthetase long chain family member 4; IGKV1D-16, immunoglobulin kappa variable 1D-16; F13B, coagulation factor XIII B subunit; IGKV4-1, immunoglobulin kappa variable 4-1; CSF1R, macrophage colony-stimulating factor 1 receptor; PSAP, prosaposin; PVR, poliovirus receptor; MMRN2, multimerin-2; 20-Hydroxy-PGE2, 20-hydroxy prostaglandin E2

**Table S2. Demographics and Clinical Characteristics of the validating cohort.**

| Variables | AMS | Non-AMS | Overall | Wilcoxon rank sum test / Fisher's exact test ^a^ | |
| --- | --- | --- | --- | --- | --- |
|  | (N=9) | (N=15) | (N=24) | W | *P* |
| Age (years) | N=9 | N=15 | N=24 |  |  |
| Median [Q3-Q1] | 26.0 [29.0-24.0] | 23.0 [24.0-21.5] | 23.5 [26.0-22.8] | 101.5 | 0.04^*^ |
| BMI (Kg/m^2^) | N=9 | N=15 | N=24 |  |  |
| Median [Q3-Q1] | 23.0 [25.0-22.0] | 23.0 [24.0-22.0] | 23.0 [24.3.0-22.0] | 75.0 | 0.67 |
| Smoke (%) ^a^ | N=5 | N=14 | N=19 |  |  |
| No | 3 (33.3%) | 7 (46.7%) | 10 (41.7%) | / | 1.00 |
| Yes | 2 (22.2%) | 7 (46.7%) | 9 (37.5%) |  |  |
| SBP (mmHg) | N=7 | N=14 | N=21 |  |  |
| Median [Q3-Q1] | 120 [124-120] | 118 [120-110] | 120 [120-114] | 80.5 | 0.02^*^ |
| DBP (mmHg) | N=7 | N=14 | N=21 |  |  |
| Median [Q3-Q1] | 70.0 [76.0-69.0] | 74.0 [80.0-70.0] | 72.0 [80.0-70.0] | 31.0 | 0.18 |
| HR (bpm) | N=7 | N=15 | N=22 |  |  |
| Median [Q3-Q1] | 73.0 [73.5-70.0] | 79.0 [83.0-73.0] | 74.5 [80.75-71.5] | 26.5 | 0.07 |
| SpO_2_ (%) | N=7 | N=15 | N=22 |  |  |
| Median [Q3-Q1] | 99.0 [99.0-96.5] | 98.0 [99.0-97.0] | 98.0 [99.0-97.0] | 58.5 | 0.68 |
| FVC (L) | N=7 | N=15 | N=22 |  |  |
| Median [Q3-Q1] | 2.83 [4.39-1.62] | 3.61 [4.49-3.36] | 3.57 [4.52-2.86] | 38.0 | 0.32 |
| FEV1 (L) | N=7 | N=15 | N=22 |  |  |
| Median [Q3-Q1] | 2.83 [3.41-1.62] | 3.52 [3.98-3.04] | 3.39 [3.65-2.83] | 23.0 | 0.04^*^ |
| PEF (L/s) | N=7 | N=15 | N=22 |  |  |
| Median [Q3-Q1] | 386 [561-300] | 491 [530-414] | 485 [548-388] | 46.0 | 0.67 |
| FEV1/FVC | N=7 | N=15 | N=22 |  |  |
| Median [Q3-Q1] | 1.00 [1.00-0.78] | 1.00 [1.00-0.92] | 1.00 [1.00-0.88] | 47.0 | 0.70 |
| WBC (E9/L) | N=9 | N=15 | N=24 |  |  |
| Median [Q3-Q1] | 5.74 [6.81-5.10] | 6.28 [6.41-5.35] | 5.92 [6.52-5.18] | 66.0 | 0.95 |
| NE (E9/L) | N=9 | N=15 | N=24 |  |  |
| Median [Q3-Q1] | 2.72 [3.53-2.53] | 3.17 [3.94-2.56] | 3.14 [3.62-2.50] | 57.5 | 0.57 |
| LY (E9/L) | N=9 | N=15 | N=24 |  |  |
| Median [Q3-Q1] | 2.51 [2.67-2.28] | 2.25 [2.61-1.83] | 2.31 [2.64-1.98] | 93.0 | 0.14 |
| RBC (E12/L) | N=9 | N=15 | N=24 |  |  |
| Median [Q3-Q1] | 5.33 [5.64-5.26] | 5.34 [5.57-5.10] | 5.33 [5.61-5.18] | 78.5 | 0.53 |
| HGB (g/L) | N=9 | N=15 | N=24 |  |  |
| Median [Q3-Q1] | 161 [162-160] | 155 [158-153] | 157 [161-155] | 92.0 | 0.15 |
| PLT (E9/L) | N=9 | N=15 | N=24 |  |  |
| Median [Q3-Q1] | 217 [271-198] | 215 [284-197] | 216 [274-197] | 64.5 | 0.88 |
| ALT (μkat/L) | N=9 | N=15 | N=24 |  |  |
| Median [Q3-Q1] | 0.33 [0.42-0.22] | 0.28 [0.32-0.23] | 0.28 [0.36-0.23] | 77.0 | 0.59 |
| AST (μkat/L) | N=9 | N=15 | N=24 |  |  |
| Median [Q3-Q1] | 0.35 [0.37-0.31] | 0.32 [0.35-0.31] | 0.32 [0.36-0.31] | 81.0 | 0.44 |
| TBIL (μmol/L) | N=9 | N=15 | N=24 |  |  |
| Median [Q3-Q1] | 16.0 [26.3-13.0] | 13.7 [16.5-11.4] | 14.4 [18.1-12.1] | 40.5 | 0.11 |
| UA (μmol/L) | N=9 | N=15 | N=24 |  |  |
| Median [Q3-Q1] | 377 [392-350] | 368 [429-357] | 373 [411-353] | 72.0 | 0.81 |
| UREA (mmol/L) | N=9 | N=15 | N=24 |  |  |
| Median [Q3-Q1] | 5.10 [5.43-4.92] | 5.14 [5.83-4.48] | 5.11 [5.74-4.69] | 61.0 | 0.73 |
| CREA (μmol/L) | N=9 | N=15 | N=24 |  |  |
| Median [Q3-Q1] | 69.2 [76.6-65.8] | 78.4 [84.7-75.5] | 77.1 [79.2-70.3] | 111.0 | 0.01^*^ |
| CK-MB (μkat/L) | N=9 | N=15 | N=24 |  |  |
| Median [Q3-Q1] | 0.25 [0.29-0.20] | 0.23 [0.27-0.20] | 0.24 [0.28-0.20] | 76.0 | 0.63 |
| LDH (μkat/L) | N=9 | N=15 | 24 |  |  |
| Median [Q3-Q1] | 3.03 [3.38-2.48] | 2.87 [3.05-2.76] | 2.90 [3.16-2.65] | 73.5 | 0.74 |
| GLU (mmol/L) | N=9 | N=15 | N=24 |  |  |
| Median [Q3-Q1] | 4.27 [4.55-3.99] | 4.23 [4.80-3.94] | 4.25 [4.80-3.95] | 69.0 | 0.95 |

Abbreviation: BMI, body mass index; SBP, systolic blood pressure; DBP, diastolic blood pressure; HR, heart rate; SpO_2_, blood oxygen saturation; FVC, forced vital capacity; FEV1, forced expiratory volume in one second; PEF, peak expiratory flow; WBC, white blood cell count; NE, neutrophil count; LY, lymphocyte count; RBC, red blood cell count; HGB, hemoglobin; PLT, platelet; ALT, alanine aminotransferase; AST, aspartate aminotransferase; TBIL, total bilirubin; UA, uric acid; UREA, urea; CREA, creatinine; CK-MB, creatine kinase-MB; LDH, lactate dehydrogenase; GLU, blood glucose

^*^*P* < 0.05

^a^ Smoke was tested using Fisher’s exact test, and other variables were tested using the Wilcoxon rank sum test

1. **Supplementary figures**


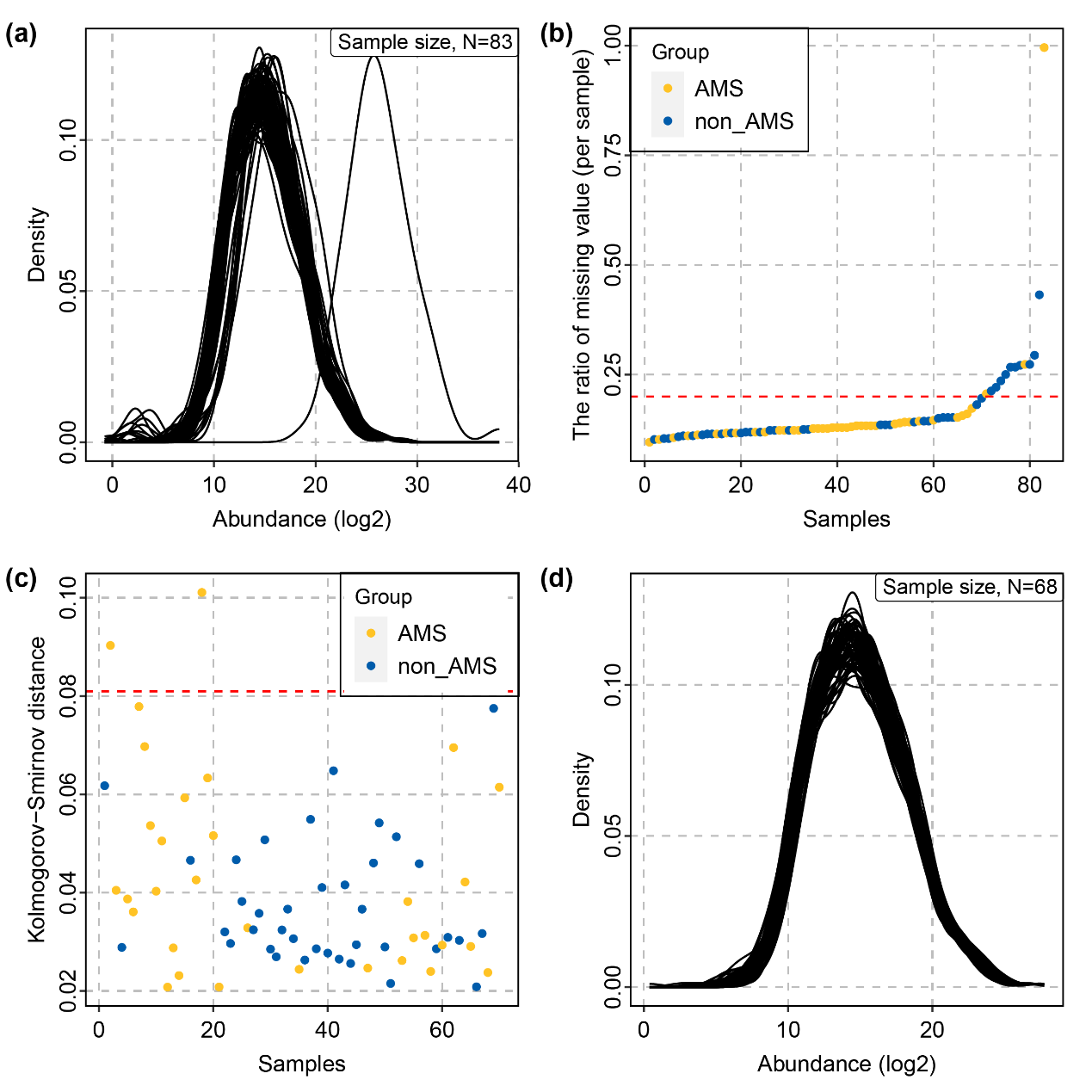


## **Fig. S1** Protein intensity distribution and missing value fraction in samples. a 479 unique proteins were measured in 83 samples. A density distribution with Gaussian kernel was used to estimate the protein abundance distribution in each sample. b Protein abundance was measured in plasma from non-AMS (blue dot, n = 41) and AMS (yellow dot, n = 42) individuals. The fraction of proteins with missing value in each sample was plotted in an increasing order. 13 samples with the fraction of missing proteins exceeded 20% were removed. **c** The distances between single sample protein abundance distribution and overall protein abundance distribution were calculated using the “Kolmogorov-Smirnov” test. The threshold of 0.081 was calculated using Q3 + 2 IQR. 2 samples above the threshold were excluded from the following analyses. **d** The proteomic profiles from 68 samples were retained after quality control, and the density distributions were estimated using the same method as in (a).


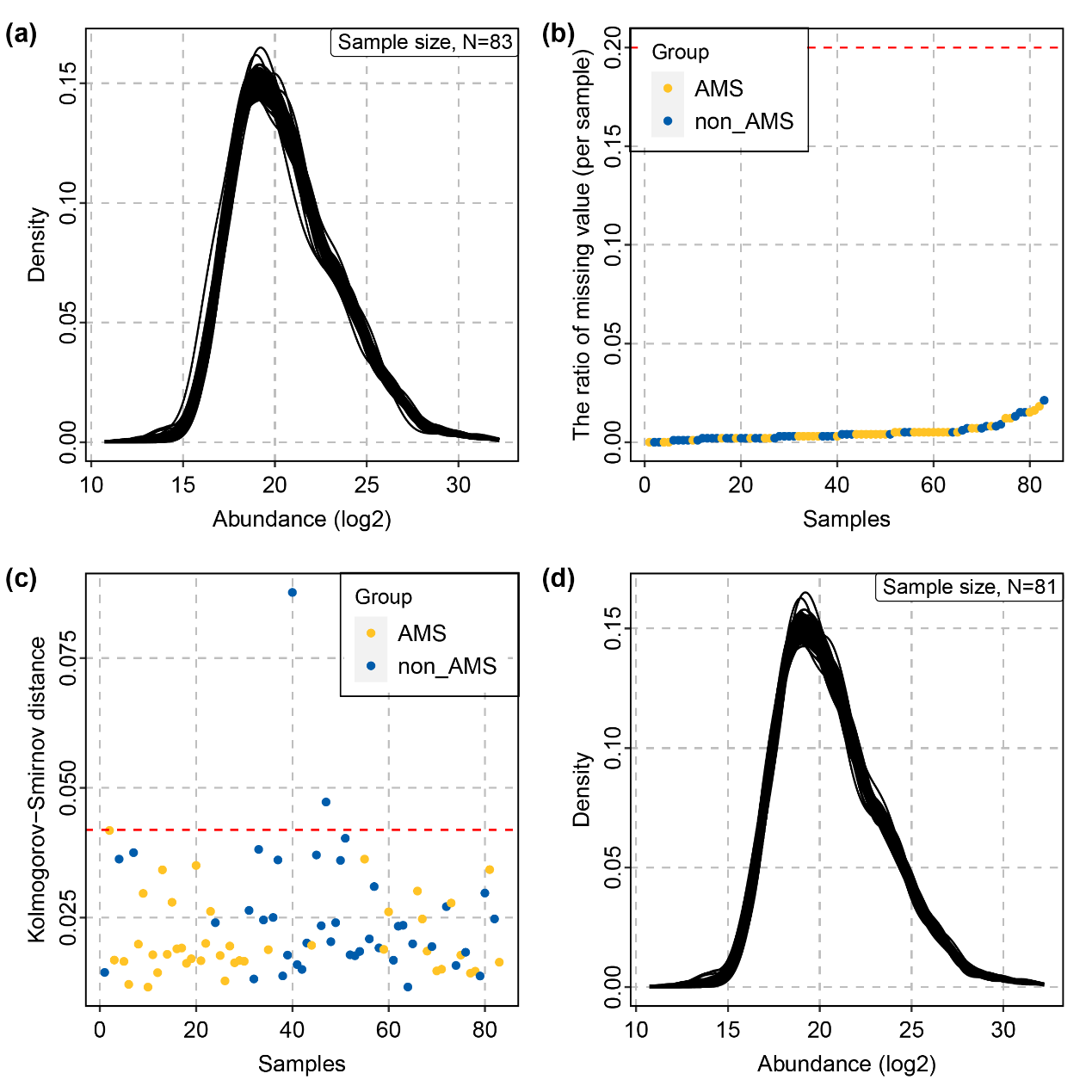


## **Fig. S2** Metabolite intensity distribution and missing value fraction in samples. **a** 989 unique metabolites were measured in 83 samples. A density distribution with Gaussian kernel was used to estimate the metabolite abundance distribution in each sample. **b** Metabolite abundance was measured in plasma from non-AMS (blue dot, n = 41) and AMS (yellow dot, n = 42) individuals. The fraction of proteins with missing value in each sample was plotted in an increasing order. **c** The distances between single sample metabolite abundance distribution and overall metabolite abundance distribution were calculated using the “Kolmogorov-Smirnov” test. The threshold of 0.042 was calculated using Q3 + 2 IQR. 2 samples above the threshold were excluded from the following analyses. **d** The metabolomic profiles from 81 samples were retained after quality control, and the density distributions were estimated using the same method as in (a).


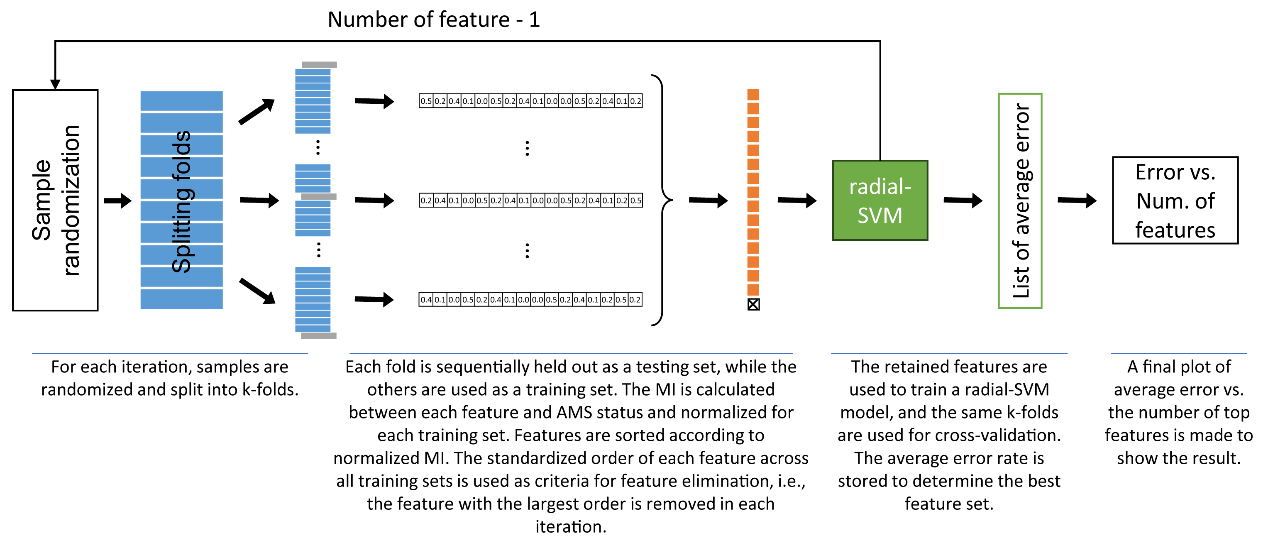


## **Fig. S3** The flowchart of the Mutual Information-radial kernel-based Support Vector Machine-Recursively Feature Elimination (MI-radialSVM-RFE) method. The molecular features from the modules significantly associated with AMS are used as the initial set of features. After applying the MI-radialSVM-RFE method, a subset of features with the lowest error rate is identified as the optimal hub of key features with the maximum predictive power.

**
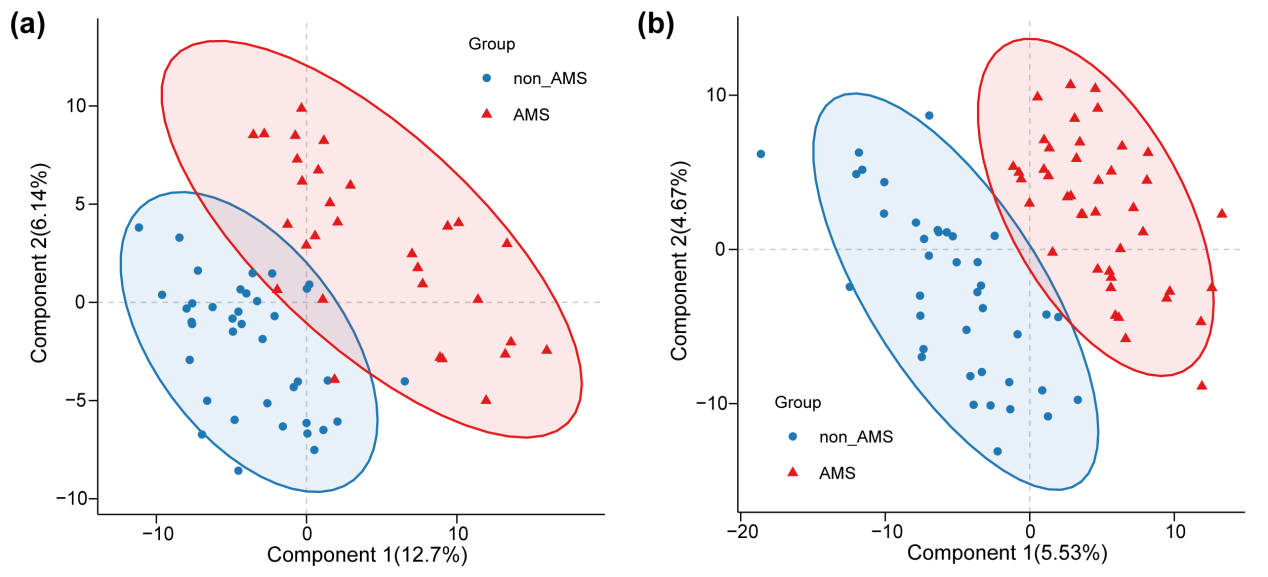
**

**Fig. S4** Pot of orthogonal partial least squares discriminant analysis (OPLS-DA). **a** OPLS-DA plot of the proteomic data. **b** OPLS-DA plot of the metabolomic data.


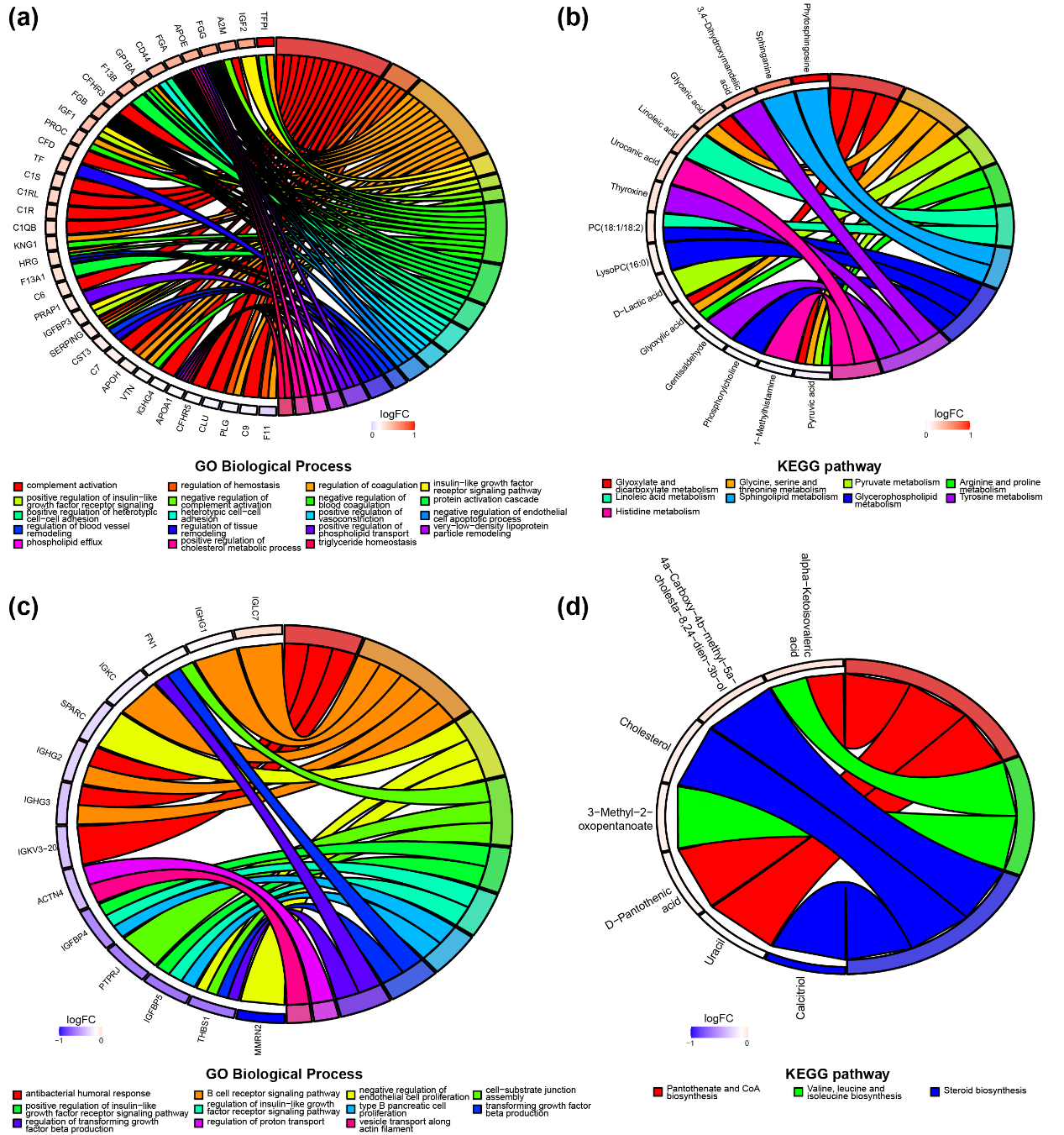


**Fig. S5** Chord plot for enriched GO biological processes (BPs) and KEGG pathways. Chord plot for enriched GO BPs (**a**) and KEGG pathways (**b**) for proteins and metabolites in protein-metabolite co-expression modules that showed up-regulation with AMS degree. Chord plot for enriched GO BPs (**c**) and KEGG pathways (**d**) for proteins and metabolites in protein-metabolite co-expression modules that showed down-regulation with AMS degree. Abbreviation: GO, gene ontology; KEGG: kyoto encyclopedia of genes and genomes

1. **Supplementary Data**

| **Molecular ID** | **GeneSymbol/Metabolite Name** | **Type** | **Module** |
| --- | --- | --- | --- |
| A0A075B6H7 | IGKV3-7 | protein | M16 |
| A0A075B6H9 | IGLV4-69 | protein | M1 |
| A0A075B6I0 | IGLV8-61 | protein | M4 |
| A0A075B6I4 | IGLV10-54 | protein | M1 |
| A0A075B6I9 | IGLV7-46 | protein | M1 |
| A0A075B6J1 | IGLV5-37 | protein | M2 |
| A0A075B6J2 | IGLV2-33 | protein | M1 |
| A0A075B6J9 | IGLV2-18 | protein | M1 |
| A0A075B6K0 | IGLV3-16 | protein | M2 |
| A0A075B6K2 | IGLV3-12 | protein | M2 |
| A0A075B6K4 | IGLV3-10 | protein | M1 |
| A0A075B6K5 | IGLV3-9 | protein | M1 |
| A0A075B6P5 | IGKV2-28 | protein | M16 |
| A0A075B6Q5 | IGHV3-64 | protein | M2 |
| A0A075B6R2 | IGHV4-4 | protein | M5 |
| A0A075B6R9 | IGKV2D-24 | protein | M1 |
| A0A075B6S2 | IGKV2D-29 | protein | M1 |
| A0A075B6S5 | IGKV1-27 | protein | M1 |
| A0A075B6S9 | IGKV1-37 | protein | M2 |
| A0A087WSX0 | IGLV5-45 | protein | M1 |
| A0A087WSY4 | IGHV4-30-2 | protein | M5 |
| A0A087WSY6 | IGKV3D-15 | protein | M16 |
| A0A087WSZ0 | IGKV1D-8 | protein | M5 |
| A0A087WW87 | IGKV2-40 | protein | M1 |
| A0A0A0MRZ9 | IGLV5-52 | protein | M2 |
| A0A0A0MS14 | IGHV1-45 | protein | M1 |
| A0A0A0MS15 | IGHV3-49 | protein | M1 |
| A0A0A0MT36 | IGKV6D-21 | protein | M1 |
| A0A0A0MT89 | IGKJ1 | protein | M2 |
| A0A0B4J1U3 | IGLV1-36 | protein | M2 |
| A0A0B4J1U7 | IGHV6-1 | protein | M1 |
| A0A0B4J1V0 | IGHV3-15 | protein | M1 |
| A0A0B4J1V1 | IGHV3-21 | protein | M4 |
| A0A0B4J1V2 | IGHV2-26 | protein | M5 |
| A0A0B4J1V6 | IGHV3-73 | protein | M16 |
| A0A0B4J1X5 | IGHV3-74 | protein | M1 |
| A0A0B4J1X8 | IGHV3-43 | protein | M1 |
| A0A0B4J1Y8 | IGLV9-49 | protein | M17 |
| A0A0B4J1Y9 | IGHV3-72 | protein | M1 |
| A0A0B4J2D9 | IGKV1D-13 | protein | M1 |
| A0A0C4DH24 | IGKV6-21 | protein | M11 |
| A0A0C4DH25 | IGKV3D-20 | protein | M1 |
| A0A0C4DH29 | IGHV1-3 | protein | M1 |
| A0A0C4DH31 | IGHV1-18 | protein | M1 |
| A0A0C4DH33 | IGHV1-24 | protein | M1 |
| A0A0C4DH34 | IGHV4-28 | protein | M1 |
| A0A0C4DH35 | IGHV3-35 | protein | M5 |
| A0A0C4DH36 | IGHV3-38 | protein | M16 |
| A0A0C4DH38 | IGHV5-51 | protein | M1 |
| A0A0C4DH43 | IGHV2-70D | protein | M2 |
| A0A0C4DH67 | IGKV1-8 | protein | M1 |
| A0A0C4DH73 | IGKV1-12 | protein | M1 |
| A0A0G2JMI3 | IGHV1-69-2 | protein | M17 |
| A0A0G2JS06 | IGLV5-39 | protein | M1 |
| A0A0J9YX35 | IGHV3-64D | protein | M1 |
| A0A0J9YXX1 | IGHV5-10-1 | protein | M16 |
| A0A2R8Y619 | H2BK1 | protein | M16 |
| A0M8Q6 | IGLC7 | protein | M5 |
| A6NNZ2 | TUBB8B | protein | M16 |
| O00187 | MASP2 | protein | M10 |
| O00231 | PSMD11 | protein | M3 |
| O00391 | QSOX1 | protein | M3 |
| O00533 | CHL1 | protein | M16 |
| O14498 | ISLR | protein | M9 |
| O14786 | NRP1 | protein | M6 |
| O14791 | APOL1 | protein | M3 |
| O43707 | ACTN4 | protein | M5 |
| O43866 | CD5L | protein | M9 |
| O60488 | ACSL4 | protein | M1 |
| O60879 | DIAPH2 | protein | M5 |
| O75144 | ICOSLG | protein | M6 |
| O75636 | FCN3 | protein | M10 |
| O75882 | ATRN | protein | M10 |
| O95445 | APOM | protein | M10 |
| O95497 | VNN1 | protein | M7 |
| P00390 | GSR | protein | M17 |
| P00450 | CP | protein | M7 |
| P00488 | F13A1 | protein | M6 |
| P00734 | F2 | protein | M10 |
| P00736 | C1R | protein | M6 |
| P00738 | HP | protein | M3 |
| P00739 | HPR | protein | M3 |
| P00740 | F9 | protein | M10 |
| P00742 | F10 | protein | M10 |
| P00746 | CFD | protein | M6 |
| P00747 | PLG | protein | M3 |
| P00748 | F12 | protein | M4 |
| P00751 | CFB | protein | M7 |
| P00915 | CA1 | protein | M10 |
| P01008 | SERPINC1 | protein | M17 |
| P01009 | SERPINA1 | protein | M10 |
| P01011 | SERPINA3 | protein | M6 |
| P01019 | AGT | protein | M3 |
| P01023 | A2M | protein | M3 |
| P01024 | C3 | protein | M10 |
| P01031 | C5 | protein | M7 |
| P01033 | TIMP1 | protein | M17 |
| P01034 | CST3 | protein | M6 |
| P01042 | KNG1 | protein | M6 |
| P01344 | IGF2 | protein | M3 |
| P01591 | JCHAIN | protein | M9 |
| P01593 | IGKV1D-33 | protein | M1 |
| P01597 | IGKV1-39 | protein | M1 |
| P01599 | IGKV1-17 | protein | M1 |
| P01601 | IGKV1D-16 | protein | M1 |
| P01602 | IGKV1-5 | protein | M1 |
| P01619 | IGKV3-20 | protein | M1 |
| P01624 | IGKV3-15 | protein | M1 |
| P01699 | IGLV1-44 | protein | M16 |
| P01700 | IGLV1-47 | protein | M1 |
| P01701 | IGLV1-51 | protein | M1 |
| P01703 | IGLV1-40 | protein | M1 |
| P01704 | IGLV2-14 | protein | M5 |
| P01705 | IGLV2-23 | protein | M2 |
| P01706 | IGLV2-11 | protein | M12 |
| P01709 | IGLV2-8 | protein | M1 |
| P01714 | IGLV3-19 | protein | M1 |
| P01715 | IGLV3-1 | protein | M1 |
| P01717 | IGLV3-25 | protein | M1 |
| P01718 | IGLV3-27 | protein | M5 |
| P01721 | IGLV6-57 | protein | M3 |
| P01742 | IGHV1-69 | protein | M1 |
| P01743 | IGHV1-46 | protein | M1 |
| P01762 | IGHV3-11 | protein | M1 |
| P01764 | IGHV3-23 | protein | M1 |
| P01766 | IGHV3-13 | protein | M1 |
| P01768 | IGHV3-30 | protein | M5 |
| P01780 | IGHV3-7 | protein | M1 |
| P01782 | IGHV3-9 | protein | M1 |
| P01817 | IGHV2-5 | protein | M1 |
| P01833 | PIGR | protein | M9 |
| P01834 | IGKC | protein | M1 |
| P01857 | IGHG1 | protein | M5 |
| P01859 | IGHG2 | protein | M1 |
| P01860 | IGHG3 | protein | M1 |
| P01861 | IGHG4 | protein | M3 |
| P01871 | IGHM | protein | M9 |
| P01876 | IGHA1 | protein | M12 |
| P01877 | IGHA2 | protein | M2 |
| P01880 | IGHD | protein | M2 |
| P02042 | HBD | protein | M10 |
| P02647 | APOA1 | protein | M16 |
| P02649 | APOE | protein | M16 |
| P02652 | APOA2 | protein | M6 |
| P02654 | APOC1 | protein | M2 |
| P02655 | APOC2 | protein | M14 |
| P02656 | APOC3 | protein | M14 |
| P02671 | FGA | protein | M6 |
| P02675 | FGB | protein | M6 |
| P02679 | FGG | protein | M6 |
| P02743 | APCS | protein | M3 |
| P02745 | C1QA | protein | M10 |
| P02746 | C1QB | protein | M6 |
| P02747 | C1QC | protein | M14 |
| P02748 | C9 | protein | M6 |
| P02749 | APOH | protein | M3 |
| P02750 | LRG1 | protein | M6 |
| P02751 | FN1 | protein | M17 |
| P02753 | RBP4 | protein | M10 |
| P02760 | AMBP | protein | M10 |
| P02763 | ORM1 | protein | M10 |
| P02765 | AHSG | protein | M6 |
| P02766 | TTR | protein | M14 |
| P02768 | ALB | protein | M6 |
| P02774 | GC | protein | M6 |
| P02775 | PPBP | protein | M17 |
| P02776 | PF4 | protein | M17 |
| P02786 | TFRC | protein | M10 |
| P02787 | TF | protein | M6 |
| P02788 | LTF | protein | M10 |
| P02790 | HPX | protein | M6 |
| P03950 | ANG | protein | M14 |
| P03951 | F11 | protein | M3 |
| P03952 | KLKB1 | protein | M10 |
| P03973 | SLPI | protein | M3 |
| P04003 | C4BPA | protein | M2 |
| P04004 | VTN | protein | M6 |
| P04040 | CAT | protein | M2 |
| P04070 | PROC | protein | M6 |
| P04075 | ALDOA | protein | M17 |
| P04114 | APOB | protein | M10 |
| P04180 | LCAT | protein | M10 |
| P04196 | HRG | protein | M6 |
| P04211 | IGLV7-43 | protein | M1 |
| P04217 | A1BG | protein | M6 |
| P04264 | KRT1 | protein | M2 |
| P04275 | VWF | protein | M16 |
| P04278 | SHBG | protein | M6 |
| P04430 | IGKV1-16 | protein | M1 |
| P04433 | IGKV3-11 | protein | M1 |
| P04439 | HLA-A | protein | M17 |
| P04908 | H2AC8 | protein | M16 |
| P05019 | IGF1 | protein | M3 |
| P05062 | ALDOB | protein | M2 |
| P05090 | APOD | protein | M16 |
| P05109 | S100A8 | protein | M7 |
| P05154 | SERPINA5 | protein | M9 |
| P05155 | SERPING1 | protein | M3 |
| P05156 | CFI | protein | M10 |
| P05160 | F13B | protein | M6 |
| P05452 | CLEC3B | protein | M3 |
| P05543 | SERPINA7 | protein | M10 |
| P05546 | SERPIND1 | protein | M3 |
| P06276 | BCHE | protein | M10 |
| P06310 | IGKV2-30 | protein | M16 |
| P06312 | IGKV4-1 | protein | M1 |
| P06331 | IGHV4-34 | protein | M1 |
| P06396 | GSN | protein | M3 |
| P06681 | C2 | protein | M10 |
| P06702 | S100A9 | protein | M7 |
| P06727 | APOA4 | protein | M12 |
| P07195 | LDHB | protein | M10 |
| P07225 | PROS1 | protein | M10 |
| P07333 | CSF1R | protein | M16 |
| P07357 | C8A | protein | M10 |
| P07358 | C8B | protein | M10 |
| P07359 | GP1BA | protein | M6 |
| P07360 | C8G | protein | M10 |
| P07602 | PSAP | protein | M16 |
| P07996 | THBS1 | protein | M17 |
| P07998 | RNASE1 | protein | M17 |
| P08185 | SERPINA6 | protein | M10 |
| P08195 | SLC3A2 | protein | M16 |
| P08253 | MMP2 | protein | M3 |
| P08294 | SOD3 | protein | M3 |
| P08493 | MGP | protein | M14 |
| P08519 | LPA | protein | M2 |
| P08571 | CD14 | protein | M6 |
| P08603 | CFH | protein | M7 |
| P08637 | FCGR3A | protein | M3 |
| P08697 | SERPINF2 | protein | M10 |
| P08709 | F7 | protein | M4 |
| P09172 | DBH | protein | M16 |
| P09486 | SPARC | protein | M17 |
| P09871 | C1S | protein | M6 |
| P0C0L4 | C4A | protein | M7 |
| P0C0L5 | C4B_2 | protein | M7 |
| P0CF74 | IGLC6 | protein | M16 |
| P0DJI8 | SAA1 | protein | M7 |
| P0DOX2 | P0DOX2 | protein | M12 |
| P0DOX3 | P0DOX3 | protein | M2 |
| P0DOX4 | P0DOX4 | protein | M16 |
| P0DOX5 | P0DOX5 | protein | M1 |
| P0DOX6 | P0DOX6 | protein | M9 |
| P0DOX7 | P0DOX7 | protein | M1 |
| P0DOX8 | P0DOX8 | protein | M1 |
| P0DOY3 | IGLC3 | protein | M16 |
| P0DP01 | IGHV1-8 | protein | M1 |
| P0DP02 | IGHV3-30-3 | protein | M1 |
| P0DTE1 | IGHV3-38-3 | protein | M1 |
| P10643 | C7 | protein | M3 |
| P10646 | TFPI | protein | M6 |
| P10720 | PF4V1 | protein | M17 |
| P10909 | CLU | protein | M3 |
| P11021 | HSPA5 | protein | M2 |
| P11226 | MBL2 | protein | M4 |
| P11279 | LAMP1 | protein | M4 |
| P11597 | CETP | protein | M10 |
| P12109 | COL6A1 | protein | M17 |
| P12111 | COL6A3 | protein | M2 |
| P12259 | F5 | protein | M10 |
| P12318 | FCGR2A | protein | M11 |
| P12830 | CDH1 | protein | M2 |
| P12955 | PEPD | protein | M7 |
| P13591 | NCAM1 | protein | M3 |
| P13598 | ICAM2 | protein | M3 |
| P13645 | KRT10 | protein | M2 |
| P13671 | C6 | protein | M3 |
| P13796 | LCP1 | protein | M6 |
| P14151 | SELL | protein | M6 |
| P14625 | HSP90B1 | protein | M6 |
| P15036 | ETS2 | protein | M6 |
| P15144 | ANPEP | protein | M14 |
| P15151 | PVR | protein | M17 |
| P15169 | CPN1 | protein | M10 |
| P15814 | IGLL1 | protein | M1 |
| P16070 | CD44 | protein | M6 |
| P17936 | IGFBP3 | protein | M3 |
| P18065 | IGFBP2 | protein | M6 |
| P18428 | LBP | protein | M6 |
| P19320 | VCAM1 | protein | M3 |
| P19652 | ORM2 | protein | M10 |
| P19823 | ITIH2 | protein | M10 |
| P19827 | ITIH1 | protein | M10 |
| P20742 | PZP | protein | M5 |
| P20851 | C4BPB | protein | M10 |
| P22105 | TNXB | protein | M2 |
| P22352 | GPX3 | protein | M10 |
| P22692 | IGFBP4 | protein | M17 |
| P22792 | CPN2 | protein | M10 |
| P22891 | PROZ | protein | M10 |
| P23083 | IGHV1-2 | protein | M1 |
| P23142 | FBLN1 | protein | M3 |
| P23470 | PTPRG | protein | M3 |
| P24592 | IGFBP6 | protein | M16 |
| P24593 | IGFBP5 | protein | M17 |
| P25311 | AZGP1 | protein | M17 |
| P26927 | MST1 | protein | M10 |
| P27169 | PON1 | protein | M6 |
| P27918 | CFP | protein | M10 |
| P29622 | SERPINA4 | protein | M10 |
| P30043 | BLVRB | protein | M10 |
| P32119 | PRDX2 | protein | M10 |
| P33151 | CDH5 | protein | M16 |
| P33908 | MAN1A1 | protein | M6 |
| P34096 | RNASE4 | protein | M7 |
| P35443 | THBS4 | protein | M13 |
| P35527 | KRT9 | protein | M2 |
| P35542 | SAA4 | protein | M6 |
| P35858 | IGFALS | protein | M10 |
| P35908 | KRT2 | protein | M2 |
| P36955 | SERPINF1 | protein | M6 |
| P36980 | CFHR2 | protein | M7 |
| P40189 | IL6ST | protein | M17 |
| P41222 | PTGDS | protein | M3 |
| P43121 | MCAM | protein | M4 |
| P43251 | BTD | protein | M10 |
| P43652 | AFM | protein | M10 |
| P47897 | QARS1 | protein | M2 |
| P48740 | MASP1 | protein | M10 |
| P49747 | COMP | protein | M13 |
| P49908 | SELENOP | protein | M3 |
| P49913 | CAMP | protein | M7 |
| P51797 | CLCN6 | protein | M1 |
| P51884 | LUM | protein | M3 |
| P54108 | CRISP3 | protein | M16 |
| P54289 | CACNA2D1 | protein | M17 |
| P55056 | APOC4 | protein | M10 |
| P55058 | PLTP | protein | M3 |
| P55103 | INHBC | protein | M10 |
| P55290 | CDH13 | protein | M3 |
| P58335 | ANTXR2 | protein | M2 |
| P59665 | DEFA1B | protein | M4 |
| P60709 | ACTB | protein | M17 |
| P61626 | LYZ | protein | M6 |
| P61769 | B2M | protein | M6 |
| P62805 | H4-16 | protein | M16 |
| P68104 | EEF1A1 | protein | M16 |
| P68871 | HBB | protein | M10 |
| P69905 | HBA2 | protein | M10 |
| P80108 | GPLD1 | protein | M10 |
| P80723 | BASP1 | protein | M17 |
| P80748 | IGLV3-21 | protein | M2 |
| P98160 | HSPG2 | protein | M16 |
| Q01459 | CTBS | protein | M10 |
| Q02985 | CFHR3 | protein | M6 |
| Q03591 | CFHR1 | protein | M3 |
| Q04756 | HGFAC | protein | M3 |
| Q06033 | ITIH3 | protein | M6 |
| Q08380 | LGALS3BP | protein | M2 |
| Q12805 | EFEMP1 | protein | M3 |
| Q12860 | CNTN1 | protein | M16 |
| Q12907 | LMAN2 | protein | M16 |
| Q12913 | PTPRJ | protein | M17 |
| Q13103 | SPP2 | protein | M17 |
| Q13201 | MMRN1 | protein | M1 |
| Q13790 | APOF | protein | M6 |
| Q13822 | ENPP2 | protein | M9 |
| Q14126 | DSG2 | protein | M3 |
| Q14515 | SPARCL1 | protein | M16 |
| Q14520 | HABP2 | protein | M10 |
| Q14624 | ITIH4 | protein | M6 |
| Q15063 | POSTN | protein | M16 |
| Q15113 | PCOLCE | protein | M9 |
| Q15166 | PON3 | protein | M3 |
| Q15485 | FCN2 | protein | M2 |
| Q15582 | TGFBI | protein | M6 |
| Q15848 | ADIPOQ | protein | M14 |
| Q16610 | ECM1 | protein | M3 |
| Q16853 | AOC3 | protein | M2 |
| Q6EMK4 | VASN | protein | M3 |
| Q6UX71 | PLXDC2 | protein | M4 |
| Q6UXB8 | PI16 | protein | M17 |
| Q6UY14 | ADAMTSL4 | protein | M3 |
| Q6YHK3 | CD109 | protein | M2 |
| Q6ZN30 | BNC2 | protein | M3 |
| Q76LX8 | ADAMTS13 | protein | M6 |
| Q86SQ4 | ADGRG6 | protein | M9 |
| Q86U17 | SERPINA11 | protein | M17 |
| Q86UD1 | OAF | protein | M3 |
| Q86UX7 | FERMT3 | protein | M1 |
| Q86VB7 | CD163 | protein | M2 |
| Q8IUL8 | CILP2 | protein | M16 |
| Q8IWU2 | LMTK2 | protein | M3 |
| Q8NBP7 | PCSK9 | protein | M12 |
| Q8TAV0 | FAM76A | protein | M2 |
| Q8TAV3 | CYP2W1 | protein | M3 |
| Q8WWA0 | ITLN1 | protein | M16 |
| Q92496 | CFHR4 | protein | M7 |
| Q92820 | GGH | protein | M3 |
| Q92945 | KHSRP | protein | M3 |
| Q92954 | PRG4 | protein | M10 |
| Q96IY4 | CPB2 | protein | M10 |
| Q96KN2 | CNDP1 | protein | M10 |
| Q96NZ9 | PRAP1 | protein | M16 |
| Q96PD5 | PGLYRP2 | protein | M6 |
| Q96S96 | PEBP4 | protein | M16 |
| Q99784 | OLFM1 | protein | M2 |
| Q99969 | RARRES2 | protein | M17 |
| Q9BUN1 | MENT | protein | M17 |
| Q9BWM5 | ZNF416 | protein | M6 |
| Q9BWP8 | COLEC11 | protein | M17 |
| Q9BXR6 | CFHR5 | protein | M3 |
| Q9C0D0 | PHACTR1 | protein | M12 |
| Q9H257 | CARD9 | protein | M2 |
| Q9H4G4 | GLIPR2 | protein | M17 |
| Q9H6T0 | ESRP2 | protein | M3 |
| Q9H8L6 | MMRN2 | protein | M17 |
| Q9HDC9 | APMAP | protein | M16 |
| Q9NP78 | ABCB9 | protein | M6 |
| Q9NP80 | PNPLA8 | protein | M2 |
| Q9NPH3 | IL1RAP | protein | M3 |
| Q9NPR2 | SEMA4B | protein | M9 |
| Q9NQ79 | CRTAC1 | protein | M16 |
| Q9NZP8 | C1RL | protein | M6 |
| Q9UBX5 | FBLN5 | protein | M6 |
| Q9UEW3 | MARCO | protein | M7 |
| Q9UGM5 | FETUB | protein | M6 |
| Q9UHG3 | PCYOX1 | protein | M10 |
| Q9UJJ9 | GNPTG | protein | M6 |
| Q9UK55 | SERPINA10 | protein | M10 |
| Q9UNN8 | PROCR | protein | M9 |
| Q9UNW1 | MINPP1 | protein | M4 |
| Q9Y2Z0 | SUGT1 | protein | M6 |
| Q9Y5Q9 | GTF3C3 | protein | M16 |
| Q9Y5Y7 | LYVE1 | protein | M3 |
| Q9Y646 | CPQ | protein | M9 |
| Q9Y6R7 | FCGBP | protein | M2 |
| Q9Y6Z7 | COLEC10 | protein | M10 |
| 1 | 4-Acetylbutyrate | metabolite | M17 |
| 2 | Nutriacholic acid | metabolite | M16 |
| 3 | 3-Hydroxydecanoic acid | metabolite | M15 |
| 4 | Gentisaldehyde | metabolite | M16 |
| 5 | L-Serine | metabolite | M11 |
| 6 | 1-Butylamine | metabolite | M11 |
| 7 | Ethenyl acetate | metabolite | M11 |
| 8 | 2-Methyl-2-buten-1-ol | metabolite | M16 |
| 9 | Trifluoromethanesulfonic acid | metabolite | M4 |
| 10 | Pyrrolidine | metabolite | M7 |
| 11 | Histamine | metabolite | M11 |
| 12 | Dimethylglycine | metabolite | M2 |
| 13 | Benzaldehyde | metabolite | M2 |
| 14 | Beta-Aminopropionitrile | metabolite | M2 |
| 15 | ACar(12:3) | metabolite | M15 |
| 16 | 9-OxoODE | metabolite | M12 |
| 17 | cis,cis-Muconic acid | metabolite | M11 |
| 18 | Glyoxylic acid | metabolite | M3 |
| 19 | Mesylate | metabolite | M5 |
| 20 | Pelargonic acid | metabolite | M5 |
| 21 | Sphingosine 1-phosphate | metabolite | M17 |
| 22 | Isobutyrylglycine | metabolite | M11 |
| 23 | D-Glutamine | metabolite | M11 |
| 24 | 4-(1,1,3,3-Tetramethylbutyl)-phenol | metabolite | M8 |
| 25 | 2,3-Butanediol | metabolite | M17 |
| 26 | Androsterone sulfate | metabolite | M3 |
| 27 | Betaine | metabolite | M17 |
| 28 | O-Acetylcarnitine | metabolite | M15 |
| 29 | indolin-2-one | metabolite | M16 |
| 30 | Methylmalonic acid | metabolite | M9 |
| 31 | DL-Tyrosine | metabolite | M2 |
| 32 | D-Proline | metabolite | M12 |
| 33 | Phosphorylcholine | metabolite | M16 |
| 34 | DL-Tryptophan | metabolite | M2 |
| 35 | Cotinine | metabolite | M5 |
| 36 | Decanoylcarnitine | metabolite | M15 |
| 37 | L-Phenylalanine | metabolite | M2 |
| 38 | L-Pipecolic acid | metabolite | M11 |
| 39 | L-Lysine | metabolite | M17 |
| 40 | Sarcosine | metabolite | M2 |
| 41 | Chenodeoxycholic acid glycine conjugate | metabolite | M12 |
| 42 | L-Leucine | metabolite | M10 |
| 43 | Eicosapentaenoic acid | metabolite | M15 |
| 44 | SM(d14:0/20:1) | metabolite | M16 |
| 45 | Safrole | metabolite | M11 |
| 46 | L-Pyroglutamic acid | metabolite | M11 |
| 47 | Indole-3-carboxaldehyde | metabolite | M2 |
| 48 | N-Methylsalsolinol | metabolite | M4 |
| 49 | L-Threonic acid | metabolite | M3 |
| 50 | 2-Pyrrolidinone | metabolite | M3 |
| 51 | LysoPA(16:0/0:0) | metabolite | M3 |
| 52 | 1,2,3-Trihydroxybenzene | metabolite | M17 |
| 53 | Dodecanoylcarnitine | metabolite | M15 |
| 54 | SM(d18:1/16:0) | metabolite | M16 |
| 55 | Biliverdin | metabolite | M6 |
| 56 | SM(d18:2/16:0) | metabolite | M16 |
| 57 | 2-Methylbutyroylcarnitine | metabolite | M7 |
| 58 | Lauroyl diethanolamide | metabolite | M11 |
| 59 | Hippuric acid | metabolite | M11 |
| 60 | SM(d14:0/22:2) | metabolite | M16 |
| 61 | Diethanolamine | metabolite | M4 |
| 62 | 5-Hydroxyhexanoic acid | metabolite | M2 |
| 63 | Perillic acid | metabolite | M7 |
| 64 | Ethylbenzene | metabolite | M5 |
| 65 | Setariol | metabolite | M2 |
| 66 | 3-Aminopropionaldehyde | metabolite | M1 |
| 67 | Acetylglycine | metabolite | M2 |
| 68 | Propionylcarnitine | metabolite | M16 |
| 69 | Theophylline | metabolite | M4 |
| 70 | L-Arginine | metabolite | M5 |
| 71 | 5-Methoxy-2-methylthiazole | metabolite | M4 |
| 72 | N4-Acetylaminobutanal | metabolite | M4 |
| 73 | (±)-Jasmonic acid | metabolite | M7 |
| 74 | 4-Hydroxybenzaldehyde | metabolite | M2 |
| 75 | Prostaglandin D2 | metabolite | M15 |
| 76 | alpha-Tocopherol | metabolite | M16 |
| 77 | L-(-)-Fucose | metabolite | M9 |
| 78 | Docosahexaenoic acid | metabolite | M15 |
| 79 | 7-Ethoxy-4-methyl-2H-1-benzopyran-2-one | metabolite | M11 |
| 80 | Arecaidine | metabolite | M12 |
| 81 | Chenodeoxycholic acid | metabolite | M12 |
| 82 | D-Aspartic acid | metabolite | M4 |
| 83 | Indoxyl sulfate | metabolite | M16 |
| 84 | SM(d14:0/22:1) | metabolite | M4 |
| 85 | N-Ethylacetamide | metabolite | M2 |
| 86 | Piperidine | metabolite | M10 |
| 87 | L-Carnitine | metabolite | M2 |
| 88 | N6-Methyladenosine | metabolite | M17 |
| 89 | ACar(11:0) | metabolite | M7 |
| 90 | Alpha-dimorphecolic acid | metabolite | M8 |
| 91 | Glyceraldehyde | metabolite | M17 |
| 92 | FAHFA(18:2/22:3) | metabolite | M15 |
| 93 | ACar(16:1) | metabolite | M15 |
| 94 | FAHFA(16:1/22:3) | metabolite | M15 |
| 95 | FAHFA(18:1/18:0) | metabolite | M15 |
| 96 | Pseudouridine | metabolite | M17 |
| 97 | (R)-lipoic acid | metabolite | M11 |
| 98 | FAHFA(2:0/22:3) | metabolite | M5 |
| 99 | ACar(10:1) | metabolite | M15 |
| 100 | 3,4-Dihydro-2H-1-benzopyran-2-one | metabolite | M2 |
| 101 | PG(18:1/18:1) | metabolite | M16 |
| 102 | 4-Hydroxy-2-butenoic acid gamma-lactone | metabolite | M11 |
| 103 | FAHFA(18:1/22:3) | metabolite | M16 |
| 104 | o-Methoxyphenyl sulfate | metabolite | M4 |
| 105 | L-Histidine | metabolite | M2 |
| 106 | ACar(7:0) | metabolite | M5 |
| 107 | Glycolic acid | metabolite | M4 |
| 108 | Metenamine | metabolite | M16 |
| 109 | ACar(14:0) | metabolite | M15 |
| 110 | ACar(8:1) | metabolite | M15 |
| 111 | Palmitic amide | metabolite | M5 |
| 112 | Cholesterol | metabolite | M5 |
| 113 | 3-Methylxanthine | metabolite | M16 |
| 114 | FAHFA(16:0/22:3) | metabolite | M15 |
| 115 | Trimethylamine N-oxide | metabolite | M4 |
| 116 | Indole-3-acetic acid | metabolite | M16 |
| 117 | Taurine | metabolite | M17 |
| 118 | ACar(8:0) | metabolite | M15 |
| 119 | ACar(6:0) | metabolite | M15 |
| 120 | L-Hydroxyproline | metabolite | M17 |
| 121 | 1-Pyrrolidinecarboxaldehyde | metabolite | M2 |
| 122 | Butyrylcarnitine | metabolite | M2 |
| 123 | Erythrulose | metabolite | M16 |
| 124 | LPE(20:3) | metabolite | M14 |
| 125 | N-Ethylglycine | metabolite | M5 |
| 126 | ACar(14:2) | metabolite | M15 |
| 127 | Uridine | metabolite | M17 |
| 128 | PC(18:0/20:4) | metabolite | M2 |
| 129 | PI(16:0/16:0) | metabolite | M13 |
| 130 | PC(16:2/24:4) | metabolite | M16 |
| 131 | L-Urobilin | metabolite | M4 |
| 132 | PC(16:0/20:4) | metabolite | M11 |
| 133 | 1-Methyl-L-Histidine | metabolite | M12 |
| 134 | PC(16:3/24:4) | metabolite | M16 |
| 135 | L-Kynurenine | metabolite | M17 |
| 136 | (R)-3-Hydroxy-tetradecanoic acid | metabolite | M1 |
| 137 | Gingerol | metabolite | M9 |
| 138 | FAHFA(16:0/18:2) | metabolite | M2 |
| 139 | Thyroxine | metabolite | M16 |
| 140 | LPE(16:1) | metabolite | M14 |
| 141 | LPE(18:0) | metabolite | M14 |
| 142 | 2-Benzofurancarboxaldehyde | metabolite | M2 |
| 143 | ACar(12:2) | metabolite | M15 |
| 144 | LPC(18:2) | metabolite | M13 |
| 145 | Bilirubin | metabolite | M17 |
| 146 | PE(16:0/18:2(9Z,12Z)) | metabolite | M13 |
| 147 | Malonic acid | metabolite | M3 |
| 148 | 12-Ketodeoxycholic acid | metabolite | M8 |
| 149 | Linoleic acid | metabolite | M16 |
| 150 | LPE(18:2) | metabolite | M13 |
| 151 | LPG(18:1) | metabolite | M14 |
| 152 | xi-2,3-Dihydro-3,5-dihydroxy-6-methyl-4H-pyran-4-one | metabolite | M11 |
| 153 | Dihydrothymine | metabolite | M2 |
| 154 | PC(14:1/26:4) | metabolite | M17 |
| 155 | Hexylamine | metabolite | M3 |
| 157 | 1-Pyrroline | metabolite | M11 |
| 158 | PE(16:1e/20:4) | metabolite | M12 |
| 159 | PC(8:0/26:2) | metabolite | M3 |
| 160 | LPC(20:0) | metabolite | M2 |
| 161 | PE(16:1e/18:2) | metabolite | M2 |
| 162 | Hydroxytyrosol | metabolite | M16 |
| 163 | ACar(16:2) | metabolite | M15 |
| 164 | PC(10:0/26:4) | metabolite | M12 |
| 165 | Citric acid | metabolite | M4 |
| 166 | 8,15-DiHETE | metabolite | M15 |
| 167 | PC(22:5(7Z,10Z,13Z,16Z,19Z)/18:1(11Z)) | metabolite | M17 |
| 168 | PI(18:1(9Z)/18:1(9Z)) | metabolite | M13 |
| 169 | LPE(17:0) | metabolite | M14 |
| 170 | Azelaic acid | metabolite | M16 |
| 171 | Deoxycholic acid | metabolite | M16 |
| 172 | Spathulenol | metabolite | M5 |
| 173 | N-Acetyl-L-aspartic acid | metabolite | M4 |
| 174 | ACar(18:2) | metabolite | M15 |
| 175 | PC(16:1e/18:2) | metabolite | M16 |
| 176 | Glycohyocholic acid | metabolite | M12 |
| 177 | ACar(13:1) | metabolite | M7 |
| 178 | LPC(18:0) | metabolite | M13 |
| 179 | LysoPE(16:0/0:0) | metabolite | M14 |
| 180 | N-Desmethylvenlafaxine | metabolite | M5 |
| 181 | Norvaline | metabolite | M2 |
| 182 | PC(14:0/22:3) | metabolite | M16 |
| 183 | LPE(22:4) | metabolite | M14 |
| 184 | ACar(16:3) | metabolite | M15 |
| 185 | ACar(10:2) | metabolite | M15 |
| 186 | LPE(18:3) | metabolite | M13 |
| 187 | Uric acid | metabolite | M17 |
| 188 | Dibutyl phthalate | metabolite | M3 |
| 189 | L-Threonine | metabolite | M12 |
| 190 | PC(18:0/18:2) | metabolite | M11 |
| 191 | PC(9:0/26:2) | metabolite | M16 |
| 192 | SM(d14:0/16:1) | metabolite | M5 |
| 193 | PC(18:1e/22:6) | metabolite | M2 |
| 194 | SM(d14:0/18:2) | metabolite | M5 |
| 195 | LPC(22:4) | metabolite | M14 |
| 196 | LPC(20:2) | metabolite | M14 |
| 197 | SM(d14:0/20:2) | metabolite | M2 |
| 198 | LPC(18:1) | metabolite | M13 |
| 199 | D-Mannose | metabolite | M2 |
| 200 | PC(6:0/26:1) | metabolite | M4 |
| 201 | PC(10:0/26:2) | metabolite | M11 |
| 202 | PC(12:0/22:3) | metabolite | M16 |
| 203 | PC(14:0e/19:2) | metabolite | M16 |
| 204 | PC(16:0/18:1) | metabolite | M16 |
| 205 | PC(16:0/18:2) | metabolite | M10 |
| 206 | PC(14:0/22:5) | metabolite | M16 |
| 207 | Linoelaidic acid | metabolite | M8 |
| 208 | Mesaconic acid | metabolite | M4 |
| 209 | LPE(20:2) | metabolite | M13 |
| 210 | Irisolidone | metabolite | M16 |
| 211 | PC(22:6(4Z,7Z,10Z,13Z,16Z,19Z)/18:1(11Z)) | metabolite | M16 |
| 212 | PC(14:1/24:4) | metabolite | M4 |
| 213 | PC(16:0/22:6) | metabolite | M16 |
| 214 | PC(14:0e/18:1) | metabolite | M4 |
| 215 | PC(18:1/18:2) | metabolite | M3 |
| 216 | 2-O-(5,8,11,14,17-Eicosapentaenoyl)-1-O-hexadecylglycero-3-phosphocholine | metabolite | M3 |
| 217 | SM(d14:0/19:1) | metabolite | M16 |
| 218 | PC(12:0/26:4) | metabolite | M16 |
| 219 | ACar(18:3) | metabolite | M15 |
| 220 | PC(14:1e/15:1) | metabolite | M5 |
| 221 | PE(18:0/20:4) | metabolite | M2 |
| 222 | PC(16:0e/22:6) | metabolite | M2 |
| 223 | PC(14:0e/20:3) | metabolite | M16 |
| 224 | PI(20:2(11Z,14Z)/16:0) | metabolite | M13 |
| 225 | PC(6:0/26:2) | metabolite | M16 |
| 226 | SM(d14:0/18:1) | metabolite | M5 |
| 227 | 5Z-Dodecenoic acid | metabolite | M8 |
| 228 | PC(14:0e/17:2) | metabolite | M2 |
| 229 | Alpha-N-Phenylacetyl-L-glutamine | metabolite | M16 |
| 230 | Glutaral | metabolite | M17 |
| 231 | Acetylhydrazine | metabolite | M3 |
| 232 | PC(14:0e/20:1) | metabolite | M16 |
| 233 | PC(14:0e/22:4) | metabolite | M16 |
| 234 | Lactosylceramide (d18:1/16:0) | metabolite | M2 |
| 235 | 4-Dodecylbenzenesulfonic Acid | metabolite | M11 |
| 236 | L-Malic acid | metabolite | M9 |
| 237 | 2,5-Dihydro-2,4-dimethyloxazole | metabolite | M12 |
| 238 | L-Valine | metabolite | M2 |
| 239 | Isonicotinic acid | metabolite | M10 |
| 240 | LPE(20:5) | metabolite | M13 |
| 241 | Diacetone alcohol | metabolite | M11 |
| 242 | Lutein | metabolite | M7 |
| 243 | FAHFA(18:3/18:2) | metabolite | M15 |
| 244 | 2-trans-6-cis-Dodecadienal | metabolite | M8 |
| 245 | LPC(22:6) | metabolite | M14 |
| 246 | PC(18:0/22:6) | metabolite | M17 |
| 247 | PC(14:0e/24:4) | metabolite | M16 |
| 248 | D-Lactic acid | metabolite | M3 |
| 249 | Morpholine | metabolite | M4 |
| 250 | LPC(16:0) | metabolite | M2 |
| 251 | ACar(14:3) | metabolite | M15 |
| 252 | LPC(17:1) | metabolite | M14 |
| 253 | Glyceric acid | metabolite | M3 |
| 254 | 3-(4-Hydroxyphenyl)-1-propanol | metabolite | M5 |
| 255 | (2R*,3R*)-1,2,3-Butanetriol | metabolite | M1 |
| 256 | 2-Piperidone | metabolite | M12 |
| 257 | (S)-Abscisic acid | metabolite | M7 |
| 258 | 1,3-Diisopropylbenzene | metabolite | M17 |
| 259 | PC(18:1e/20:4) | metabolite | M16 |
| 260 | PC(16:2e/24:4) | metabolite | M16 |
| 261 | ACar(9:0) | metabolite | M7 |
| 262 | MethylIndole-3-acetate | metabolite | M11 |
| 263 | Epsilon-caprolactam | metabolite | M4 |
| 264 | PC(14:1e/24:4) | metabolite | M16 |
| 265 | 4-Pyridoxic acid | metabolite | M17 |
| 266 | gamma-Glutamylleucine | metabolite | M10 |
| 267 | N-p-Coumaroyloctopamine | metabolite | M11 |
| 268 | L-Galactose | metabolite | M17 |
| 269 | Acetaminophen | metabolite | M9 |
| 270 | SM(d18:1/14:0) | metabolite | M17 |
| 271 | LPA(20:4) | metabolite | M17 |
| 272 | LPC(14:0) | metabolite | M14 |
| 273 | PC(14:0e/20:2) | metabolite | M16 |
| 274 | PC(2:0/19:0) | metabolite | M10 |
| 275 | PC(14:0e/22:3) | metabolite | M2 |
| 276 | PC(14:1/24:2) | metabolite | M4 |
| 277 | Curcumadiol | metabolite | M5 |
| 278 | LPE(20:4) | metabolite | M14 |
| 279 | 2-Methyltetrahydrofuran-3-one | metabolite | M16 |
| 280 | (E)-8-(3,6-Dimethyl-2-heptenyl)-4',5,7-trihydroxyflavanone | metabolite | M6 |
| 281 | PC(18:1/22:6) | metabolite | M13 |
| 282 | LysoPE(0:0/20:3(11Z,14Z,17Z)) | metabolite | M14 |
| 283 | Hypoxanthine | metabolite | M17 |
| 284 | PC(16:1/22:6) | metabolite | M16 |
| 285 | LPC(20:3) | metabolite | M14 |
| 286 | LPE(22:5) | metabolite | M14 |
| 287 | PC(22:2(13Z,16Z)/14:1(9Z)) | metabolite | M3 |
| 288 | PC(8:0/26:1) | metabolite | M3 |
| 289 | ACar(16:4) | metabolite | M15 |
| 290 | 4-Hydroxycinnamic acid | metabolite | M2 |
| 291 | N-Palmitoylsphingosine | metabolite | M5 |
| 292 | LPC(20:1) | metabolite | M13 |
| 293 | PC(22:2(13Z,16Z)/14:0) | metabolite | M11 |
| 294 | 3-Methoxybenzenepropanoic acid | metabolite | M3 |
| 295 | Methyl (2E,4Z)-decadienoate | metabolite | M11 |
| 296 | D-Glucuronolactone | metabolite | M4 |
| 297 | Tetradecanedioic acid | metabolite | M12 |
| 298 | Adipic acid | metabolite | M11 |
| 299 | PG(18:1/20:4) | metabolite | M16 |
| 300 | 4-Nitrophenol | metabolite | M4 |
| 301 | LPG(18:2) | metabolite | M14 |
| 302 | PC(5:0/27:0) | metabolite | M16 |
| 303 | Syringol | metabolite | M11 |
| 304 | LysoPC(O-18:0) | metabolite | M13 |
| 305 | LPC(19:1) | metabolite | M13 |
| 306 | Paradol | metabolite | M11 |
| 307 | PC(20:3(5Z,8Z,11Z)/P-18:0) | metabolite | M2 |
| 308 | 2-acetyl-1-alkyl-sn-glycero-3-phosphocholine | metabolite | M13 |
| 309 | LysoPC(17:0) | metabolite | M14 |
| 310 | 4-Isopropylbenzoic acid | metabolite | M7 |
| 311 | 5-Aminovaleric acid | metabolite | M2 |
| 312 | Methyl 2-furoate | metabolite | M11 |
| 313 | PC(16:0e/20:4) | metabolite | M16 |
| 314 | FAHFA(22:6/22:5) | metabolite | M15 |
| 315 | PC(18:1/20:4) | metabolite | M16 |
| 316 | PC(10:0/26:1) | metabolite | M11 |
| 317 | ACar(18:0) | metabolite | M15 |
| 318 | PC(16:0/16:0) | metabolite | M12 |
| 319 | Ethylphosphate | metabolite | M16 |
| 320 | PC(16:1(9Z)/P-18:1(11Z)) | metabolite | M16 |
| 321 | Polyoxyethylene (600) monoricinoleate | metabolite | M14 |
| 322 | LPC(16:1) | metabolite | M14 |
| 323 | LysoPA(0:0/18:2(9Z,12Z)) | metabolite | M13 |
| 324 | Tiglylglycine | metabolite | M11 |
| 325 | 16-Hydroxy hexadecanoic acid | metabolite | M15 |
| 326 | 1,2,3,4-Tetrahydro-1-[1-hydroxy-3-(4-hydroxyphenyl)-2-propenyl]-7-methoxy-2,6-naphthalenediol | metabolite | M11 |
| 327 | Nor-Desoxycholic acid | metabolite | M11 |
| 328 | L-Rhamnose | metabolite | M9 |
| 329 | p-Cresol sulfate | metabolite | M16 |
| 330 | Pseudooxynicotine | metabolite | M3 |
| 331 | LysoPC(16:1(9Z)/0:0) | metabolite | M14 |
| 332 | 5-Aminopentanal | metabolite | M9 |
| 333 | 5,7alpha-Dihydro-1,4,4,7a-tetramethyl-4H-indene | metabolite | M11 |
| 334 | PC(18:5e/15:1) | metabolite | M9 |
| 335 | 2-Ketobutyric acid | metabolite | M11 |
| 336 | PE(16:0/18:2) | metabolite | M16 |
| 337 | Choline | metabolite | M17 |
| 338 | PC(22:4(7Z,10Z,13Z,16Z)/16:0) | metabolite | M12 |
| 339 | 2,5-Diisopropylphenol | metabolite | M3 |
| 340 | LPA(18:2) | metabolite | M13 |
| 341 | PC(14:0e/18:2) | metabolite | M16 |
| 342 | xi-Tetrahydro-6-propyl-2H-pyran-2-one | metabolite | M2 |
| 343 | LPE(16:0) | metabolite | M14 |
| 344 | L-Ornithine | metabolite | M17 |
| 345 | Gallic acid | metabolite | M9 |
| 346 | LPE(22:6) | metabolite | M14 |
| 347 | L-homoserine | metabolite | M12 |
| 348 | FAHFA(2:0/21:0) | metabolite | M3 |
| 349 | L-Dopa | metabolite | M12 |
| 350 | 4-Hydroxymandelonitrile | metabolite | M2 |
| 351 | PC(20:4/22:6) | metabolite | M13 |
| 352 | O-Methylcorypalline | metabolite | M11 |
| 353 | Linoleamide | metabolite | M5 |
| 354 | PE(16:0/20:4) | metabolite | M16 |
| 355 | HexCer/NS(d17:1/17:0) | metabolite | M3 |
| 356 | L-Erythrulose | metabolite | M11 |
| 357 | 3-Methyl-2-oxopentanoate | metabolite | M17 |
| 358 | LPC(15:0) | metabolite | M14 |
| 359 | PE(18:2e/20:4) | metabolite | M4 |
| 360 | LPE(18:1) | metabolite | M13 |
| 361 | Enrofloxacin | metabolite | M11 |
| 362 | PC(18:2(9Z,12Z)/14:0) | metabolite | M16 |
| 363 | ACar(18:1) | metabolite | M15 |
| 364 | 2-(3-Phenylpropyl)tetrahydrofuran | metabolite | M5 |
| 365 | H-Abu-OH | metabolite | M11 |
| 366 | 1-Cyano-2-hydroxy-3-butene | metabolite | M5 |
| 367 | 2,5-Diisopropyl-4-methylphenol | metabolite | M5 |
| 368 | Methyl 3-methylbutanoate | metabolite | M11 |
| 369 | ACar(15:0) | metabolite | M7 |
| 370 | Glycine | metabolite | M11 |
| 371 | Isovaleric acid | metabolite | M5 |
| 372 | 4-(2,6,6-Trimethyl-1,3-cyclohexadien-1-yl)-2-butanone | metabolite | M11 |
| 373 | (S)-beta-Aminoisobutyric acid | metabolite | M9 |
| 374 | ACar(20:2) | metabolite | M15 |
| 375 | LysoPC(20:3(5Z,8Z,11Z)) | metabolite | M14 |
| 376 | (E,E)-2,4-Decadienal | metabolite | M11 |
| 377 | LPA(22:6) | metabolite | M14 |
| 378 | LPC(20:4) | metabolite | M14 |
| 379 | (+/-)-Dihydromintlactone | metabolite | M17 |
| 380 | Nicotinamide | metabolite | M2 |
| 381 | 20-Hydroxyeicosatetraenoic acid | metabolite | M12 |
| 382 | LysoPC(22:5(4Z,7Z,10Z,13Z,16Z)) | metabolite | M14 |
| 383 | Crustecdysone | metabolite | M9 |
| 384 | alpha-Linolenic acid | metabolite | M15 |
| 385 | 3-O-Acetylepisamarcandin | metabolite | M6 |
| 386 | D-Mannosamine | metabolite | M11 |
| 387 | 5,6-DHET | metabolite | M12 |
| 388 | LysoPC(18:1(9Z)) | metabolite | M13 |
| 389 | FAHFA(19:2/15:1) | metabolite | M2 |
| 390 | Propyl 2,4-decadienoate | metabolite | M11 |
| 391 | ACar(20:3) | metabolite | M8 |
| 392 | Tropic acid | metabolite | M16 |
| 393 | LPC(22:5) | metabolite | M14 |
| 394 | LysoPC(16:0) | metabolite | M16 |
| 395 | Trigonelline | metabolite | M12 |
| 396 | LysoPC(22:2(13Z,16Z)) | metabolite | M13 |
| 397 | LPC(18:3) | metabolite | M13 |
| 398 | 8-Heptadecenal | metabolite | M8 |
| 399 | PE(18:1/18:2) | metabolite | M16 |
| 400 | LysoPC(14:0/0:0) | metabolite | M14 |
| 401 | 8-iso-13,14-dihydro-15-keto-PGF2a | metabolite | M12 |
| 402 | ACar(6:1) | metabolite | M15 |
| 403 | 3-Indoleacrylic acid | metabolite | M2 |
| 404 | 3beta,6beta-Dihydroxynortropane | metabolite | M2 |
| 405 | LysoPC(15:0) | metabolite | M14 |
| 406 | 5,7-Dihydro-2-methylthieno[3,4-d]pyrimidine | metabolite | M11 |
| 407 | Isohumbertiol | metabolite | M16 |
| 408 | LysoPE(16:1(9Z)/0:0) | metabolite | M14 |
| 409 | Proline betaine | metabolite | M17 |
| 410 | Cinnamyl alcohol | metabolite | M5 |
| 411 | LysoPC(P-18:1(9Z)) | metabolite | M13 |
| 412 | Pentyl formate | metabolite | M5 |
| 413 | Monoethylhexyl phthalic acid | metabolite | M5 |
| 414 | D(+)-Glucose | metabolite | M11 |
| 415 | 4-Aminoantipyrine | metabolite | M11 |
| 416 | PC(16:0/16:1) | metabolite | M16 |
| 417 | 12-Leukotriene B4 | metabolite | M12 |
| 418 | Dimethyl dialkyl ammonium chloride | metabolite | M11 |
| 419 | (R)-3-Hydroxybutyric acid | metabolite | M15 |
| 420 | Caffeine | metabolite | M4 |
| 421 | 3-Methyl-1-butylamine | metabolite | M3 |
| 422 | Diethyl phthalic acid | metabolite | M11 |
| 423 | Austalide H | metabolite | M17 |
| 424 | 3,4-Dihydro-2,2,5,7,8-pentamethyl-2H-1-benzopyran-6-ol | metabolite | M8 |
| 425 | Uric acid | metabolite | M17 |
| 426 | 1-Methyl-1,3-cyclohexadiene | metabolite | M5 |
| 427 | PC(22:6(4Z,7Z,10Z,13Z,16Z,19Z)/18:3(6Z,9Z,12Z)) | metabolite | M17 |
| 428 | Reticuline | metabolite | M14 |
| 429 | N-Acetyl-L-methionine | metabolite | M17 |
| 430 | DL-Dopa | metabolite | M2 |
| 431 | LPA(18:1) | metabolite | M13 |
| 432 | PC(22:2(13Z,16Z)/16:1(9Z)) | metabolite | M4 |
| 433 | ACar(22:5) | metabolite | M14 |
| 434 | Alanylglycine | metabolite | M4 |
| 435 | 2-O-(4,7,10,13,16,19-Docosahexaenoyl)-1-O-hexadecylglycero-3-phosphocholine | metabolite | M17 |
| 436 | ACar(13:0) | metabolite | M7 |
| 437 | ACar(20:5) | metabolite | M15 |
| 438 | Prolylhydroxyproline | metabolite | M16 |
| 439 | LysoPC(22:0) | metabolite | M5 |
| 440 | Cellulose triacetate | metabolite | M11 |
| 441 | OxPC(16:0/18:1+3O) | metabolite | M16 |
| 442 | Gamma-Linolenic acid | metabolite | M15 |
| 443 | D-Xylose | metabolite | M11 |
| 444 | PI(20:2(11Z,14Z)/18:2(9Z,12Z)) | metabolite | M13 |
| 445 | PC(20:1(11Z)/14:0) | metabolite | M11 |
| 446 | 24,25-Dihydroxyvitamin D | metabolite | M5 |
| 447 | 5-Hexyltetrahydro-2-furanoctanoic acid | metabolite | M8 |
| 448 | 2-Methyl-1,3-cyclohexadiene | metabolite | M5 |
| 449 | Furanofukinin | metabolite | M12 |
| 450 | Rishitin | metabolite | M4 |
| 451 | Cysteinyl-Cysteine | metabolite | M7 |
| 452 | PC(14:1e/3:0) | metabolite | M14 |
| 453 | 2-Azetidinecarboxylic acid | metabolite | M3 |
| 454 | Catechin | metabolite | M4 |
| 455 | 2',4',6'-Trihydroxyacetophenone | metabolite | M16 |
| 456 | stearoyl sphingomyelin | metabolite | M16 |
| 457 | Butylamine | metabolite | M4 |
| 458 | 2,6-Dimethoxyphenol | metabolite | M12 |
| 459 | 1-(3-Aminopropyl)-4-aminobutanal | metabolite | M11 |
| 460 | H-PHE-PRO-OH | metabolite | M17 |
| 461 | Capsaicin | metabolite | M11 |
| 462 | Oleoyl glycine | metabolite | M15 |
| 463 | Bergapten | metabolite | M11 |
| 464 | Symmetric dimethylarginine | metabolite | M17 |
| 465 | Oleamide | metabolite | M5 |
| 466 | Isoquinoline | metabolite | M2 |
| 467 | (3beta,5alpha,6beta,22E,24R)-23-Methylergosta-7,22-diene-3,5,6-triol | metabolite | M17 |
| 468 | 6,10,14-Trimethyl-5,9,13-pentadecatrien-2-one | metabolite | M8 |
| 469 | PEtOH(19:0/18:2) | metabolite | M13 |
| 470 | FAHFA(2:0/22:5) | metabolite | M8 |
| 471 | LysoPC(18:1(11Z)) | metabolite | M13 |
| 472 | 2-Thiophenethiol | metabolite | M2 |
| 473 | (9S,10E,12Z,15Z)-9-Hydroxy-10,12,15-octadecatrienoic acid | metabolite | M12 |
| 474 | (9Z,11R,12S,13S,15Z)-12,13-Epoxy-11-hydroxy-9,15-octadecadienoic acid | metabolite | M7 |
| 475 | D8'-Merulinic acid A | metabolite | M17 |
| 476 | 16-Methylheptadecanoic acid | metabolite | M16 |
| 477 | PE(18:2e/18:2) | metabolite | M9 |
| 478 | PC(18:2/22:6) | metabolite | M13 |
| 479 | (10E,12Z)-(9S)-9-Hydroperoxyoctadeca-10,12-dienoic acid | metabolite | M7 |
| 480 | (-)-Epiafzelechin | metabolite | M12 |
| 481 | 3,5-Dihydroxybenzoic acid | metabolite | M6 |
| 482 | D-Citrulline | metabolite | M4 |
| 483 | 24-Epibrassinolide | metabolite | M8 |
| 484 | N-Acetylglycine | metabolite | M5 |
| 485 | Cer/AP(t14:1/13:1) | metabolite | M8 |
| 486 | (8E,15E)-1,8,15-Heptadecatriene-11,13-diyne | metabolite | M5 |
| 487 | LPC(16:2) | metabolite | M14 |
| 488 | LysoPE(0:0/18:0) | metabolite | M14 |
| 489 | PG(16:0/18:1) | metabolite | M14 |
| 490 | FAHFA(18:0/3:0) | metabolite | M12 |
| 491 | LysoPC(20:4(8Z,11Z,14Z,17Z)) | metabolite | M14 |
| 492 | 4-Methylcatechol | metabolite | M16 |
| 493 | PA(16:0/18:2) | metabolite | M12 |
| 494 | Maleamic acid | metabolite | M9 |
| 495 | L-Malic acid | metabolite | M17 |
| 496 | Germacr-1(10)-ene-5,8-dione | metabolite | M5 |
| 497 | Oxypurinol | metabolite | M17 |
| 498 | 3-Methyl-alpha-ionyl acetate | metabolite | M8 |
| 499 | LPC(15:1) | metabolite | M13 |
| 500 | Benzoic acid | metabolite | M16 |
| 501 | Myristic acid | metabolite | M15 |
| 502 | (9R,10S,12Z)-9,10-Dihydroxy-8-oxo-12-octadecenoic acid | metabolite | M7 |
| 503 | FAHFA(19:0/8:0) | metabolite | M3 |
| 504 | Glycyrrhetinic acid | metabolite | M11 |
| 505 | (-)-3-Isothujone | metabolite | M17 |
| 506 | ACar(14:1) | metabolite | M15 |
| 507 | Prostaglandin B2 | metabolite | M12 |
| 508 | 3,4,5-Trimethoxycinnamic acid | metabolite | M11 |
| 509 | Armillarivin | metabolite | M12 |
| 510 | Vanillin | metabolite | M16 |
| 511 | 5-Hydroxyindoleacetate | metabolite | M17 |
| 512 | Cytidine | metabolite | M16 |
| 513 | xi-2,5-Dihydro-2,4-dimethylthiazole | metabolite | M4 |
| 514 | Dodecanoic acid | metabolite | M15 |
| 515 | (3R,6'Z)-3,4-Dihydro-8-hydroxy-3-(6-pentadecenyl)-1H-2-benzopyran-1-one | metabolite | M12 |
| 516 | D-Pantothenic acid | metabolite | M17 |
| 517 | 2-Hydroxyadipic acid | metabolite | M11 |
| 518 | 2-Phenylacetamide | metabolite | M2 |
| 519 | ACar(18:4) | metabolite | M15 |
| 520 | PC(22:1(13Z)/14:0) | metabolite | M11 |
| 521 | N'-Hydroxymethylnorcotinine | metabolite | M5 |
| 522 | PG(18:2/18:2) | metabolite | M16 |
| 523 | 3,4-Dihydroxybenzoic acid | metabolite | M3 |
| 524 | ACar(16:0) | metabolite | M15 |
| 525 | Peroxysimulenoline | metabolite | M11 |
| 526 | (2R,3R,4R)-2-Amino-4-hydroxy-3-methylpentanoic acid | metabolite | M2 |
| 527 | LysoPE(20:3(11Z,14Z,17Z)/0:0) | metabolite | M14 |
| 528 | 13-L-Hydroperoxylinoleic acid | metabolite | M12 |
| 529 | Acetamidopropanal | metabolite | M5 |
| 530 | Canavanine | metabolite | M5 |
| 531 | 1-Phenyl-1,3-heptadecanedione | metabolite | M15 |
| 532 | Urocanic acid | metabolite | M16 |
| 533 | Alpha-CEHC | metabolite | M11 |
| 534 | FAHFA(11:0/16:1) | metabolite | M3 |
| 535 | LysoPC(18:4(6Z,9Z,12Z,15Z)) | metabolite | M14 |
| 536 | Hydrocotarnine | metabolite | M11 |
| 537 | Diphenylamine | metabolite | M2 |
| 538 | Traumatic acid | metabolite | M16 |
| 539 | PG(18:0/18:1) | metabolite | M16 |
| 540 | Canthaxanthin | metabolite | M7 |
| 541 | PG(18:1/18:2) | metabolite | M13 |
| 542 | LysoPC(22:4(7Z,10Z,13Z,16Z)) | metabolite | M14 |
| 543 | Precocene II | metabolite | M11 |
| 544 | Parakmerin A | metabolite | M11 |
| 545 | LysoPC(20:2(11Z,14Z)) | metabolite | M13 |
| 546 | 2,2,6,7-Tetramethylbicyclo[4.3.0]nona-1(9),4-diene-7,8-diol | metabolite | M5 |
| 547 | Cer/NS(d18:2/22:0) | metabolite | M17 |
| 548 | Stearoylethanolamide | metabolite | M16 |
| 549 | [7]-Paradol | metabolite | M14 |
| 550 | L-Norleucine | metabolite | M16 |
| 551 | 5-Amino-3-oxohexanoate | metabolite | M16 |
| 552 | Tangeretin | metabolite | M2 |
| 553 | LysoPI(18:0/0:0) | metabolite | M2 |
| 554 | PI(16:0/20:4) | metabolite | M13 |
| 555 | Docosapentaenoic acid (22n-3) | metabolite | M15 |
| 556 | (10)-Gingerol | metabolite | M8 |
| 557 | OxPI(18:1/18:1+3O) | metabolite | M13 |
| 558 | 2-Deoxycastasterone | metabolite | M8 |
| 559 | 12,13-DHOME | metabolite | M3 |
| 560 | PI(18:0/20:3) | metabolite | M13 |
| 561 | PE(22:6e/18:1) | metabolite | M16 |
| 562 | Suberic acid | metabolite | M16 |
| 563 | PI(18:0/18:1) | metabolite | M13 |
| 564 | PI(16:0/16:1) | metabolite | M13 |
| 565 | PI(16:0/18:1) | metabolite | M13 |
| 566 | PE(20:5(5Z,8Z,11Z,14Z,17Z)/22:5(7Z,10Z,13Z,16Z,19Z)) | metabolite | M9 |
| 567 | Uracil | metabolite | M17 |
| 568 | Epinephrine | metabolite | M2 |
| 569 | PC(17:0/18:2) | metabolite | M16 |
| 570 | Ajocysteine | metabolite | M3 |
| 571 | OxPI(18:0/18:1+3O) | metabolite | M13 |
| 572 | 8-hydroxy-6,7-dimethoxy-2H-chromen-2-one | metabolite | M16 |
| 574 | Phenylalanylphenylalanine | metabolite | M17 |
| 575 | ACar(12:1) | metabolite | M15 |
| 576 | PI(18:1/18:2) | metabolite | M13 |
| 577 | PS(20:5(5Z,8Z,11Z,14Z,17Z)/15:0) | metabolite | M9 |
| 578 | PI(18:0/20:2) | metabolite | M13 |
| 579 | 1-Pyrroline-5-carboxylic acid | metabolite | M2 |
| 580 | Prostaglandin D1 | metabolite | M12 |
| 581 | PI(18:0/18:2) | metabolite | M13 |
| 582 | Methylimidazole acetaldehyde | metabolite | M3 |
| 583 | FAHFA(2:0/22:2) | metabolite | M8 |
| 584 | Isoplumbagin | metabolite | M16 |
| 585 | Fagomine | metabolite | M2 |
| 586 | 5-Hydroxytryptophan | metabolite | M2 |
| 587 | 12,13-EpOME | metabolite | M8 |
| 588 | Norpropoxyphene | metabolite | M3 |
| 589 | 13S-hydroxyoctadecadienoic acid | metabolite | M2 |
| 590 | 3-Isoxazolidinone | metabolite | M11 |
| 591 | 2-Deoxybrassinolide | metabolite | M8 |
| 592 | Creatinine | metabolite | M16 |
| 593 | alpha-Ketoisovaleric acid | metabolite | M17 |
| 594 | Momordol | metabolite | M8 |
| 595 | PI(18:0/22:4) | metabolite | M13 |
| 596 | Glutaric acid | metabolite | M11 |
| 597 | LPI(18:2) | metabolite | M13 |
| 598 | Carbendazim | metabolite | M12 |
| 599 | 25-Hydroxycholesterol | metabolite | M9 |
| 600 | N-Hexadecanoylpyrrolidine | metabolite | M5 |
| 601 | PC(22:5(7Z,10Z,13Z,16Z,19Z)/16:1(9Z)) | metabolite | M16 |
| 602 | Arachidonic acid | metabolite | M15 |
| 603 | 1-Aminocyclopropanecarboxylic acid | metabolite | M9 |
| 604 | Inositol 1,3,4-trisphosphate | metabolite | M11 |
| 605 | L-Gulonic gamma-lactone | metabolite | M11 |
| 606 | (1S,2S,4R,8R)-p-Menthane-1,2,9-triol | metabolite | M5 |
| 607 | 6-Hydroxynicotinic acid | metabolite | M4 |
| 608 | Methyl (2E,6Z)-dodecadienoate | metabolite | M5 |
| 609 | Flavidin | metabolite | M16 |
| 610 | PI(18:0/20:4) | metabolite | M13 |
| 611 | Stigmasterol | metabolite | M5 |
| 612 | 7-Ketocholesterol | metabolite | M12 |
| 613 | N-a-Acetylcitrulline | metabolite | M11 |
| 614 | Demethyl-Coclaurine | metabolite | M11 |
| 615 | 5-Aminolevulinic acid | metabolite | M11 |
| 616 | PC(18:4e/2:0) | metabolite | M13 |
| 617 | β-Alanine | metabolite | M2 |
| 618 | Acrylamide | metabolite | M16 |
| 619 | alpha-Tocopherol succinate | metabolite | M5 |
| 620 | PC(18:5e/2:0) | metabolite | M13 |
| 621 | 5,8,11-Eicosatrienoic acid | metabolite | M15 |
| 622 | 1H-Indole-2,3-dione | metabolite | M16 |
| 623 | LysoPC(18:0) | metabolite | M16 |
| 624 | LPC(12:0) | metabolite | M13 |
| 625 | PI(16:0/18:2) | metabolite | M13 |
| 626 | PI(17:0/18:1) | metabolite | M13 |
| 627 | N-Nitroso-pyrrolidine | metabolite | M12 |
| 628 | DAG(18:2/18:2) | metabolite | M17 |
| 629 | PC(18:5e/6:0) | metabolite | M2 |
| 630 | Behenic acid | metabolite | M16 |
| 631 | LPC(19:0) | metabolite | M13 |
| 632 | Abscisic acid | metabolite | M5 |
| 633 | Zearalenone | metabolite | M5 |
| 634 | Cholic acid | metabolite | M12 |
| 635 | Palmitoleoyl Ethanolamide | metabolite | M5 |
| 636 | p-Menth-1-en-9-ol acetate | metabolite | M3 |
| 637 | Galanthaminone | metabolite | M11 |
| 638 | LPI(20:3) | metabolite | M14 |
| 639 | Chavicol | metabolite | M6 |
| 640 | Methionyl-Isoleucine | metabolite | M11 |
| 641 | PI(16:0/20:5) | metabolite | M13 |
| 642 | LysoPE(20:1(11Z)/0:0) | metabolite | M13 |
| 643 | PC(14:1e/6:0) | metabolite | M13 |
| 644 | 3-Hydroxy-6,8-dimethoxy-7(11)-eremophilen-12,8-olide | metabolite | M11 |
| 645 | N-Oleoylethanolamine | metabolite | M16 |
| 646 | L-alpha-Aspartyl-L-hydroxyproline | metabolite | M4 |
| 647 | Cassiachromone | metabolite | M17 |
| 648 | (3beta,5alpha,6beta,7alpha,22E,24R)-Ergosta-8,22-diene-3,5,6,7-tetrol | metabolite | M8 |
| 649 | S-(2-carboxypropyl)-Cysteamine | metabolite | M7 |
| 650 | Palmitoylethanolamide | metabolite | M17 |
| 651 | LPI(18:1) | metabolite | M13 |
| 652 | 8-Deoxy-11,13-dihydroxygrosheimin | metabolite | M6 |
| 653 | Petroselinic acid | metabolite | M15 |
| 654 | (-)-Isocorypalmine | metabolite | M11 |
| 655 | 3-Methyl-2-oxovaleric acid | metabolite | M4 |
| 656 | Homoarecoline | metabolite | M5 |
| 657 | LPC(20:5) | metabolite | M14 |
| 658 | Cyromazine | metabolite | M17 |
| 659 | Acetylleucine | metabolite | M11 |
| 660 | (10betaH,11xi)-11-Hydroxy-13-nor-6-eremophilen-8-one | metabolite | M8 |
| 661 | Methylsuccinic acid | metabolite | M3 |
| 662 | PI(16:1/18:2) | metabolite | M13 |
| 664 | 3-(3,4-Dihydroxy-5-methoxy)-2-propenoic acid | metabolite | M11 |
| 665 | N-[(4E,8E)-1,3-dihydroxyoctadeca-4,8-dien-2-yl]hexadecanamide | metabolite | M5 |
| 666 | 5-Aminopentanamide | metabolite | M2 |
| 667 | (3beta,5alpha,6alpha,9alpha,22E,24R)-Ergosta-7,22-diene-3,5,6,9-tetrol | metabolite | M8 |
| 668 | 3,4-Dihydrocoumarin | metabolite | M2 |
| 669 | 2,4-Pentadienal | metabolite | M11 |
| 670 | Picrotoxinin | metabolite | M12 |
| 671 | L-Methionine | metabolite | M2 |
| 672 | Isopropyl formate | metabolite | M3 |
| 673 | Allysine | metabolite | M3 |
| 674 | Hexadecanedioic acid | metabolite | M12 |
| 675 | HexCer/NS(d18:1/16:0) | metabolite | M17 |
| 676 | Pyruvic acid | metabolite | M3 |
| 677 | 8-Hydroxy-deoxyguanosine | metabolite | M15 |
| 678 | FAHFA(18:2/18:0) | metabolite | M2 |
| 679 | 4-Butyl-gamma-butyrolactone | metabolite | M2 |
| 680 | Cer/NS(d18:2/24:1) | metabolite | M17 |
| 681 | Stearidonic acid | metabolite | M15 |
| 682 | Myristoleic acid | metabolite | M15 |
| 683 | Galactosylsphingosine | metabolite | M13 |
| 684 | 7-Methylguanine | metabolite | M17 |
| 685 | trans-Zeatin-riboside | metabolite | M17 |
| 686 | cis-5-Tetradecenoylcarnitine | metabolite | M15 |
| 687 | 4-Pentenoic acid | metabolite | M3 |
| 688 | Indolepyruvate | metabolite | M4 |
| 689 | Ethyl (2E,6Z)-dodecadienoate | metabolite | M8 |
| 690 | Farnesol | metabolite | M1 |
| 691 | PGD2 ethanolamide | metabolite | M7 |
| 692 | 27-Norcholestanehexol | metabolite | M13 |
| 693 | Sphingosine | metabolite | M9 |
| 694 | Phthalic acid | metabolite | M10 |
| 695 | Thiacremonone | metabolite | M9 |
| 696 | (3R,7R)-1,3,7-Octanetriol | metabolite | M4 |
| 697 | Dihydrojasmonic acid | metabolite | M12 |
| 698 | N-Desmethyltramadol | metabolite | M11 |
| 699 | 7-Ketodeoxycholic acid | metabolite | M8 |
| 700 | (3beta,8beta)-3-Hydroxy-7(11)-eremophilen-12,8-olide | metabolite | M12 |
| 701 | Artomunoxanthentrione | metabolite | M16 |
| 702 | Dihydrofolic acid | metabolite | M12 |
| 703 | Octopamine | metabolite | M9 |
| 704 | 13,14-Dihydro PGE1 | metabolite | M12 |
| 705 | Palmitic acid | metabolite | M6 |
| 706 | 1,8-Octanedithiol | metabolite | M16 |
| 707 | LysoPC(P-16:0) | metabolite | M13 |
| 708 | Hydrocortisone | metabolite | M12 |
| 709 | 9,10-DHOME | metabolite | M7 |
| 710 | Daidzein | metabolite | M11 |
| 711 | 2-(3-Phenylpropyl)pyridine | metabolite | M11 |
| 712 | (-)-Thebaine | metabolite | M11 |
| 713 | Norambreinolide | metabolite | M8 |
| 714 | 6-Methyltetrahydropterin | metabolite | M9 |
| 715 | 3,4-Dihydroxymandelic acid | metabolite | M16 |
| 716 | Xanthohumol | metabolite | M15 |
| 717 | Pyroglutamic acid | metabolite | M5 |
| 718 | Isopropyl beta-D-glucoside | metabolite | M11 |
| 719 | IS4 | metabolite | M11 |
| 720 | 3-Octanol glucoside | metabolite | M5 |
| 722 | Zingerone | metabolite | M16 |
| 723 | Dehydroxypaxilline | metabolite | M11 |
| 724 | 1-Methylhistamine | metabolite | M16 |
| 725 | Hydroxyisocaproic acid | metabolite | M4 |
| 726 | Hypogeic acid | metabolite | M8 |
| 727 | alpha-Tocopherolquinone | metabolite | M5 |
| 728 | beta-Cryptoxanthin | metabolite | M17 |
| 729 | N-Nitrosothiazolidine-4-carboxylic acid | metabolite | M11 |
| 730 | 3-Phenoxybenzoic acid | metabolite | M11 |
| 731 | LysoSM(d18:1) | metabolite | M2 |
| 732 | Tabernanthine | metabolite | M5 |
| 733 | Mangiferdesmethylursanone | metabolite | M17 |
| 734 | (E)-4'-Methylresveratrol 3-glucoside | metabolite | M11 |
| 735 | Ethyl stearate | metabolite | M15 |
| 736 | Mesobilirubinogen | metabolite | M10 |
| 737 | Sepiapterin | metabolite | M3 |
| 738 | Secoclausenamide | metabolite | M3 |
| 739 | IS6 | metabolite | M11 |
| 740 | Ganoderic acid B | metabolite | M11 |
| 741 | PC(20:4(5Z,8Z,11Z,14Z)/18:0) | metabolite | M12 |
| 742 | PS(20:5(5Z,8Z,11Z,14Z,17Z)/14:1(9Z)) | metabolite | M9 |
| 743 | Leukotriene E3 | metabolite | M9 |
| 744 | Aniline | metabolite | M1 |
| 745 | Hydroxyoctanoic acid | metabolite | M12 |
| 746 | Succinic acid | metabolite | M9 |
| 747 | Tetracosahexaenoic acid | metabolite | M12 |
| 748 | Niacinamide | metabolite | M3 |
| 749 | Dihydro-4-mercapto-3(2H)-furanone | metabolite | M9 |
| 750 | Heptanoic acid | metabolite | M3 |
| 751 | Glycerol triundecanoate | metabolite | M8 |
| 752 | Ethyl 2,4-dioxohexanoate | metabolite | M11 |
| 753 | MG(22:5(7Z,10Z,13Z,16Z,19Z)/0:0/0:0) | metabolite | M3 |
| 754 | Juglone | metabolite | M4 |
| 755 | 16(R)-HETE | metabolite | M12 |
| 756 | Methyl methacrylate | metabolite | M11 |
| 757 | 3,4-Dihydroxybenzylamine | metabolite | M4 |
| 758 | (alpha-D-mannosyl)7-beta-D-mannosyl-diacetylchitobiosyl-L-asparagine, isoform A (protein) | metabolite | M2 |
| 759 | Acetyl tributyl citrate | metabolite | M12 |
| 760 | alpha-Butyl-omega-hydroxypoly(oxyethylene) poly(oxypropylene) | metabolite | M9 |
| 761 | 3-Methyl-2-(1-pyrrolidinyl)-2-cyclopenten-1-one | metabolite | M2 |
| 762 | Rotenone | metabolite | M11 |
| 763 | 3-Oxocholic acid | metabolite | M8 |
| 764 | 3-Dehydroxycarnitine | metabolite | M12 |
| 765 | Ubiquinone-1 | metabolite | M7 |
| 766 | Ethyl tetradecanoate | metabolite | M16 |
| 767 | Glycerol 1-(5-hydroxydodecanoate) | metabolite | M5 |
| 768 | Dehydroepiandrosterone sulfate | metabolite | M3 |
| 769 | Ginkgoic acid | metabolite | M12 |
| 770 | N,N-Dimethylaniline | metabolite | M17 |
| 771 | Neryl propionate | metabolite | M7 |
| 772 | (R)-Heraclenol 2'-(3-methylbutanoate) | metabolite | M5 |
| 773 | 3-Hydroxysebacic acid | metabolite | M11 |
| 774 | (4E)-1,7-bis(4-hydroxyphenyl)hept-4-en-3-one | metabolite | M11 |
| 775 | Ixocarpanolide | metabolite | M9 |
| 776 | Prostaglandin E2 | metabolite | M10 |
| 777 | Ganoderiol H | metabolite | M8 |
| 778 | 15-HPETE | metabolite | M12 |
| 779 | Isoacitretin | metabolite | M12 |
| 780 | Hydroxycotinine | metabolite | M7 |
| 781 | 9,10-epoxyoctadecanoic acid | metabolite | M12 |
| 782 | Eremopetasidione | metabolite | M7 |
| 783 | Glutamylthreonine | metabolite | M12 |
| 784 | LysoPE(20:2(11Z,14Z)/0:0) | metabolite | M13 |
| 786 | LysoPC(14:1(9Z)) | metabolite | M14 |
| 787 | Sebacic acid | metabolite | M16 |
| 788 | 4-Oxo-2-nonenal | metabolite | M5 |
| 789 | Estrone | metabolite | M7 |
| 790 | Hebevinoside VI | metabolite | M9 |
| 791 | Jangomolide | metabolite | M9 |
| 792 | 5a-Tetrahydrocorticosterone | metabolite | M8 |
| 793 | Baccatin III | metabolite | M17 |
| 794 | Hyodeoxycholic acid | metabolite | M17 |
| 795 | Pentadecanoic acid | metabolite | M4 |
| 796 | 2-Hydroxyxanthone | metabolite | M16 |
| 797 | Capric acid | metabolite | M15 |
| 798 | 2-Methyl-3-hydroxyvaleric acid | metabolite | M11 |
| 799 | DG(18:4(6Z,9Z,12Z,15Z)/15:0/0:0) | metabolite | M8 |
| 800 | 8-hydroxy-7-methoxy-3-(2-methylbut-3-en-2-yl)-2H-chromen-2-one | metabolite | M4 |
| 801 | 2-Propylpiperidine | metabolite | M11 |
| 802 | Sarcodon scabrosus Depsipeptide | metabolite | M16 |
| 803 | 2-Methylglutaric acid | metabolite | M16 |
| 804 | (3beta,5beta,8beta,22E,24xi)-Ergosta-6,22-diene-3,5,8-triol | metabolite | M5 |
| 805 | Isosakuranin | metabolite | M6 |
| 806 | (Â±)-4-Methylene-2-pyrrolidinecarboxylic acid | metabolite | M11 |
| 807 | Ruscogenin | metabolite | M16 |
| 808 | gamma-Camphorene | metabolite | M5 |
| 809 | N-Methyl-1H-indole-3-propanamide | metabolite | M4 |
| 810 | [12]-Gingerol | metabolite | M15 |
| 811 | Taurodeoxycholic acid | metabolite | M12 |
| 812 | Ethyl hexadecanoate | metabolite | M3 |
| 813 | Hexadecanedioic acid mono-L-carnitine ester | metabolite | M12 |
| 814 | Enoxolone | metabolite | M3 |
| 815 | 14alpha-Hydroxypaxilline | metabolite | M11 |
| 816 | Nervonoylacetone | metabolite | M8 |
| 817 | Octyl phenylacetate | metabolite | M8 |
| 818 | (-)-(E)-1-(4-Hydroxyphenyl)-7-phenyl-6-hepten-3-ol | metabolite | M11 |
| 819 | Artesunate | metabolite | M5 |
| 820 | 4'-Hydroxyacetophenone | metabolite | M16 |
| 821 | Octadecylamine | metabolite | M11 |
| 822 | Rubitic acid | metabolite | M8 |
| 823 | Kahweol | metabolite | M16 |
| 824 | Arginyl-Alanine | metabolite | M16 |
| 825 | Calcitriol | metabolite | M5 |
| 826 | Cortisol | metabolite | M12 |
| 827 | Dopamine | metabolite | M2 |
| 828 | 2-O-p-Coumaroylhydroxycitric acid | metabolite | M16 |
| 829 | 5-Oxooctadecanoic acid | metabolite | M17 |
| 830 | Dehydroxyzyleuton | metabolite | M16 |
| 831 | Oleacein | metabolite | M11 |
| 832 | Phytosphingosine | metabolite | M6 |
| 833 | 3-Isopropenylpentanedioic acid | metabolite | M16 |
| 834 | Acesulfame | metabolite | M2 |
| 835 | 2-Cinnamoyl-1-galloyl-beta-D-glucopyranose | metabolite | M16 |
| 836 | Estriol | metabolite | M5 |
| 837 | N1-Methyl-2-pyridone-5-carboxamide | metabolite | M10 |
| 838 | Ovalicin | metabolite | M5 |
| 839 | 1,2-Dihydro-1,1,6-trimethylnaphthalene | metabolite | M5 |
| 840 | 4a-Carboxy-4b-methyl-5a-cholesta-8,24-dien-3b-ol | metabolite | M5 |
| 841 | p-Mentha-1,8-dien-7-ol | metabolite | M11 |
| 842 | 20-HDoHE | metabolite | M15 |
| 843 | Mizolastine | metabolite | M11 |
| 844 | rac-Normetanephrine | metabolite | M11 |
| 845 | (2R,3R)-2,3-Butanediol | metabolite | M17 |
| 846 | Pipericine | metabolite | M16 |
| 847 | 1-Palmitoylglycerophosphoinositol | metabolite | M13 |
| 848 | Aspartyl-Glutamate | metabolite | M11 |
| 849 | 2-Hydroxystearic acid | metabolite | M12 |
| 850 | 7,12-Diketolithocholic acid | metabolite | M8 |
| 851 | Sphinganine | metabolite | M6 |
| 852 | 3-Methyl-5-pentyl-2-furanundecanoic acid | metabolite | M13 |
| 853 | Saccharopine | metabolite | M7 |
| 854 | Prostaglandin B1 | metabolite | M16 |
| 855 | Nicotinic acid | metabolite | M14 |
| 856 | 2-Phenylethyl octanoate | metabolite | M8 |
| 857 | LysoPC(18:3(9Z,12Z,15Z)) | metabolite | M13 |
| 858 | 20-Hydroxyecdysone | metabolite | M12 |
| 859 | 5,7-dihydroxy-2-phenyl-6-[3,4,5-trihydroxy-6-(hydroxymethyl)oxan-2-yl]-8-(3,4,5-trihydroxyoxan-2-yl)-4H-chromen-4-one | metabolite | M9 |
| 860 | Schidigeragenin B | metabolite | M12 |
| 861 | Melleolide H | metabolite | M6 |
| 862 | Stenocereol | metabolite | M5 |
| 863 | 8(R)-Hydroperoxylinoleic acid | metabolite | M12 |
| 864 | Acetaldehyde butyl phenethyl acetal | metabolite | M8 |
| 865 | 5-Methylcytidine | metabolite | M4 |
| 866 | 4-Acetamido-2-amino-6-nitrotoluene | metabolite | M5 |
| 867 | LysoPE(0:0/20:4(5Z,8Z,11Z,14Z)) | metabolite | M13 |
| 868 | 2'-Deoxycytidine | metabolite | M12 |
| 869 | Ricinoleic acid | metabolite | M9 |
| 870 | Avenestergenin B2 | metabolite | M11 |
| 871 | Undecanoic acid | metabolite | M11 |
| 872 | (9S,10S)-9,10-dihydroxyoctadecanoate | metabolite | M8 |
| 873 | Enkephalin L | metabolite | M13 |
| 874 | AlisolA24-acetate | metabolite | M16 |
| 875 | Cyanidin | metabolite | M11 |
| 876 | Pinostrobin | metabolite | M4 |
| 877 | Kanzonol K | metabolite | M6 |
| 878 | 6-(2-Carboxyethyl)-7-hydroxy-2,2-dimethyl-4-chromanone glucoside | metabolite | M7 |
| 879 | Isovalerylglutamic acid | metabolite | M11 |
| 880 | Arginyl-Proline | metabolite | M11 |
| 881 | 7,8-Dihydropteroic acid | metabolite | M11 |
| 882 | (3xi,6E)-1,7-Diphenyl-6-hepten-3-ol | metabolite | M5 |
| 883 | (3beta,5alpha,6alpha,7beta,14alpha,22E,24R)-5,6-Epoxyergosta-8,22-diene-3,7,14-triol | metabolite | M8 |
| 884 | PC(22:5(7Z,10Z,13Z,16Z,19Z)/20:2(11Z,14Z)) | metabolite | M17 |
| 885 | 3-Methyl-histidine | metabolite | M12 |
| 886 | (all-E)-Rubixanthin | metabolite | M16 |
| 887 | 8beta-Angeloyloxy-15-hydroxy-1alpha,10R-dimethoxy-3-oxo-11(13)-germacren-12,6alpha-olide | metabolite | M11 |
| 888 | Isopeonidin 3-rutinoside | metabolite | M3 |
| 889 | (3R,8E)-3-Hydroxy-5,8-megastigmadien-7-one | metabolite | M5 |
| 890 | 2-(acetylamino)-1,5-anhydro-2-deoxy-3-O-b-D-galactopyranosyl-D-arabino-Hex-1-enitol | metabolite | M11 |
| 891 | 3-Aminoisobutanoic acid | metabolite | M17 |
| 892 | 1,2,3,4-Tetramethoxy-5-(2-propenyl)benzene | metabolite | M11 |
| 893 | Glutamylglutamic acid | metabolite | M11 |
| 894 | Ethyl Arachidonate | metabolite | M11 |
| 895 | Cholesterol sulfate | metabolite | M13 |
| 896 | Dihydroxyacetone Phosphate Acyl Ester | metabolite | M2 |
| 897 | Glutaminylarginine | metabolite | M16 |
| 898 | 6-Deoxocastasterone | metabolite | M6 |
| 899 | Brassinolide | metabolite | M13 |
| 900 | L-Aspartyl-4-phosphate | metabolite | M12 |
| 901 | 3-Oxo-5β-cholanoic acid | metabolite | M9 |
| 902 | Lansiumamide C | metabolite | M11 |
| 903 | Sudachiin B | metabolite | M16 |
| 904 | (3'alpha,5'alpha,9'xi,10'beta)-O-(3-Hydroxy-7-drimen-11-yl)umbelliferone | metabolite | M5 |
| 905 | Resolvin D2 | metabolite | M15 |
| 906 | Quinolinic acid | metabolite | M17 |
| 907 | Maslinic acid | metabolite | M8 |
| 908 | Eicosadienoic acid | metabolite | M3 |
| 909 | Methyl jasmonate | metabolite | M7 |
| 910 | gamma-Calacorene | metabolite | M11 |
| 911 | Coprocholic acid | metabolite | M3 |
| 912 | Cer(d18:1/14:0) | metabolite | M5 |
| 913 | Polyporusterone E | metabolite | M8 |
| 914 | Apo-12'-violaxanthal | metabolite | M5 |
| 915 | Octadecanedioic acid | metabolite | M12 |
| 916 | PC(22:6(4Z,7Z,10Z,13Z,16Z,19Z)/22:6(4Z,7Z,10Z,13Z,16Z,19Z)) | metabolite | M13 |
| 917 | Persicaxanthin | metabolite | M15 |
| 918 | Lysyl-Histidine | metabolite | M11 |
| 919 | Norepinephrine sulfate | metabolite | M3 |
| 920 | Uridine 5'-diphosphate (UDP) | metabolite | M11 |
| 921 | Ginkgolide A | metabolite | M8 |
| 922 | N-Methylhistamine | metabolite | M14 |
| 923 | (-)-11-Hydroxy-9,15,16-trioxooctadecanoic acid | metabolite | M11 |
| 924 | MG(0:0/20:3(11Z,14Z,17Z)/0:0) | metabolite | M3 |
| 925 | CPA(18:1(11Z)/0:0) | metabolite | M17 |
| 926 | N-Stearoylsphingosine | metabolite | M16 |
| 927 | Cannabidiol | metabolite | M15 |
| 928 | Fasciculic acid B | metabolite | M11 |
| 929 | 2-Acetylpyrrolidine | metabolite | M11 |
| 930 | Diethylphosphate | metabolite | M11 |
| 931 | (10E,12Z)-9-HODE | metabolite | M15 |
| 932 | Thalicpureine | metabolite | M11 |
| 933 | 4-Hydroxy-3-(3-methyl-2-butenyl)acetophenone | metabolite | M2 |
| 934 | Girinimbine | metabolite | M7 |
| 935 | Succinic anhydride | metabolite | M3 |
| 936 | Armillarin | metabolite | M6 |
| 937 | 1-(2,6,6-Trimethyl-1-cyclohexen-1-yl)-1-penten-3-one | metabolite | M8 |
| 938 | Prostaglandin F1a | metabolite | M9 |
| 939 | Fexofenadine | metabolite | M14 |
| 940 | 2,6-Di-tert-butyl-1,4-benzenediol | metabolite | M17 |
| 941 | DG(18:3(9Z,12Z,15Z)/15:0/0:0) | metabolite | M8 |
| 942 | gamma-L-Glutamyl-gamma-L-glutamyl-L-methionine | metabolite | M2 |
| 943 | Glycocholic acid | metabolite | M13 |
| 944 | Isoliquiritigenin | metabolite | M5 |
| 945 | Linoleoyl ethanolamide | metabolite | M5 |
| 946 | 3-Hydroxyanthranilic acid | metabolite | M12 |
| 947 | Rutarin | metabolite | M11 |
| 948 | N-Acetyl-5-hydroxytryptamine | metabolite | M4 |
| 949 | Octadecanamide | metabolite | M5 |
| 950 | Perfluorooctanoic acid | metabolite | M16 |
| 951 | 2-(5,8-Tetradecadienyl)cyclobutanone | metabolite | M13 |
| 952 | CPA(18:2(9Z,12Z)/0:0) | metabolite | M13 |
| 953 | Marmesin | metabolite | M11 |
| 954 | 2-Propanoylthiazole | metabolite | M17 |
| 955 | 5-Hydroxy-6-methoxycoumarin 7-glucoside | metabolite | M16 |
| 956 | Dioscoretine | metabolite | M3 |
| 957 | Geranyl acetate | metabolite | M5 |
| 958 | 3-Oxo-12,18-ursadien-28-oic acid | metabolite | M17 |
| 959 | (3S,3'R,5R,6R)-7',8'-Didehydro-3,6-epoxy-5,6-dihydro-beta,beta-carotene-3',5-diol | metabolite | M3 |
| 960 | (3beta,22R,23R,24S)-3,22,23-Trihydroxystigmastan-6-one | metabolite | M3 |
| 961 | N-[(4E,8Z)-1,3-dihydroxyoctadeca-4,8-dien-2-yl]hexadecanamide 1-glucoside | metabolite | M17 |
| 962 | Ganoderic acid eta | metabolite | M12 |
| 963 | Ambolic acid | metabolite | M11 |
| 964 | D-Alanine | metabolite | M2 |
| 965 | MG(0:0/15:0/0:0) | metabolite | M17 |
| 966 | Lithocholic acid glycine conjugate | metabolite | M11 |
| 967 | 8-Hydroxyoctanoate | metabolite | M5 |
| 968 | N-3-Methyluridine | metabolite | M12 |
| 969 | Selenomethionine | metabolite | M11 |
| 970 | 2-benzoylbenzene-1,3,5-triol | metabolite | M11 |
| 971 | Lansamide 3 | metabolite | M11 |
| 972 | (6E)-Piperamide-C7:1 | metabolite | M11 |
| 973 | Vitamin K1 2,3-epoxide | metabolite | M12 |
| 974 | Dethiobiotin | metabolite | M10 |
| 975 | Momordenol | metabolite | M5 |
| 976 | L-trans-alpha-Amino-2-carboxycyclopropaneacetic acid | metabolite | M12 |
| 977 | Torvoside G | metabolite | M11 |
| 978 | Caprylic acid | metabolite | M17 |
| 979 | (E)-Squamosamide | metabolite | M6 |
| 980 | 5-Hexyltetrahydro-2-oxo-3-furancarboxylic acid | metabolite | M5 |
| 981 | 2-Acetoxy-3-geranylgeranyl-1,4-dihydroxybenzene | metabolite | M11 |
| 982 | Normetanephrine | metabolite | M7 |
| 983 | 20-Hydroxy-PGE2 | metabolite | M16 |
| 984 | 2,6-Toluenediamine | metabolite | M4 |
| 985 | (-)-Wikstromol | metabolite | M11 |
| 986 | Allyl cyclohexylacetate | metabolite | M11 |
| 987 | Terbutaline | metabolite | M5 |
| 988 | Physangulide | metabolite | M16 |
| 989 | 2-Isopropyl-3-oxosuccinate | metabolite | M16 |

Note: All proteins are named by retrieving annotations from the UniProt website (<https://www.uniprot.org>). All metabolites are named according to reference databases from the MetaboAnalyst website ([https://www.metaboanalyst.ca](https://www.metaboanalyst.ca/)).
